# Comparative genomics and *in silico* virulence assessment of nonpathogenic *Avibacterium paragallinarum* isolates from healthy layer chickens

**DOI:** 10.3389/fmicb.2026.1919963

**Published:** 2026-09-03

**Authors:** Mostafa M. S. Shelkamy, Stephan Schmitz-Esser, Amro Hashish, Eman Gadu, Maria Chaves, Yuko Sato, Mohamed El-Gazzar

**Affiliations:** 1 Department of Veterinary Diagnostic and Production Animal Medicine, College of Veterinary Medicine, Iowa State University, Ames, IA, United States; 2 Department of Avian and Rabbit Medicine, Faculty of Veterinary Medicine, Suez Canal University, Ismailia, Egypt; 3 Department of Animal Science, Iowa State University, Ames, IA, United States; 4 Department of Veterinary and Biomedical Sciences, South Dakota State University, Brookings, SD, United States; 5 National Laboratory for Veterinary Quality Control on Poultry Production, Animal Health Research Institute, Agriculture Research Center, Giza, Egypt; 6 Department of Avian and Rabbit Diseases, Faculty of Veterinary Medicine, Mansoura University, Mansoura, Egypt

**Keywords:** *Avibacterium paragallinarum*, comparative genomics, infectious coryza, nonpathogenic, virulence factors

## Abstract

Infectious coryza (IC) is a respiratory disease of chickens caused by the bacterial primary pathogen *Avibacterium paragallinarum* (AP). Clinical signs are characteristic and suggestive of the disease; however, diagnosis is confirmed through bacterial isolation or qPCR detection. Recently, several naïve, healthy layer (NHL) flocks, neither exposed to IC nor vaccinated against it, tested positive using current IC-specific qPCR assays. Moreover, AP isolates were recovered from these NHL flocks and were designated “non-pathogenic *Avibacterium paragallinarum*” (npAP) due to the absence of clinical signs in the field and in an experimental challenge study. However, understanding the genetic basis behind this difference in pathogenicity remains unknown. Therefore, the purpose of the current study was to investigate the genetic explanations for the lack of pathogenicity in npAP. Comprehensive comparative genomic analysis was performed for 14 npAP and 87 pathogenic AP (pAP) genomes at three main levels, (a) gene presence/absence, (b) sequence variation within translated open reading frames (ORFs) across the whole genome, and (c) targeted analysis of selected virulence-associated genes. The analysis revealed four consistent features, (1) The *HMTp210* gene, encoding the trimeric autotransporter adhesin HMTp210, important for serotyping and protective immunity, is 2–3 times longer in npAP compared to pAP. (2) npAP isolates exhibit either complete absence of the capsular polysaccharide locus or defective genes within the locus. (3) All npAP isolates lack both *hmp* and *nsrR*, which encode Flavohemoglobin/Nitric oxide dioxygenase protein (Hmp) and the nitrite-sensitive transcriptional repressor protein (NsrR), respectively. (4) The *ftsZ* gene, which is essential for cell-division, exhibits substantial allelic divergence between npAP and pAP. We hypothesize that defects or changes in HMTp210 adhesin, capsule, the nitric oxide dioxygenase system, or FtsZ contribute to the nonpathogenic phenotype of npAP in chickens. Moreover, most of npAP isolates encode a putative sialylated lipooligosaccharide which may promote a more commensal-like interaction with the host immune system, preventing an overt inflammatory response. In conclusion, our findings highlight potential virulence factors that can be involved in AP pathogenesis. Additionally, defining and understanding the role of these potential virulence factors, may open new avenues for the development of modified live vaccines against IC.

## 1 Introduction

*Avibacterium paragallinarum* (AP) is a primary bacterial pathogen of chickens (Blackall et al., 2005). It causes infectious coryza (IC), a well-known disease of economic importance. IC is characterized by upper respiratory clinical signs, facial swelling, reduced water and feed intake, and consequently, up to a 40% decline in egg production in laying hens, as well as growth retardation in broilers (Blackall, 1999). Historically, IC is reported to be endemic in warm weather geographies and rarely if ever reported in cold weather geographies. In the US, the disease has been endemic in southern states such as Florida, Texas, and California (Dunn et al., 2003). However, several IC outbreaks have been witnessed starting in Pennsylvania then spreading to Ohio, Indiana and eventually to Iowa since December 2018 (El-Gazzar et al., 2024; Shelkamy et al., 2024). This marked the first time IC had ever been documented in these northern states. Therefore, clinical presentation of the initial cases in each state was severe, as the affected flocks were naïve to AP infection.

In an effort to contain the outbreaks, investigations were first conducted around the index case in Ohio, where samples from naïve healthy layer (NHL) flocks were collected and tested using AP-specific qPCR assays (Corney et al., 2008; Kuchipudi et al., 2021). Surprisingly, many of these NHL flocks tested positive despite the absence of clinical signs. At the same time, NHL flocks from Iowa, where the disease had not been reported yet, tested positive with the same AP-specific qPCR assays. After troubleshooting to confirm that there were no foreseen issues with the qPCR assays, investigation of the possibility of a variant AP population was conducted. Multiple isolation attempts were carried out from these qPCR-positive NHL flocks, and two AP isolates were successfully isolated and characterized: one from Ohio and the other from Iowa (Hashish et al., 2023). Preliminary genomic analyses of these isolates, compared with pathogenic AP (pAP), revealed genetic differences in major virulence factors such as *HMTp210* gene and the capsule biosynthesis locus (Hashish et al., 2023; Shelkamy et al., 2024). Because these isolates originated from NHL flocks that did not show any signs of disease, these AP isolates were designated as non-pathogenic (npAP).

Following the first isolation of npAP, efforts to address specific diagnostic gaps have been conducted and included the development and validation of differential qPCR assays to distinguish between pAP and npAP directly from clinical samples (Shelkamy et al., 2024). In addition, a pilot surveillance study was carried out to investigate the prevalence of npAP in the US commercial layer industry (Shelkamy et al., 2025a) in which, a total of 710 oropharyngeal swab pools were collected from 80 NHL chicken sites across 13 states and tested using both the screening qPCR assay (Kuchipudi et al., 2021) and the recently developed differential qPCR assays (Shelkamy et al., 2024). This pilot study revealed a high prevalence of npAP, estimated at 35% in the US commercial layer industry (Shelkamy et al., 2025a). During the pilot surveillance study, seventeen npAP isolates were obtained from six different events across four states (Shelkamy et al., 2025a). Twelve of these isolates were sequenced using Illumina and Oxford Nanopore Technologies (ONT), 8 complete and 4 draft genome sequences were obtained (Shelkamy et al., 2025b). The non-pathogenic nature of seven npAP isolates was evaluated during a live bird challenge study using the intra-sinus injection route and none of the isolates caused any pathology, both clinically and microscopically, in the challenged birds. The results reflected the field situation and provided strong evidence that npAP are indeed nonpathogenic (Shelkamy et al., 2026).

Several studies have investigated and attributed AP pathogenicity to key factors, including the trimeric autotransporter adhesin HMTp210 (Yamaguchi et al., 1993; Wang et al., 2014), the capsule (Sawata et al., 1985; Tu et al., 2015; Chen et al., 2024), cytotoxins such as RTX (Küng and Frey, 2013) and cytolethal distending toxins (CDT) (Chen et al., 2014), fimbriae (Liu et al., 2016), heme utilization systems (Huo et al., 2023), outer membrane vesicles (Ramón Rocha et al., 2006; Mei et al., 2020), and metalloproteases (Rivero-García et al., 2005).

After the discovery and characterization of npAP isolates, the logical next step is to investigate what these bacteria lack, or have, that renders them non-pathogenic and to identify the genetic basis for this attenuation. Are all, or some, of the previously described virulence factors non-functional or absent in npAP? Therefore, the main objectives of this study were: (i) to perform a general comparative genomic analysis between npAP and pAP to identify genetic differences between the two populations, and (ii) to examine in greater detail both well-characterized virulence factors in AP and additional predicted factors that may contribute to its pathogenicity or lack thereof. By narrowing the scope to specific virulence determinants and comparing them genetically between pAP and npAP, potential explanations for the lack of pathogenicity in npAP can be uncovered.

## 2 Materials and methods

In this study, comparative genomic analyses between npAP and pAP were conducted at three main levels: (1) gene presence/absence, (2) sequence variation within translated open reading frames (ORFs) across the whole genome, and (3) targeted analysis of selected virulence-associated genes. These analyses were performed in BV-BRC (Olson et al., 2023) using *in silico* translated ORF sequences. In addition, phylogenomic analysis using BV-BRC Global Protein Families (PGFams) (Davis et al., 2016), and phylogenetic analysis based on the *HMTp210* gene were conducted.

### 2.1 Isolates description and genomes included in the analysis

A total of 101 AP genomes were included in this study, comprising 14 npAP and 87 pAP isolates. Classification as npAP or pAP was primarily based on the case history, specifically whether the isolates were recovered from naïve healthy or diseased flocks. The 14 npAP isolates were obtained from naïve healthy layer chickens representing eight different events from seven sites and were sequenced using Illumina MiSeq and ONT MinION platforms (Hashish et al., 2023; Shelkamy et al., 2025b). The six earlier npAP isolates used for the selection of differential qPCR targets, as described by Shelkamy et al. (2024), were included in the set of 14 npAP genomes analyzed in this study. pAP genomes consisted of 82 publicly available assembled genomes retrieved from the NCBI Genomes database, and five additional clinical isolates submitted to Iowa State University Veterinary Diagnostic laboratory, which were sequenced using Illumina technology following the same methods as Shelkamy et al. (2025b). A detailed description of all genomes is provided in Supplementary Table 1. Quast version 5.2 was used to assess assembly quality (Gurevich et al., 2013). All genomes were annotated using the RAST tool kit (RASTtk) (Brettin et al., 2015).

### 2.2 Phylogenomics and phylogenetics

The Bacterial Phylogenetic Tree service in BV-BRC (Olson et al., 2023, Wattam et al., 2024) was used with the default settings to construct a core genome phylogenomic tree. A total of 557 core genes (Supplementary Table 2), present in 100% of the analyzed genomes, were selected from PGFams (Davis et al., 2016) to construct the phylogenomic tree. Genomes of npAP (*n* = 14), pAP (*n* = 82), and other *Avibacterium* species (*n* = 8) were included. The NCBI accession numbers for the *Avibacterium* species were JAKKOF000000000 (*Avibacterium* sp. 20–15), JAKKOD000000000 (*Avibacterium* sp. 21–586), PQVI00000000 (*Avibacterium endocarditidis* 20186H4H1), UGSP00000000 (*Avibacterium avium* strain NCTC 11297), PQVJ00000000 and UGSQ00000000 (*Avibacterium gallinarum* strain NCTC11188), SNXJ00000000 (*Avibacterium gallinarum* strain DSM 17481), and LR134167 (*Avibacterium volantium* strain NCTC3438).

To predict the serogroup of npAP isolates, a maximum-likelihood phylogenetic tree was constructed using the first 1,200 bp of the *HMTp210* gene from both npAP and pAP (Tan et al., 2021). The published dataset of sequences (Buter et al., 2023) was used to construct the phylogenetic tree. IQ-Tree multicore version 1.6.12 (Nguyen et al., 2014) was used, and the best-fit model (HKY+F+G4) was selected according to BIC. The ultrafast bootstrap was performed with 1,000 replicates (Minh et al., 2013). FigTree v1.4.4 (Rambaut, 2018) was used for trees visualization.

### 2.3 Comparative genomic analysis

#### 2.3.1 Gene presence/absence

To identify gene presence or absence in the npAP population compared to the pAP population, the Comparative Systems service in BV-BRC (Olson et al., 2023; Wattam et al., 2024) was used. Briefly, this service performs a pan-genome analysis with various filtering options that enable rapid identification of protein families conserved across 100% of the selected genomes (core genome) or conserved only in a subset (accessory genome) (Olson et al., 2023). Accordingly, in the present study, a protein family was defined as part of the core genome only if it was present in all genomes analyzed (100% threshold). We acknowledge that the unequal sample sizes (14 npAP vs. 87 pAP) may influence pan-genome partitioning. The Comparative Systems service primarily relies on *k*-mer based assignment of protein families (Davis et al., 2016).

The consistent absence of a gene or a group of genes in a particular population was further validated using NCBI Nucleotide BLAST (Align two or more sequences). A gene was considered present when a significant BLASTN hit was detected with ≥ 80% nucleotide sequence identity, ≥ 70% query coverage, and an E-value ≤ 1 × 10^−5^. For complete genome assemblies, these criteria were applied directly to determine gene presence or absence. For draft genome assemblies, the same thresholds were applied with caution, particularly when BLAST hits were distributed across multiple contigs. Such cases were reported and manually inspected by evaluating gene location, contig boundaries, and ORF integrity was performed to distinguish true gene absence from potential sequencing or assembly artifacts. When BLAST hits, with ≥ 80% nucleotide sequence identity, were located at the ends and beginnings of contigs, the genomic context, including neighboring genes and operon organization, was examined. If the fragmented hits were consistent with a single gene interrupted by a contig break, they were interpreted as representing a single gene fragmented during genome assembly and were therefore considered indicative of gene presence.

#### 2.3.2 Sequence variation within translated ORFs across the whole genome

To detect consistent sequence variations within the translated ORFs of the npAP population compared to the pAP population, the Proteome Comparison service in BV-BRC (Olson et al., 2023, Wattam et al., 2024) was used. This service employs the sequence comparison tools from RAST (Overbeek et al., 2014) and performs protein similarity analyses using BLASTP (Boratyn et al., 2013). Three reference genomes were used separately: the type strain *Avibacterium paragallinarum* NCTC11296 (BV-BRC Genome ID 728.107; UGHK00000000), strain ESV-135 (BV-BRC Genome ID 728.171; CP050316), and npAP/OH-USA/20210309/S1-1 (AG21-0333) (CP104914). The advanced parameters were adjusted to a minimum subject coverage of 10%, minimum subject identity of 10%, and a BLAST E-value threshold of 1e^–5^. These permissive subject-based thresholds were selected because the Proteome Comparison service calculates coverage and identity relative to the subject sequence rather than the query sequence. When pAP strains NCTC11296 or ESV-135 were used as the query, homologous proteins in npAP were expected to contain substantially longer insertions in some ORFs, as previously reported (Shelkamy et al., 2024). Consequently, these longer subject sequences could exhibit low subject coverage despite representing true homologs, potentially resulting in false-negative identifications if more stringent subject-based thresholds were applied. To avoid the inclusion of non-homologous proteins, additional filtering criteria were applied. Specifically, proteins with low subject coverage or subject identity (<50%) were considered true homologous proteins only if they were identified as bidirectional homologs by the RAST Sequence Comparison tool and met thresholds of ≥ 50% query coverage and ≥ 70% query amino acid sequence identity.

Because this service allows comparison of only nine genomes to a reference per run, multiple runs were conducted for each reference genome to include all study isolates. The outputs generated from runs using the same reference were concatenated into a single Excel file. Sequence variation analysis focused on translated ORFs that consistently exhibited less than 95% amino acid identity in one population compared to the other.

#### 2.3.3 Targeted analysis of selected virulence-associated genes

A number of virulence-associated genes or operons were identified based on either AP*/Pasteurellaceae* literature or the Virulence Factor Database (VFDB) (Liu et al., 2019) in BV-BRC version 3.35.5 (Olson et al., 2023, Wattam et al., 2024; Table 1).

**TABLE 1:** List of *Avibacterium paragallinarum* virulence-associated factors, their predicted products, and NCBI protein accession numbers, along with relevant references.

Gene/protein	Product	NCBI protein accession number	Reference	Comparative genomic outcome (npAP vs. pAP)
*HMTp210*	Trimeric autotransporter adhesin	WP_103846630.1	Yamaguchi et al. (1993) and Wang et al. (2014)	Consistently longer in npAP (2–3-fold increase compared with pAP)
*KpsS*	Capsular polysaccharide	WP_017807039.1	Sawata et al. (1985), Roberts (1996), De Smidt et al. (2004), Boyce et al. (2010), Wu et al. (2010), Tu et al. (2015), and Chen et al. (2024)	Absent in all npAP except four genomes with a defective *ccbE*; other genes are intact with no major differences observed
*KpsC*		WP_017807038.1		
*ccbE*		WP_456503313.1		
*ccbF1*		WP_017807037.1		
*ccbF2*		WP_017807036.1		
*ccbC*		WP_017807035.1		
*ccbB*		WP_051185325.1		
*hctD*		WP_017807033.1		
*hctC*		RZN77538.1		
*hctB*		WP_017807031.1		
*hctA*		WP_017807030.1		
*waaF*	Lipooligosaccharide	WP_017805194.1	Harper et al. (2003), Harper et al. (2004), Chiang et al. (2013), and Chen et al. (2024)	No major difference
*waaQ*		WP_017805166.1		No major difference
*lic1A*		AGB93813.1		See Supplementary Table 9
*lic1B*		AGB93814.1		
*lic1C*		AGB93815.1		
*lic1D*		WP_017806719.1		
*avxC*	RTX toxin	WP_017806773.1	Linhartová et al. (2010) and Küng and Frey (2013)	See Supplementary Table 9
*avxA*		WP_017806772.1		
*avxB*		AFM44712.1		
*avxD*		WP_021724213.1		
*cdtA*	Cytolethal distending toxin	WP_021724664.1	Chen et al. (2014)	No major difference
*cdtC*		WP_021724663.1		
*cdtB*		WP_017806646.1		
*flfA*	Major fimbrial subunit of F17-like fimbriae	MFQ1047997.1	Bager et al. (2013), Kudirkienė et al. (2014), and Liu et al. (2016)	See Supplementary Table 9
*hutZ*	Heme utilization system	WP_017805684.1	Negrete Abascal et al. (2009), Huo et al. (2021), and Huo et al. (2023)	See Supplementary Table 9
*hutX*		WP_017805683.1		
TonB dependent heme receptor A		WP_046097756.1		
Fur protein		WP_017805778.1		
*apaH*	Bis (5′-nucleosyl)-tetraphosphatase, symmetrical (EC 3.6.1.41) – Metallophosphatase	WP_017807070.1	Guo et al. (2025a)	No major difference
*ksgA/rsmA*	16S rRNA adenine dimethyltransferase (EC 2.1.1.182)	WP_017807071.1	Guo et al. (2025b)	No major difference
*pepN*	Metalloprotease	WP_017806547.1	González et al. (2004), Rivero-García et al. (2005), Ramón Rocha et al. (2006)	No major difference
*gor*	Glutathione reductase (EC 1.8.1.7)	WP_017807017.1	Zhi et al. (2025)	No major difference
*kdsA*	2-Keto-3-deoxy-D-manno-octulosonate-8-phosphate synthase (EC 2.5.1.55)	WP_017807394.1	VFDB (Liu et al., 2019)	No major difference
*rfaD*	ADP-L-glycero-D-manno-heptose-6-epimerase (EC 5.1.3.20)	WP_017805193.1	VFDB (Liu et al., 2019)	No major difference
*IpxA*	Acyl-[acyl-carrier-protein]–UDP-N-acetylglucosamine O-acyltransferase (EC 2.3.1.129)	WP_017807063.1	VFDB (Liu et al., 2019)	No major difference
*KatA*	Catalase KatE (EC 1.11.1.6)	WP_115248936.1	VFDB (Liu et al., 2019)	Only present in four npAP genomes
*rfaE*	D-glycero-beta-D-manno-heptose 1-phosphate adenylyltransferase (EC 2.7.7.70) / D-glycero-beta-D-manno-heptose-7-phosphate kinase (EC 2.7.1.167)	WP_017806227.1	VFDB (Liu et al., 2019)	No major difference
*gmhA/lpcA*	D-sedoheptulose 7-phosphate isomerase (EC 5.3.1.28)	WP_017806819.1	VFDB (Liu et al., 2019)	No major difference
*gmd*	GDP-mannose 4,6-dehydratase (EC 4.2.1.47)	WP_017806933.1	VFDB(Liu et al., 2019)	See Supplementary Table 9, 10
*msbB*	Lipid A biosynthesis myristoyltransferase (EC 2.3.1.243)	WP_017805788.1	VFDB (Liu et al., 2019)	No major difference
*lpxB*	Lipid-A-disaccharide synthase (EC 2.4.1.182)	WP_017807064.1	VFDB (Liu et al., 2019)	No major difference
*lpxC*	UDP-3-O-[3-hydroxymyristoyl] N-acetylglucosamine deacetylase (EC 3.5.1.108)	CDF99056.1	VFDB (Liu et al., 2019)	No major difference
*lpxD*	UDP-3-O-[3-hydroxymyristoyl] glucosamine N-acyltransferase (EC 2.3.1.191)	WP_017807061.1	VFDB (Liu et al., 2019)	No major difference
*galE*	UDP-glucose 4-epimerase (EC 5.1.3.2)	WP_017805050.1	VFDB (Liu et al., 2019)	No major difference
*gaIU*	UTP–glucose-1-phosphate uridylyltransferase (EC 2.7.7.9)	WP_017805542.1	VFDB (Liu et al., 2019)	No major difference
*wecA*	Undecaprenyl-phosphate alpha-N-acetylglucosaminyl 1-phosphate transferase (EC 2.7.8.33)	WP_017806118.1	VFDB (Liu et al., 2019)	No major difference

The translated amino acid sequences of each predicted ORF were extracted from all analyzed genomes using the BLAST service and the BLASTP search program (Boratyn et al., 2013) in BV-BRC (Olson et al., 2023). The amino acid sequences of the virulence factors from the type strain *Avibacterium paragallinarum* NCTC11296 were used as query sequences. An ORF was considered present when a significant BLASTP hit was identified with ≥ 70% query amino acid sequence identity, ≥ 50% query coverage, and an E-value ≤ 1 × 10^−5^. BV-BRC Compare Region Viewer was used to identify truncated ORFs, frameshifts, and pseudogenes in cases where short hits, with ≥ 70% amino acid sequence identity, were detected compared with the query sequence. The absence of an ORF was further confirmed by BLASTN using the same thresholds and the criteria described above for gene presence/absence determination in draft genome assemblies.

The amino acid sequences for each virulence factor were then downloaded and aligned to the corresponding virulence factor of the type strain *Avibacterium paragallinarum* NCTC11296. In case of truncated ORF, frameshifts, and pseudogenes, the corresponding nucleotide sequences were downloaded, translated in BioEdit version 7.0.5.3 (Hall, 1999), and manually inspected by sequence alignment. Multiple amino acid sequence alignments were performed using ClustalW (Thompson et al., 1994) in BioEdit version 7.0.5.3 (Hall, 1999). Additionally, MAFFT alignment (Katoh et al., 2002) within the MegAlign Pro tool of DNASTAR Navigator version 15.0.1.1 was used for complex sequences.

The active, catalytic, and metal binding sites were identified using NCBI conserved domain database (CDD) v3.21^1^ (Wang et al., 2023) and examined in the aligned amino acid sequences of npAP compared to pAP. The presence, absence, and copy number of protein domains were determined using the Pfam database (Mistry et al., 2021) within the InterPro server (Blum et al., 2024).

For the capsule genotyping, capsular polysaccharide biosynthesis loci of pAP strains 221 (GenBank FJ965541) and H18 (GenBank FJ965542) were used as reference sequences for genotypes I (Chondroitin synthesis) and II (Heparosan synthesis), respectively (Wu et al., 2010). BLASTP with ≥ 90% amino acid sequence identity, ≥ 90% query coverage, and an E-value ≤ 1 × 10^–10^ thresholds were used to determine the capsule genotype. Partial hits were manually inspected by evaluating ORF integrity and genomic context, as described in the gene presence/absence section. When a gene was fragmented across contigs but determined to be truly present, each partial hit was required to meet the ≥ 90% amino acid sequence identity threshold for genotyping.

The amino acids numbering and BV-BRC IDs (fig|genome ID.gene number) of genes/proteins in this manuscript were according to the AP type strain NCTC11296, BV-BRC ID: 728.107; NCBI accession number UGHK00000000, unless otherwise indicated. Typing this ID in the BV-BRC search bar,^2^ provides access to the nucleotide and amino acid sequences of each gene used in this study. It is worth noting that the assigned gene/protein names mentioned in Table 1, particularly those factors selected based on the literature, were based on the original literature, as these genes/proteins are often annotated under different names across different databases.

## 3 Results

### 3.1 Phylogenomic analysis

**FIGURE 1:**
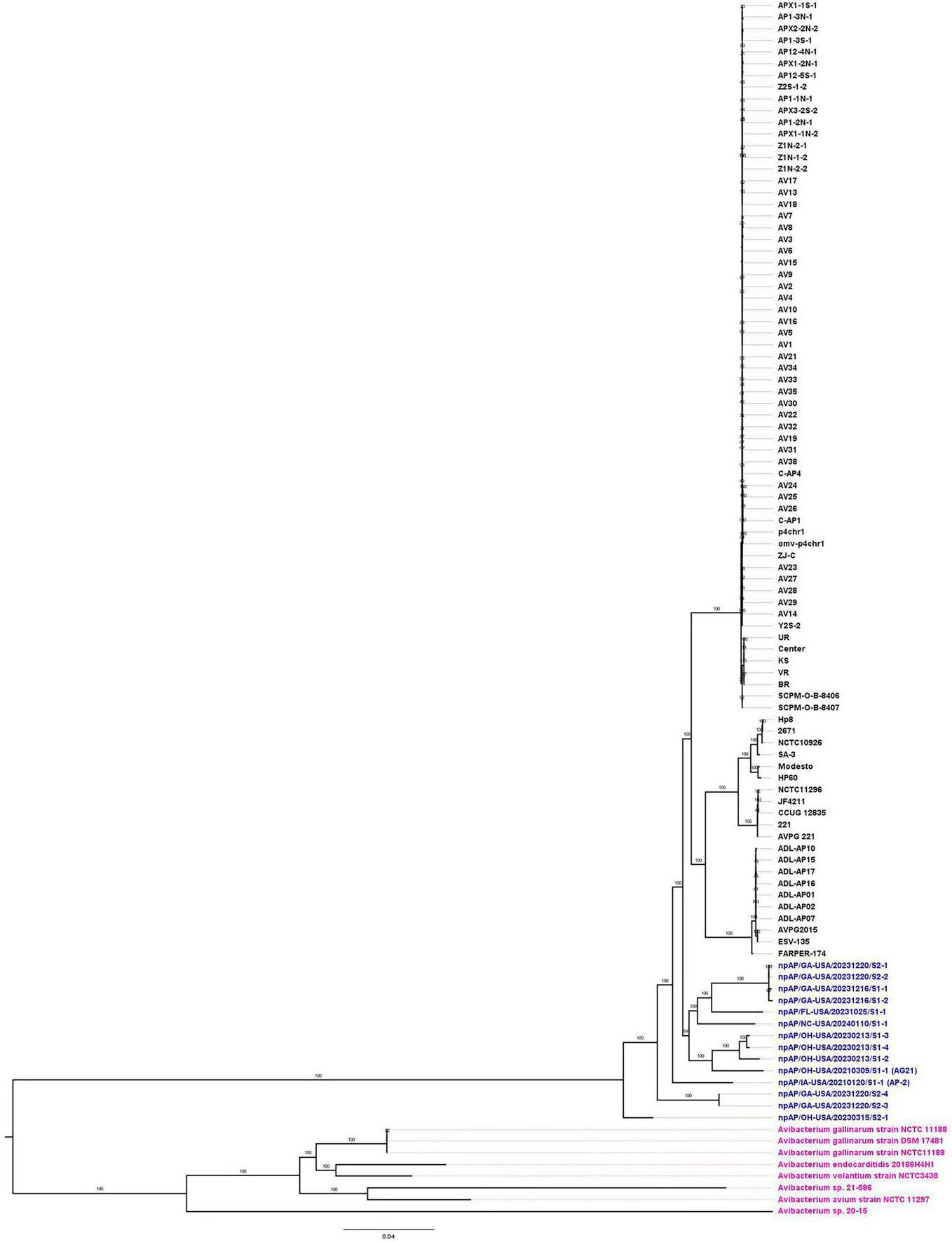
Phylogenomic tree based on 557 core genes. This phylogenomic analysis shows that the npAP genomes (blue text) cluster closely with the pAP genomes (black text) and remain clearly separated from the non-*Avibacterium paragallinarum* species (pink text). Within the npAP group, npAP/IA-USA/20210120/S1-1 (AP-2), npAP/OH-USA/20230315/S2-1, npAP/GA-USA/20231220/S2-3, and npAP/GA-USA/20231220/S2-4 form distinct clades while still maintaining close relatedness to the other npAP and pAP genomes. This tree was constructed using Bacterial Phylogenetic Tree service in BV-BRC using RAxML with 100 bootstrap replicates.

The phylogenetic tree based on 557 core genes showed that the npAP genomes clustered near the pAP genomes and were distant from non-*Avibacterium paragallinarum* species. The tree also indicated that npAP/IA-USA/20210120/S1-1 (AP-2), npAP/OH-USA/20230315/S2-1, npAP/GA-USA/20231220/S2-3, and npAP/GA-USA/20231220/S2-4 form distinct clades, while remaining more closely related to the AP genomes and distant from the other *Avibacterium* species (Figure 1). In addition to the Average Nucleotide Identity (ANI) score (Hashish et al., 2023; Shelkamy et al., 2025b), core genome phylogeny is an additional evidence that npAP are still within the AP species as npAP.

Regarding the phylogenomic clustering and the interpretation of genetic diversity, when the pAP genomes are evaluated together with their metadata (Supplementary Table 1), the clustering pattern appears to be associated primarily with geographical origin (continent level), with isolates from regions such as China and Russia clustering separately from isolates from the Americas, regardless of outbreak origin, and bacterial serovar, with some exceptions. In contrast, although all npAP isolates included in this study originated from the USA, they displayed distinct phylogenetic clustering patterns. Notably, npAP isolates originating from the same site and event did not always cluster together. For example, npAP/GA-USA/20231220/S2-1 and npAP/GA-USA/20231220/S2-2 clustered separately from npAP/GA-USA/20231220/S2-3 and npAP/GA-USA/20231220/S2-4, despite being recovered from the same site and event (Figure 1). These results highlight the greater genetic diversity observed among npAP isolates compared to pAP isolates that are highly related to each other (Figure 1).

### 3.2 Gene presence/absence

The pan-genome analysis revealed that the *hmp* and *nsrR* genes, which form an operon encoding Flavohemoglobin/Nitric oxide dioxygenase Hmp (EC 1.14.12.17) (BV-BRC ID: fig|728.107.peg.1941) and the Nitrite-sensitive transcriptional repressor NsrR (BV-BRC ID: fig|728.107.peg.1942), were exclusively present in all pAP genomes but absent in all npAP genomes. The absence of *hmp* and *nsrR* in the npAP genomes was further verified through NCBI Nucleotide BLAST analysis and confirmed by in house real-time PCR assays specifically targeting these genes (Supplementary Table 3).

The *vapD* gene, encoding the virulence-associated protein D (BV-BRC ID: fig|728.107.peg.355), was also exclusively present in all pAP genomes but absent in all npAP genomes, except for npAP/FL-USA/20231025/S1-1 (incomplete sequence) and npAP/OH-USA/20230315/S2-1. Similarly, the *ompP4* gene, encoding the outer membrane lipoprotein e (P4)/NMN 5′-nucleotidase, extracellular (EC 3.1.3.5) (BV-BRC ID: fig|728.107.peg.2185), was present in all pAP genomes but absent in all npAP genomes except for npAP/GA-USA/20231216/S1-1.

Conversely, the gene encoding CMP-N-acetylneuraminate-beta-galactosamide-alpha-2,3-sialyltransferase (EC 2.4.99.-) was uniquely absent in all pAP genomes but present in all npAP genomes, except for npAP/OH-USA/20230315/S2-1. In this isolate, the gene could not be fully identified due to the incomplete genome assembly; nevertheless, a 112-bp sequence at the 5′ end of the gene of npAP/OH-USA/20210309/S1-1 (AG21-0333) aligns to the start of contig 2, suggesting that the gene is likely present but appears absent because of gaps between contigs. Two additional npAP genomes, npAP/IA-USA/20210120/S1-1 (AP-2) and npAP/GA-USA/20231220/S2-4, harbor nucleotide deletions at positions 475/476 in AP-2 and at position 28 in npAP/GA-USA/20231220/S2-4, respectively, when aligned to the corresponding gene sequence of AG21-0333 (BV-BRC ID: fig|728.352.peg.1996). These deletions result in frameshifts and the introduction of premature stop codons.

### 3.3 Sequence variation within translated ORFs across the whole genome

**FIGURE 2:**
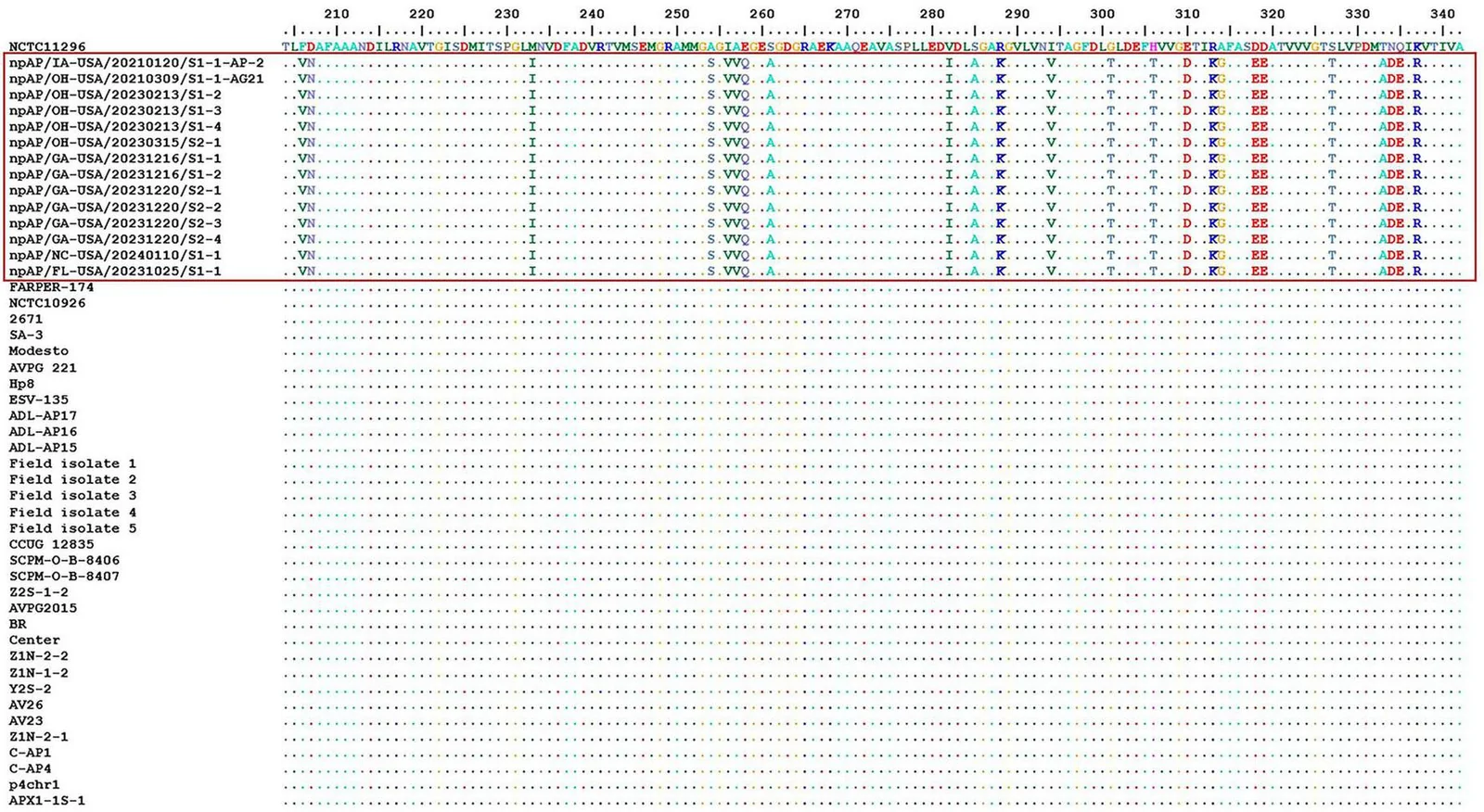
Multiple amino acid sequence alignment of *Avibacterium paragallinarum* FtsZ protein sequences. The alignment highlights consistent amino acid substitutions in all npAP isolates (red box) within the region spanning positions 206–337, compared to the AP type strain NCTC11296 and representative pAP strains. Identical amino acid positions are shown as dots. The alignment was generated using ClustalW in BioEdit version 7.0.5.3.

Sequence variation analysis of the ORFs across all genomes identified only a few genes exhibiting sequence divergence between npAP and pAP. Among these, the *ftsZ* gene, encoding the cell division protein FtsZ (BV-BRC ID: fig|728.107.peg.988), was the only gene that consistently exhibited sequence divergence in all npAP genomes compared to all pAP genomes. FtsZ showed 98.6–100% amino acid identity among npAP genomes and 92.2–94.5% identity when compared to pAP genomes (Figure 2). FtsZ contains two conserved protein domains: the Tubulin/FtsZ family GTPase domain (IPR003008) and the FtsZ family C-terminal domain (IPR024757), and the observed amino acid substitutions were localized specifically within the C-terminal domain. The results of the entire proteome analysis compared to the type strain are presented in Supplementary Table 4, whereas the partially consistent divergence observed in some ORFs is presented in Supplementary Table 5.

### 3.4 Targeted analysis of selected virulence-associated genes

#### 3.4.1 Identified from AP/*Pasteurellaceae* literature

##### 3.4.1.1 HMTp210

The HMTp210 adhesin (BV-BRC ID: fig|728.107.peg.1638) has been shown to be one of the major contributors to AP virulence (Yamaguchi et al., 1993, Wang et al., 2014). Our analysis revealed that the HMTp210 of the npAP was 2 to 3 times longer than its homolog in the pAP. The translated ORFs range from 4291 to 6681 amino acids in npAP, compared to 2034 to 2084 amino acids in the pAP.

**FIGURE 3:**
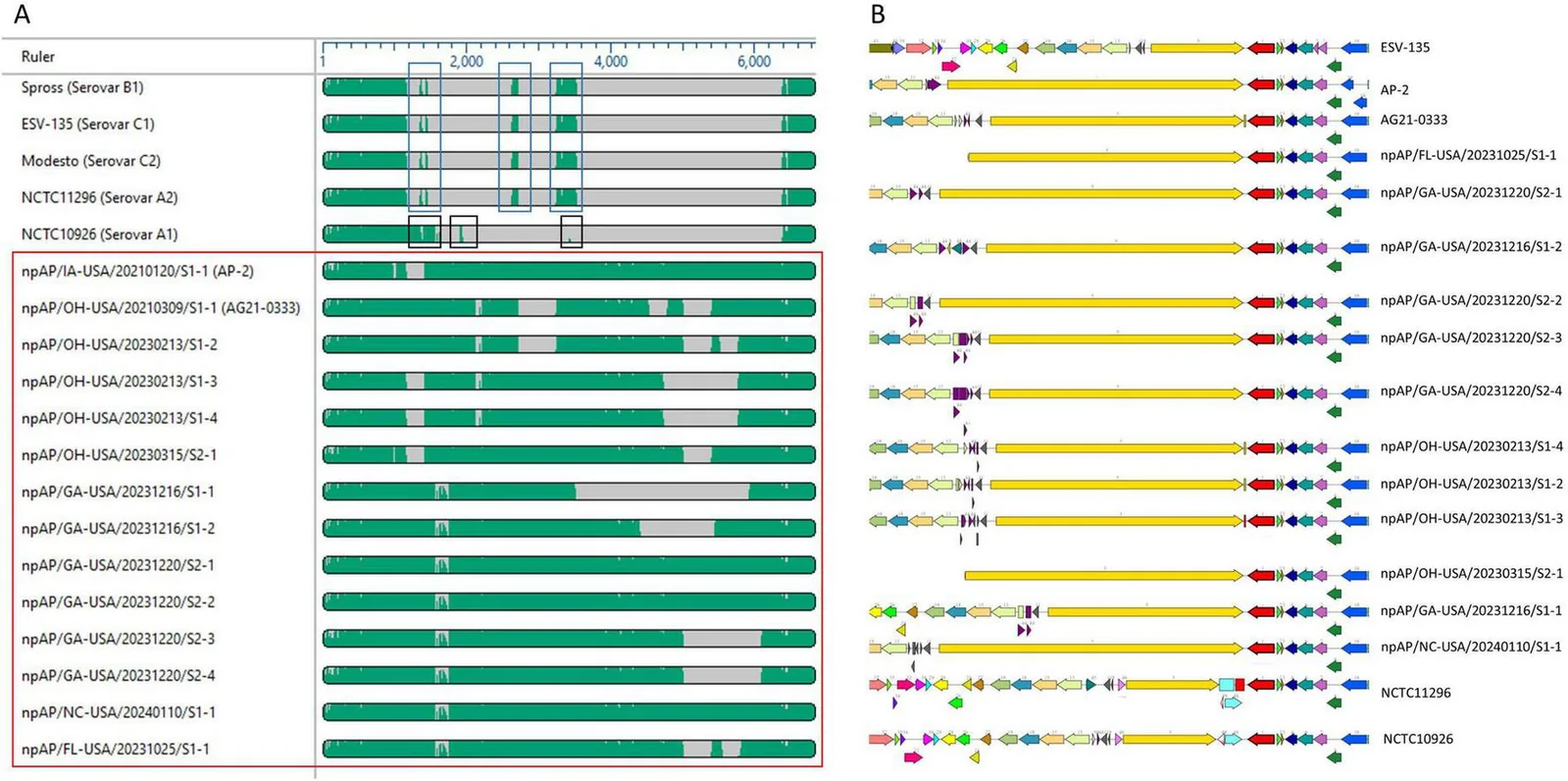
Length variation in the HMTp210 between pAP and npAP. The figure illustrates the substantial length increase in the HMTp210 of npAP isolates, which are 2–3 times longer than that of pAP. **(A)** Multiple amino acid sequence alignment generated using MAFFT in MegAlign Pro (DNASTAR Navigator v15.0.1.1). Five representative pAP HMTp210 proteins are shown in comparison with the npAP set (highlighted by the red box). Blue boxes indicate the HVRs of the known pAP serovars (B1, C1, C2, and A2), except for serovar A1, which is marked by black boxes. Green regions represent aligned sequence, whereas gray regions indicate gaps (absence of sequence), corresponding to the large insertions present in the npAP HMTp210. **(B)** Genomic context, location, and orientation of the HMTp210 gene (yellow arrow) visualized using the BV-BRC Compare Region Viewer. The figure shows that the HMTp210 gene is consistently much longer in all npAP isolates compared to the pathogenic strains (ESV-135, NCTC11296, and NCTC10926).

The difference in length resulted from large nucleotide insertions between regions I and III of the *HMTp210* gene, disrupting the hypervariable region (HVR) (Figure 3). BLASTP analysis of the translated amino acid sequences from four major insertions showed similarity to YadA-like adhesins from multiple species, including *Gallibacterium anatis*, *Gallibacterium trehalosifermentans*, *Pasteurella* sp. *PK-2025*, *Fusobacterium* sp., *Histophilus somni*, *Veillonella magna*, and *Mergibacter septicus*. BLASTP also identified similarity hits within AP species (Supplementary Table 6). Notably, one insertion showed similarity to a YadA-like adhesin distinct from HMTp210 in strain ESV-135 (fig|728.171.peg.1142) and in other AP strains such as AV26, AV25, AV24, and npAP npAP/IA-USA/20210120/S1-1 (AP-2). Another insertion showed similarity to a third YadA-like adhesin in ESV-135 (fig|728.171.peg.1273) and in many pAP and npAP strains. We refer to fig|728.171.peg.1142 and its homologs as Adhesin 2, and fig|728.171.peg.1273 and its homologs as Adhesin 3. Additionally, one insertion showed similarity to the HMTp210 of the pAP strain ENV-1065 (Supplementary Table 6).

Multiple amino acid sequence alignments of the five reference pAP strains and the study’s npAP set revealed that the npAP HMTp210 contain more than one hypervariable region (HVR). One HVR showed similarity to the serovar A1 HVR, while the other resembled HVRs from the remaining serovars (Figure 3). The A1-like HVR remained largely intact after region I in all npAP isolates except npAP/IA-USA/20210120/S1-1(AP-2), npAP/OH-USA/20230213/S1-3, npAP/OH-USA/20230213/S1-4, and npAP/OH-USA/20230315/S2-1. In these four isolates, A1 HVR similarity begins only around amino acid position 1,357 (based on NCTC10296 numbering), representing the second half of the HVR, and is interrupted by multiple small insertions. In contrast, the remaining npAP isolates retain an A1-like HVR that is nearly intact up to approximately amino acid position 1,529. However, all of these isolates share a 21-amino-acid insertion at position 1,337/1,338 that disrupts the HVR, and the final ∼46 amino acids are additionally interrupted by two large insertions. The second HVR, which corresponded to the rest of serovars, was also disrupted in the npAP sequences by three to four insertions as compared to serovars (B1, C1, C2, and A2) (Figure 3). A similar pattern was observed for serovars C3, C4, A3, and A4, with minor exceptions (Supplementary Figure 1).

**FIGURE 4:**
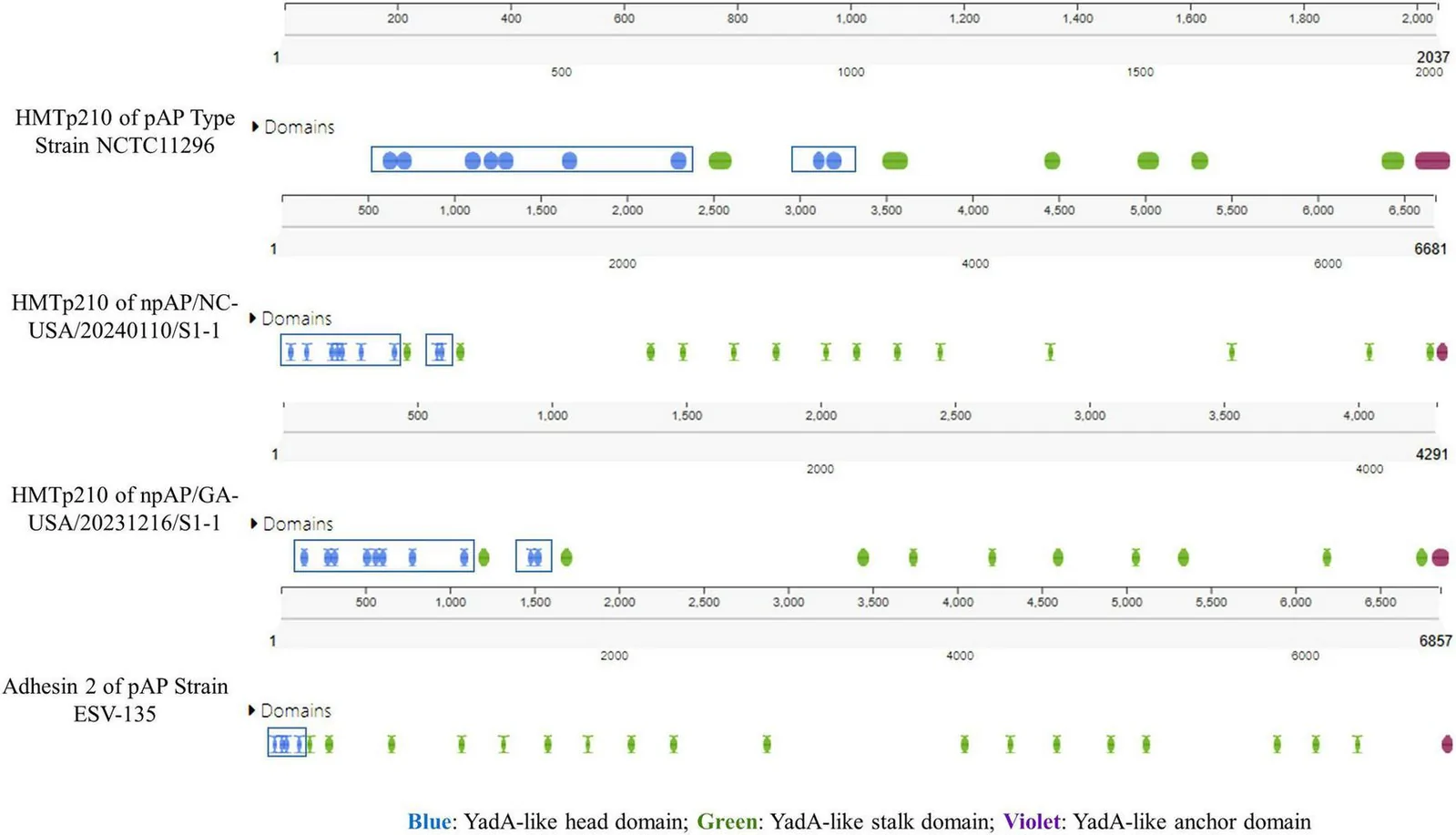
Domain architecture of HMTp210 and Adhesin 2 proteins. The figure shows the domain architecture of HMTp210 in pAP and npAP strains, highlighting similarities in the YadA-like head domains (blue boxes) and clear differences in the stalk regions (green). The HMTp210 of the type strain NCTC11296 is shown as the representative pAP sequence. For npAP, the longest homolog (6681 amino acids, npAP/NC-USA/20240110/S1-1) and the shortest homolog (4291 amino acids, npAP/GA-USA/20231216/S1-1) are displayed. The domain architecture of Adhesin 2, a long YadA-like adhesin present in all npAP strains and most pAP strains, is also shown and exhibits a length comparable to npAP HMTp210.

Protein domain analysis of HMTp210 identified YadA-like head (IPR008640), stalk (IPR008635), and anchor (IPR005594) domains in both pAP and npAP. The number and positions of YadA-like head domains were broadly similar between pAP and npAP; these head domains are located within the N-terminus ∼1,000 amino acids of HMTp210 and appear as two heads of differing length (one long, one short) in both groups (Figure 4). By contrast, npAP genomes contained a greater number of YadA-like stalk domains than pAP (Figure 4 and Table 2). A single YadA-like anchor domain was detected in both pAP and npAP.

**TABLE 2:** Comparison of HMTp210 proteins between five reference pAP strains and the study’s npAP isolates, including amino acid length and numbers of YadA-like domains.

Isolate^a^	Pathogenicity	HMTp210 length (amino acids)	Number of YadA-like head domains^b^	Number of YadA-like stalk domains	Number of YadA-like anchor domains
NCTC11296	Pathogenic	2,037	11	6	1
ESV-135	Pathogenic	2,038	12	6	1
NCTC10926 (0083)	Pathogenic	2,042	11	4	1
Modesto	Pathogenic	2,038	11	6	1
Spross	Pathogenic	2,037	12	6	1
npAP/IA-USA/20210120/S1-1 (AP-2)	Nonpathogenic	6,494	10	15	1
npAP/OH-USA/20210309/S1-1 (AG21)	Nonpathogenic	5,547	12	12	1
npAP/OH-USA/20230213/S1-2	Nonpathogenic	5,547	12	12	1
npAP/OH-USA/20230213/S1-3	Nonpathogenic	5,432	12	14	1
npAP/OH-USA/20230213/S1-4	Nonpathogenic	5,432	12	14	1
npAP/OH-USA/20230315/S2-1	Nonpathogenic	6,108	10	15	1
npAP/GA-USA/20231216/S1-1	Nonpathogenic	4,291	13	10	1
npAP/GA-USA/20231216/S1-2	Nonpathogenic	5,649	13	13	1
npAP/GA-USA/20231220/S2-1	Nonpathogenic	6,674	12	14	1
npAP/GA-USA/20231220/S2-2	Nonpathogenic	6,674	12	14	1
npAP/GA-USA/20231220/S2-3	Nonpathogenic	5,574	12	13	1
npAP/GA-USA/20231220/S2-4	Nonpathogenic	5,574	12	13	1
npAP/NC-USA/20240110/S1-1	Nonpathogenic	6,681	11	14	1
npAP/FL-USA/20231025/S1-1	Nonpathogenic	6,033	11	13	1

^a^The *HMTp210* gene sequences were extracted from the study genomes, except for the Spross strain, the MW533118 GenBank accession was used (Tan et al., 2021).

^b^The number of YadA-like head domains varied slightly among pAP strains (11–12 domains) and npAP strains (10–13 domains); however, the overlapping ranges indicate that head domain numbers were broadly similar between the two populations. Naming format for nonpathogenic *Avibacterium paragallinarum* isolates: npAP/location/YearMonthDay/site-isolate number.

**FIGURE 5:**
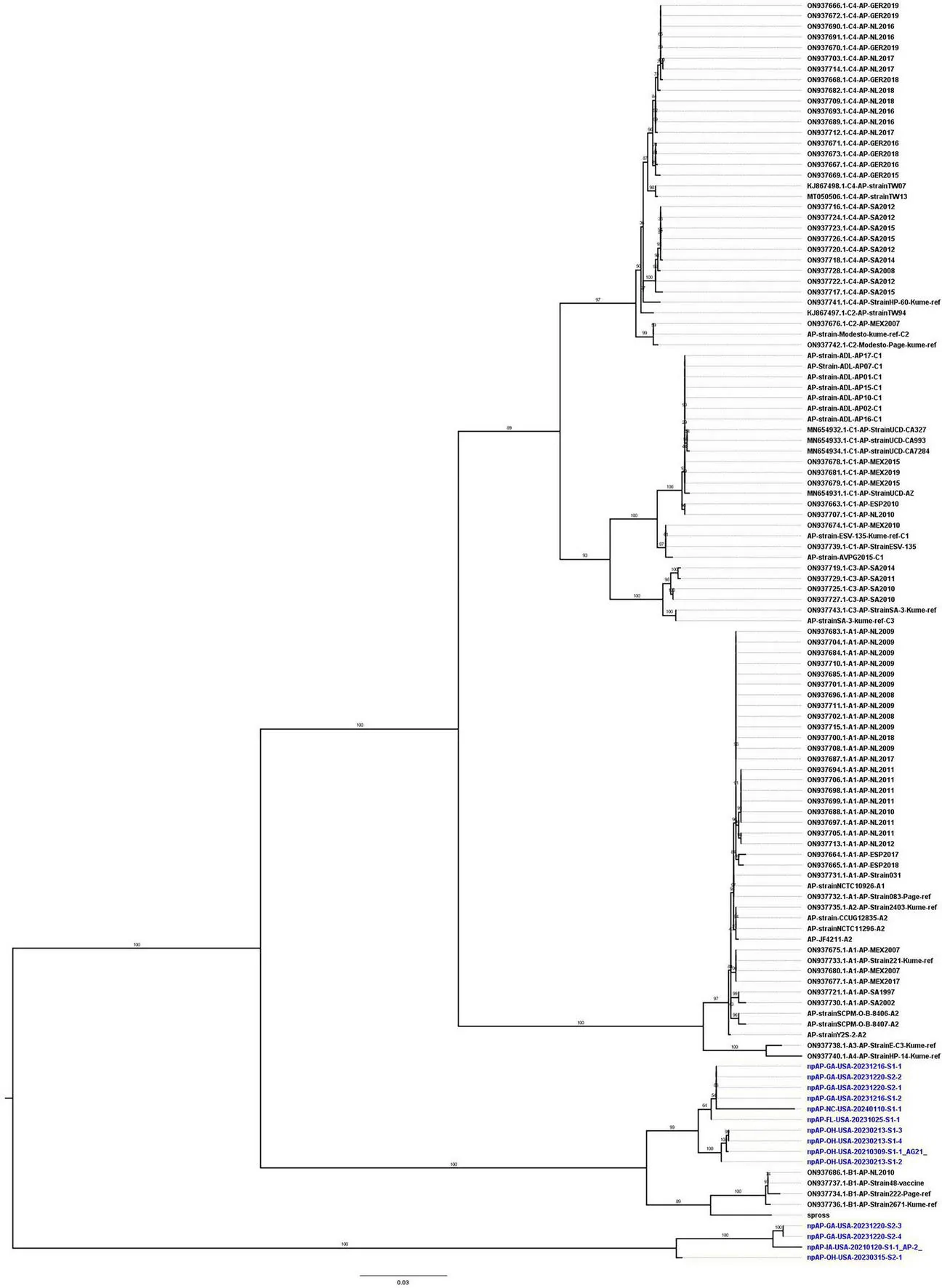
Phylogenetic tree based on the first 1,200 bp of the *HMTp210* gene. Black taxa beginning with accession numbers represent pAP sequences obtained from Buter et al. (2023), while those without accession numbers were extracted from the WGS data analyzed in this study. For the Spross strain, the MW533118 GenBank accession was used (Tan et al., 2021). The phylogenetic analysis shows that the npAP isolates (blue text) cluster into two major groups: one group positioned close to serogroup B, and a second, distinct cluster composed of npAP/IA-USA/20210120/S1-1 (AP-2), npAP/OH-USA/20230315/S2-1, npAP/GA-USA/20231220/S2-3, and npAP/GA-USA/20231220/S2-4. The tree was constructed using IQ-TREE multicore version 1.6.12, and the best-fit model (HKY+F+G4) was selected, with ultrafast bootstrap performed using 1,000 replicates.

The phylogenetic analysis of the first 1,200 bp of *HMTp210* showed that the npAP isolates clustered into two major groups: one clustered close to serogroup B of pAP, and the other forming a distinct, unique cluster similar to the core genome phylogeny comprising npAP/IA-USA/20210120/S1-1 (AP-2), npAP/OH-USA/20230315/S2-1, npAP/GA-USA/20231220/S2-3, and npAP/GA-USA/20231220/S2-4 (Figure 5).

The analysis revealed that HMTp210 is not the only YadA-like adhesin in AP species. Adhesin 2 and Adhesin 3 are also YadA-like adhesins, with lengths of 6,857 and 3,521 amino acids, respectively, in the ESV-135 strain. Homologs of both Adhesin 2 and Adhesin 3 were present in all npAP isolates with comparable lengths and amino acids identities of 94.7–99% and 87.7–94.9%, respectively, compared to ESV-135 strain. Adhesin 2 homologs were also detected in most pAP genomes, except NCTC11296, HP60, HP8, AVPG221, SA-3, Modesto, 2671, NCTC10926, CCUG12835, 221, and JF4211. Adhesin 3 was present in all pAP genomes. Length variability was observed for both Adhesin 2 and Adhesin 3 across AP genomes. Adhesin 2 drew particular attention because its size was comparable to that of the npAP HMTp210. At the amino acid sequence level, Adhesin 2 was distinct from npAP HMTp210; however, the number and distribution of protein domains were broadly comparable, except for the head region. npAP HMTp210 appears to possess two heads, whereas Adhesin 2 contains a single head followed by a long stalk and an anchor domain (Figure 4).

##### 3.4.1.2 Capsular polysaccharide

The capsular polysaccharide was investigated as one of the major virulence factors of AP (Sawata et al., 1985; Tu et al., 2015; Chen et al., 2024). Genetically, the capsular polysaccharide locus comprises three main regions. The first region consists of four genes (*hctA*, *hctB*, *hctC*, and *hctD*) that encode the secretion system responsible for exporting capsular polysaccharides to the bacterial surface (De Smidt et al., 2004). The second region is the biosynthesis module, which includes 5–6 genes involved in polymerization of genotype-specific capsules (Wu et al., 2010). Two genotypes (I and II) for AP have been described (Wu et al., 2010), encoded by *acbEDGHCB* and *ccbEF1F2CB*, respectively. The third region contains two genes encoding proteins responsible for lipidation and anchorage of the capsular polysaccharide (Roberts, 1996; Boyce et al., 2010). The capsular polysaccharide biosynthesis loci of pAP strains 221 (GenBank FJ965541) and H18 (GenBank FJ965542) were used as reference sequences for genotypes I (Chondroitin synthesis) and II (Heparosan synthesis), respectively (Wu et al., 2010).

In this study, the capsular polysaccharide locus was present in all pAP genomes and absent in all npAP genomes except four: npAP/GA-USA/20231216/S1-1, npAP/GA-USA/20231216/S1-2, npAP/GA-USA/20231220/S2-1, and npAP/GA-USA/20231220/S2-2. Based on amino acid similarity of biosynthesis genes relative to strains 221 (Genotype I) and H18 (Genotype II), the capsule of the four npAP genomes carrying the locus was classified as Genotype II, with high similarity to the type strain NCTC11296 across the locus (Supplementary Table 7).

**FIGURE 6:**
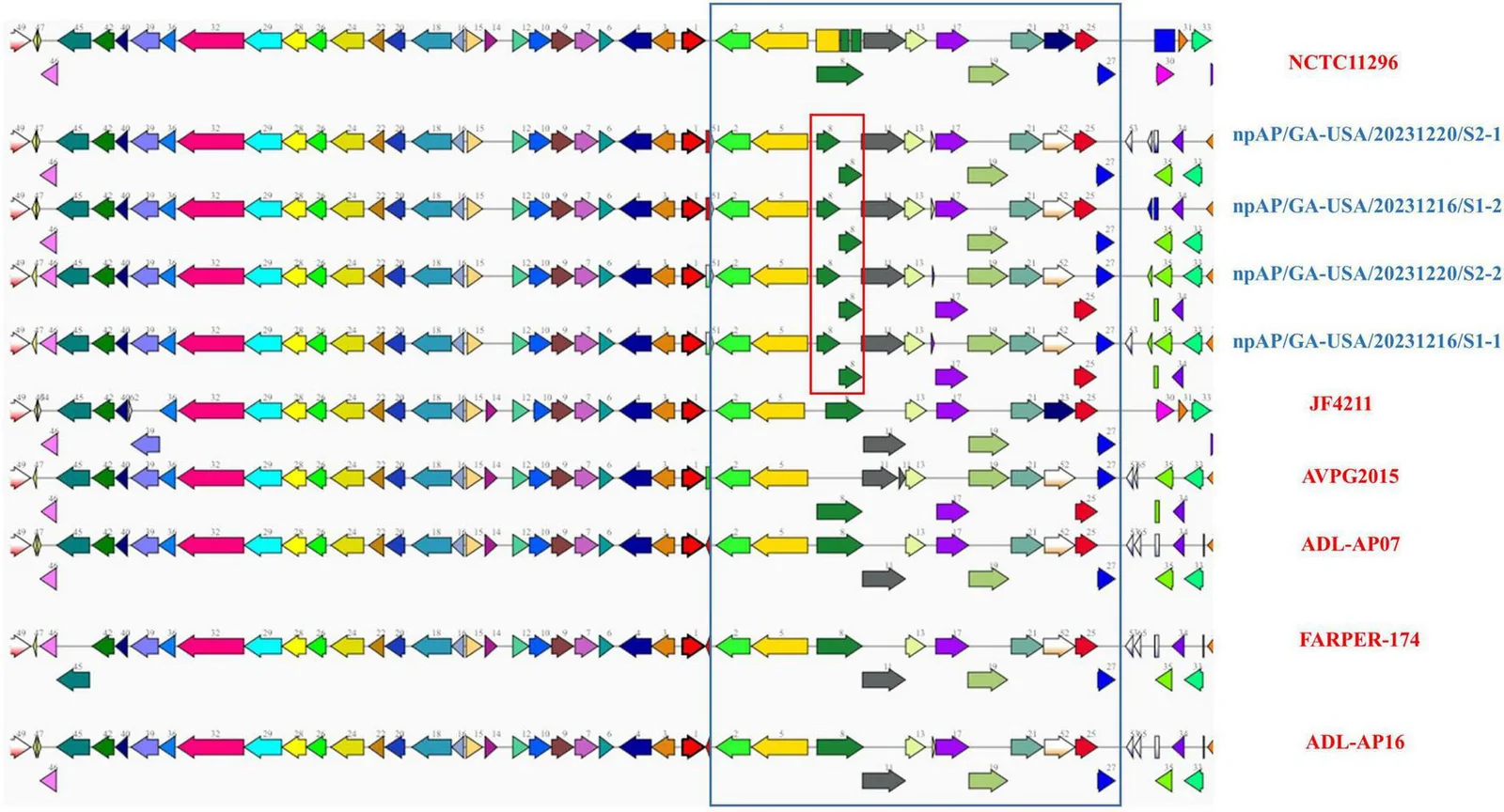
Capsular polysaccharide locus of npAP and pAP. The figure shows the genomic context, location, and orientation of the capsular polysaccharide locus (blue box) visualized using the BV-BRC Compare Region Viewer. Representative pAP strains belonging to capsule genotype II (shown in red text) are included alongside npAP genomes that contain the capsular polysaccharide locus (shown in blue text). The red box highlights the ccbE ORF, which is split in npAP genomes compared to the intact ORF observed in pAP strains.

The four npAP genomes showed an intact capsular polysaccharide locus, including the *hctA* gene, with exceptional features observed. First, the *ccbE* ORF was split into two fragments (Figure 6) due to a 35-nucleotide deletion (CTTACCTTACCTTACCTTACCTTACCTTACCTTAC) at position 832, resulting in a frameshift and introduction of a premature stop codon. Two additional deletions were also identified at positions 1,095 and 1,475. The 35-nucleotide deletion was confirmed by PCR and Sanger sequencing (Supplementary Table 8). Second, an additional gene encoding a hypothetical protein of 33 amino acids was detected within the biosynthesis locus. A unique feature was observed in the *KpsS* gene of npAP/GA-USA/20231216/S1-2, which is truncated due to an adenine deletion at nucleotide position 46. This deletion causes a frameshift, resulting in a new downstream start codon beginning at position 49.

Similarly, some genetic defects were observed in a few pAP genomes. Isolate ESV-135 (CP050316), which was sequenced using the PacBio Sequel platform, assembled with Canu v1.5, and had an average genome coverage of 36 ×, contained deletions in *ccbE* and indels in *ccbF1* and *ccbF2*, resulting in frameshifts and premature stop codons. Strain 221 (AOGF00000000), which was sequenced using Illumina HiSeq with an average genome coverage of 100 × and assembled using SOAPdenovo v 1.05, harbored an insertion of CTTACCTTAC in *ccbE* at nucleotide position 866/867, introducing multiple stop codons and disrupting the open reading frame.

A cryptic *ccbD* homolog (BV-BRC ID: fig|728.107.peg.2807) located outside the capsule locus was identified in NCTC11296 but absent in npAP isolates. The capsule of the type strain NCTC11296 was classified as Genotype II, and the BV-BRC IDs and protein domains of all capsule locus-associated genes are presented in Table 3.

**TABLE 3:** Capsular polysaccharide locus of the AP type strain NCTC11296 and associated protein domains encoded by each gene.

Gene	BV-BRC ID^a^	Function	Protein domain^b^*
*KpsS*	fig|728.107.peg.1741	Lipidation and anchorage	IPR007833
*KpsC*	fig|728.107.peg.1742		IPR007833
*ccbE*	fig|728.107.peg.1743	Biosynthesis	Not defined
*ccbF1*	fig|728.107.peg.1744		IPR001173
*ccbF2*	fig|728.107.peg.1745		Not defined
*ccbC*	fig|728.107.peg.1746		IPR001732; IPR014026; IPR014027
*ccbB*	fig|728.107.peg.1747		IPR001173
*hctD*	fig|728.107.peg.1748	Export	IPR003715; IPR054765
*hctC*	fig|728.107.peg.1749		Not defined
*hctB*	fig|728.107.peg.1750		IPR013525
*hctA*	fig|728.107.peg.1751		IPR003439

^a^The BV-BRC ID of AP type strain NCTC11296 was used for all genes, of the capsular polysaccharide locus. Typing this ID in the BV-BRC search bar https://www.bv-brc.org/, provides access to the nucleotide and amino acid sequences of each gene.

^b^The integrated accession number for the representative protein domain was obtained from the InterPro server.

*The same domains were present in the four npAP genomes: npAP/GA-USA/20231216/S1-1, npAP/GA-USA/20231216/S1-2, npAP/GA-USA/20231220/S2-1, and npAP/GA-USA/202312 20/S2-2, similar to pAP.

Transcriptional regulators of capsule expression were also investigated. A homolog of the Fis protein was identified in the type strain (BV-BRC ID: fig|728.107.peg.2923), which modulates capsular transcription (Steen et al., 2010), and shared 94.95% identity with the Fis protein of *Pasteurella multocida* strain Pm70 (UniProt P57902). Similarly, a homolog of Hfq was identified in the AP type strain NCTC11296 (BV-BRC ID: fig|728.107.peg.1415), a regulator of capsular translation (Mégroz et al., 2016), and showed 97.18% identity to *Pasteurella multocida* strain Pm70 Hfq (UniProt Q9CMC6). Both Fis and Hfq were present in the four npAP genomes carrying the capsule locus and were 100% identical to those found in the majority of pAP genomes.

The rest of the virulence factors identified from the literature and mentioned in Table 1 were investigated in the same way; however, we did not find major differences between npAP and pAP. Nevertheless, there are some generally significant findings and insights that can benefit the AP research community. The results of these findings are available in Supplementary Table 9.

#### 3.4.2 Identified from VFDB

No major differences were observed in npAP compared to pAP, except for the *KatA* gene, which encodes catalase KatE (EC 1.11.1.6). Biochemically, AP is distinguished from the rest of *Avibacterium* species by being catalase-negative (Blackall et al., 2005; Bisgaard et al., 2007), however, in this study, the *KatA* gene was detected in four npAP isolates: npAP/OH-USA/20210309/S1-1 (AG21-0333), npAP/OH-USA/20230213/S1-2, npAP/OH-USA/20230213/S1-3, and npAP/OH-USA/20230213/S1-4, while it was absent in the remaining AP genomes. The BV-BRC ID of *KatA* in the AG21-0333 isolate is fig|728.352.peg.1751. The analysis of the remaining factors is presented in Supplementary Tables 9, 10.

## 4 Discussion

AP is a primary bacterial pathogen within genus *Avibacterium* of family *Pasteurellaceae*, and it is the causative agent of IC in chickens (Blackall et al., 1997; Blackall et al., 2005). Among poultry pathogens within the family *Pasteurellaceae*, *Pasteurella multocida*, the etiologic agent of fowl cholera, has long served as a well-studied model organism. Although several virulence factors of AP have been examined, the overall understanding of AP virulence factors and pathogenesis remain incomplete and not fully integrated. More in-depth and coordinated investigations are therefore needed to clarify the roles of these factors and to uncover additional contributors to AP pathogenicity.

The isolation and acquisition of complete and draft genome sequences of npAP isolates (Hashish et al., 2023; Shelkamy et al., 2025a,b), provide a unique opportunity to study AP pathogenicity and its virulence factors. This natural contrast between npAP and pAP isolates offers a straightforward and biologically relevant comparative framework for identifying the major virulence factors that drive AP pathogenicity and for assessing how defects or alterations in these determinants contribute to the naturally occurring nonpathogenic phenotype. Therefore, the main objective of this article was to define the major virulence factors associated with AP pathogenicity through comparative genomics between npAP and pAP. As a result, this manuscript can serve as a review with a list of all known AP-associated virulence factors consolidated into a single comprehensive reference for researchers investigating AP pathogenesis.

The current study demonstrated that npAP isolates indeed belong to the AP species and are clearly distinct from other *Avibacterium* species based on phylogenomic analyses, consistent with previously reported ANI score interpretations for these isolates (Hashish et al., 2023, Shelkamy et al., 2025b). Phylogenomic analysis further indicated that the pAP population is relatively uniform, whereas the npAP population exhibits greater genetic diversity. Moreover, the data suggest the possibility that pAP may have originated from a single evolutionary event; however, the directionality of evolution between pAP and npAP remains to be elucidated.

The current study also uncovered four major and consistent genetic features that distinguish all known npAP isolates from all pAP genomes. First, the *HMTp210* gene, is 2–3 times longer in npAP compared to pAP. The second consistent genomic feature is that all npAP isolates exhibit either complete absence of the capsular polysaccharide locus or defects in specific genes within the locus. Third, npAP lack both *hmp* and *nsrR*, which encode the nitric oxide detoxification system. Fourth, the *ftsZ* gene, which encodes the essential cell-division protein FtsZ, exhibits substantial allelic divergence between npAP and pAP. In addition, the majority of npAP isolates encode a putative sialyltransferase and lack OmpP4, whereas all pAP genomes lack the putative sialyltransferase and encode OmpP4 (Table 4)

**TABLE 4:** Summary of the main genetic features distinguishing the npAP population from the pAP population.

npAP genomes	HMTp210^a^	Capsular polysaccharide locus			Nitric oxide dioxygenase system	FtsZ^c^	Putative sialyltransferase encoding gene^d^	*ompP4*
		Presence/ absence	*hctA*	*ccbE* ^b^				
npAP/IA-USA/20210120/S1-1 (AP-2)	71%	Absent	Absent	Absent	Absent	94.5%	Disrupted	Absent
npAP/OH-USA/20210309/S1-1 (AG21-0333)	80.7%	Absent	Absent	Absent	Absent	93.1%	Present	Absent
npAP/OH-USA/20230213/S1-2	80.8%	Absent	Absent	Absent	Absent	93.1%	Present	Absent
npAP/OH-USA/20230213/S1-3	75.9%	Absent	Absent	Absent	Absent	93.1%	Present	Absent
npAP/OH-USA/20230213/S1-4	76%	Absent	Absent	Absent	Absent	93.1%	Present	Absent
npAP/GA-USA/20231216/S1-1	81.4%	Present	Present	Disrupted	Absent	93.3%	Present	Present
npAP/GA-USA/20231216/S1-2	81.4%	Present	Present	Disrupted	Absent	93.3%	Present	Absent
npAP/GA-USA/20231220/S2-1	81.4%	Present	Present	Disrupted	Absent	93.3%	Present	Absent
npAP/GA-USA/20231220/S2-3	78.9%	Absent	Absent	Absent	Absent	93.1%	Present	Absent
npAP/NC-USA/20240110/S1-1	79.9%	Absent	Absent	Absent	Absent	92.9%	Present	Absent
npAP/GA-USA/20231220/S2-2	81.4%	Present	Present	Disrupted	Absent	93.3%	Present	Absent
npAP/GA-USA/20231220/S2-4	78.9%	Absent	Absent	Absent	Absent	93.1%	Disrupted	Absent
npAP/OH-USA/20230315/S2-1	73.2%	Absent	Absent	Absent	Absent	92.9%	Incomplete sequence	Absent
npAP/FL-USA/20231025/S1-1	82%	Absent	Absent	Absent	Absent	92.2%	Present	Absent

Naming format for nonpathogenic *Avibacterium paragallinarum* isolates: npAP/location/YearMonthDay/site-isolate number.

*^a^*HMTp210 amino acid identity (%) relative to the homologous protein of the type strain NCTC11296 (WP_103846630.1).

*^b^*Distrupted refers to frameshifts and introduction of premature stop codons.

*^c^*npAP FtsZ amino acid identity (%) relative to the homologous protein of the type strain NCTC11296 (WP_017805978.1), For comparison, the FtsZ amino acid identity of the pAP genomes relative to the type strain ranged from 99.3 to 100%.

*^d^*Incomplete sequence refers to partial sequence hits located at the ends of contigs; therefore, gene presence or absence could not be determined due to the draft genome assembly. Present refers to an intact, complete ORF; absent refers to the complete absence of an ORF.

HMTp210 is a trimeric autotransporter adhesin (TAA) that mediates haemagglutination, cell adherence, and biofilm formation (Wang et al., 2014). Its importance as a major virulence factor in AP was previously demonstrated (Yamaguchi et al., 1993; Wang et al., 2014). HMTp210 is also the major protective antigen that confers immunity against AP infection (Iritani et al., 1980; Noro et al., 2008; Wu et al., 2011; Sakamoto et al., 2013; Chen et al., 2024).

HMTp210 is a YadA-like TAA composed of a head, stalk, and anchor domains (Li et al., 2023). The head domain is responsible for adhesion to extracellular matrix proteins (El Tahir and Skurnik, 2001; Linke et al., 2006), the stalk plays a critical role in projecting the head outward from the bacterial membrane to reach host cell surfaces (Linke et al., 2006; Shahid, 2012) and contributes to serum resistance against complement-mediated killing (Roggenkamp et al., 2003), while the anchor forms a membrane pore that enables autotransport (Grosskinsky et al., 2007). Our analysis showed that npAP HMTp210 retains nearly identical head and anchor domains compared with pAP, but contains substantially more stalk domains, resulting in a protein that is two to three times longer in npAP. We hypothesize that a longer stalk may impair efficient head projection, may reduce structural rigidity needed for proper head positioning, cause tilting or bending that limits adhesion, or diminish serum resistance, potential explanations for the nonpathogenic phenotype of npAP, however further experimental validation is needed to test this hypothesis.

A TAA as large as the npAP HMTp210 (4,291 to 6,681 amino acids) has not previously been reported in the literature. The largest known functional TAA is BadA of *Bartonella henselae*, approximately 3,100 amino acids, described by Riess et al. (2004) and confirmed to be a major pathogenicity factor. Another example is AtaA from the nonpathogenic *Acinetobacter* sp. strain Tol 5 (3,630 amino acids) (Yoshimoto et al., 2016) which exhibits nonspecific adhesiveness to both abiotic and biotic surfaces. Yoshimoto et al. (2016) attributed this nonspecific adhesion to its 3D fibrous structure rather than its large size alone. This explanation may also apply to npAP HMTp210, either functioning as a nonspecific adhesin that becomes diverted by inappropriate surfaces, thereby attenuating virulence, or becoming misfolded and thus nonfunctional. Even more intriguingly, our study identified additional unusually large TAAs (Adhesin 2 and Adhesin 3) in both pAP and npAP, a finding that, to our knowledge, has never been reported in AP before, and their roles remain unknown and require further investigation.

The HVR is part of the stalk region, and it has been demonstrated that the HVR is highly antigenic and a potent protective protein against AP infection (Wu et al., 2011; Sakamoto et al., 2013). However, its role in bacterial pathogenesis remains unclear. Because the HVR lies within the stalk, it may contribute to head protrusion or serum resistance, especially since it has been shown that not only the head but also the stalk are surface-exposed (Roggenkamp et al., 2003; Linke et al., 2006). Two main features of the HVR were noted in the npAP isolates. First, the HVR is not intact; instead, it is disrupted by multiple sequence insertions. We speculate that these insertions render the HVR nonfunctional, which could negatively affect bacterial pathogenicity. Further studies are needed to determine the functional consequences of this disruption and how it relates to AP virulence. Second, the npAP HMTp210 contains two HVRs at the amino acids level: one resembling serovar A1, which clusters distantly from serovars B1, C1-C4, and A2-A4 (Buter et al., 2023), and another resembling the HVRs of the rest of serovars. In addition to phylogenomic analysis, and the presence of additional large TAAs in both pAP, and npAP, this HVR finding raises an important evolutionary question that requires further investigation: which form arose first, npAP or pAP? At the level of HMTp210, npAP could have evolved into pAP through sequence loss, or pAP could have evolved into npAP through sequence gain.

Additionally, it is worth noting that the amino acid sequences of the npAP HMTp210 insertions showed BLASTP similarity hits with YadA-like adhesins from other bacterial species, particularly *Gallibacterium anatis*. This finding raises the possibility that horizontal gene transfer (HGT) may have contributed to shaping the unique HMTp210 architecture in npAP, as *G. anatis* frequently co-inhabits the same host, as previously documented (Rocha et al., 2024; Mei et al., 2026). Further HGT analyses are needed to elucidate the potential role of genetic exchange in shaping the npAP HMTp210 architecture.

As the first 1,200 bp of the *HMTp210* gene has sufficient discriminatory power to differentiate among the three AP serogroups A, B, and C (Tan et al., 2021; Buter et al., 2023), we constructed a phylogenetic tree based on this region. The analysis revealed that the npAP isolates formed two distinct clusters. The first npAP cluster was positioned close to serogroup B, suggesting a possible evolutionary relationship between this npAP group and serogroup B. In contrast, the second npAP cluster was clearly distinct from serogroups A, B, and C, which may indicate the presence of an additional serogroup. This observation requires further laboratory confirmation, such as hemagglutination inhibition (HI) testing, to validate whether these isolates indeed represent a previously unrecognized serogroup.

Similar to many other pathogenic bacteria, the capsular polysaccharide is a major virulence factor of AP (Sawata and Kume, 1983; Sawata et al., 1985; Tu et al., 2015; Chen et al., 2024), protecting the bacteria against phagocytosis and complement-mediated killing (Boulnois and Roberts, 1990; Whitfield, 2006). Genomic comparisons in the current study revealed that the capsular polysaccharide locus is present in all pAP strains but absent in all npAP strains, except for four npAP genomes harboring this locus, including an intact *hctA* gene. The *hctA* findings highlight a limitation of the previously published differential qPCR assays (Shelkamy et al., 2024), in which this gene was selected as a marker for pAP. However, *hctA* is no longer considered a unique marker for pAP, and new differential qPCR assays based on robust markers need to be developed and validated.

Notably, these four npAP genomes contain a consistent defect in one of the biosynthesis genes, *ccbE*, which contains a frameshift leading to a premature stop codon, strongly suggesting loss of normal *ccbE* function. The *ccbE* gene is homologous to *kfiB* in *Escherichia coli* K5 and *dcbE* in *Pasteurella multocida* type D (Wu et al., 2010; Townsend et al., 2001). In *E. coli*, KfiB stabilizes the KfiAC complex, facilitates its transport to the inner membrane, and contributes to heparosan export and capsule biosynthesis initiation (Leroux and Priem, 2016; Willis and Whitfield, 2013). Based on these functional and sequence similarities, our hypothesis is that disruption of *ccbE* in these npAP isolates is likely to impair normal capsule biosynthesis and may contribute to the absence or alteration of capsule production in these strains.

Regarding the defects observed in *ccbE* of the pathogenic strains ESV-135 (CP050316) and 221 (AOGF00000000), these findings could be attributable to sequencing or assembly errors, as both the PacBio Sequel platform (Tedersoo et al., 2018) and SOAPdenovo assembler (Meng et al., 2014) have been associated with potential errors. Furthermore, no published experimental data are available to confirm the presence or absence of capsule production in these strains. It is worth noting that the genome assembly of strain 221 used in the current study (AOGF00000000) is completely different from the strain 221 described by Wu et al. (2010) with respect to the capsular locus. Wu et al. reported an intact genotype I capsular locus, whereas the genome analyzed in the current study contains defects and is more similar to genotype II. The reason for this discrepancy remains unclear; it may be due to subpopulation differences, a mixed culture, or a labeling issue during genome submission (Xu et al., 2013).

In contrast, the *ccbE* defect identified in the four npAP genomes is supported by strong evidence. The same 35-nucleotide deletion was consistently identified in all four isolates, despite their recovery from two different sites. Furthermore, this deletion was independently confirmed by conventional PCR and Sanger sequencing, eliminating concerns regarding sequencing accuracy. Nevertheless, although the sequence defect is well supported, its effect on capsule production in *Avibacterium paragallinarum* remains hypothetical and requires experimental validation. Future studies should investigate capsule production through direct visualization in the four npAP isolates, as well as in strains ESV-135 and 221.

Hmp is involved in nitric oxide detoxification (NOD), thereby playing a central role in the inducible response to nitrosative stress and in protecting bacteria from nitric-oxide–mediated killing by macrophages (Hausladen et al., 2001; Gardner and Gardner, 2002; Stevanin et al., 2002; UniProt Consortium, 2023). The NOD reaction is tightly regulated by NsrR (Bang et al., 2006; Karlinsey et al., 2012). The roles of Hmp and NsrR in *Salmonella* virulence and pathogenesis have been well established (Bang et al., 2006; Gilberthorpe et al., 2007; Karlinsey et al., 2012) and the contribution of Hmp to *Staphylococcus aureus* virulence has also been demonstrated (Richardson et al., 2006). Given the established role of the Hmp/NsrR system in protecting bacteria from host-derived nitric oxide stress in other bacterial species, its absence in npAP may impair the ability of these isolates to withstand innate immune defenses, providing a plausible mechanistic hypothesis for their nonpathogenic phenotype. However, the function of this operon in AP remains to be experimentally validated.

FtsZ is a cytosolic tubulin-like protein that plays an essential role in bacterial cell division (Löwe and Amos, 1998). Although traditionally considered a cytosolic protein, emerging evidence suggests that FtsZ may also exhibit moonlighting functions related to host interaction and immunogenicity in some bacterial species (Gallotta et al., 2014; Feng et al., 2018; Fu et al., 2022; Jurado-Martín et al., 2024; Jurado-Martín et al., 2024). Collectively, these findings support the idea that FtsZ may function as a bacterial virulence-associated protein with moonlighting roles beyond cell division.

In this study, npAP isolates exhibited consistent allelic divergence within the FtsZ C-terminal region relative to pAP isolates, suggesting potential functional divergence. Although the precise functional role of the C-terminal domain of FtsZ in AP remains unknown, studies in other bacteria have shown that this region is involved in the recruitment of downstream cell division proteins and regulation of bacterial cytokinesis (Ma and Margolin, 1999; Jindal and Panda, 2013; Sundararajan and Goley, 2017). The biological significance of the observed amino acid substitutions in npAP remains unknown. However, the consistent divergence within the C-terminal region raises the possibility that these substitutions may influence FtsZ function and, consequently, bacterial physiology, including cell division or replication efficiency. Previous challenge studies demonstrated that npAP exhibited a lower bacterial load than pAP over a 1-week post-challenge period, as determined by qPCR (Shelkamy et al., 2026). Although this observation cannot be directly attributed to the observed FtsZ allelic differences, it is consistent with the hypothesis that alterations in FtsZ may contribute to the nonpathogenic phenotype of npAP. Nevertheless, functional studies are required to determine whether these sequence differences affect FtsZ activity and contribute to the reduced virulence of npAP.

Based on the four previously discussed consistent genetic findings, we hypothesize that defects or alterations in HMTp210 adhesin, the capsular polysaccharide locus, the nitric oxide dioxygenase system, and/or FtsZ may contribute to the nonpathogenic phenotype of npAP in chickens. However, these proposed mechanisms remain hypothetical and require further experimental validation. Future studies, including targeted gene manipulation and live bird challenge experiments, will be necessary to determine the specific contribution of each factor to the npAP phenotype. In addition, phenotypic assays, such as haemagglutination assays to assess HMTp210 functionality, capsule visualization to evaluate the impact of *ccbE* defects, and nitric oxide resistance assays to investigate Hmp/NsrR-associated functions, will be critical for testing these hypotheses.

Interestingly, we identified a gene encoding a putative α-2,3 sialyltransferase (CMP-N-acetylneuraminate-β-galactosamide-α-2,3-sialyltransferase, EC 2.4.99.–) that is exclusively present in npAP and absent in all pAP genomes analyzed in the current study. This enzyme contributes to the sialylation of lipooligosaccharide, a modification that can help bacteria evade host immune detection and can serve as a metabolic substrate to support colonization and persistence in specific host niches (Vimr, 2013; McDonald et al., 2016; North et al., 2018; Severi et al., 2021). Based on this, we hypothesize that npAP may possess a sialylated lipooligosaccharide that promotes a more commensal-like interaction with the host, reducing immune activation and preventing overt inflammatory responses, however further experimental validation is needed to test this hypothesis. A similar pattern has been reported in *Haemophilus parasuis*, where this enzyme is encoded by avirulent strains but absent in virulent strains, and loss of sialylation has been associated with heightened host sensitivity (Lawrence et al., 2014).

It is also noteworthy that the *ompP4* gene, encoding outer membrane lipoprotein e (P4)/NMN 5′-nucleotidase, was present in all pAP genomes but absent from all npAP genomes except npAP/GA-USA/20231216/S1-1. In *Haemophilus influenzae*, lipoprotein e (P4) is involved in NAD and NADP utilization, heme acquisition, host invasion, adhesion, and serum resistance (Reidl et al., 2000; Morton et al., 2007; Su et al., 2016). We hypothesize that the absence of *ompP4* may contribute to the nonpathogenic phenotype of npAP and could also impair NAD utilization, potentially explaining the lower recovery of npAP from field samples using standard NAD-supplemented media. Consistent with this hypothesis, *H. influenzae* strains lacking functional lipoprotein e (P4) exhibit reduced growth on NAD-supplemented media (Su et al., 2016). However, the presence of an apparently intact *ompP4* gene in npAP/GA-USA/20231216/S1-1 indicates that further functional validation is required before generalizing this hypothesis.

The remaining virulence factors identified from the literature and VFDB (Table 1) were analyzed following the same approach; however, no major consistent differences were observed between npAP and pAP. Nevertheless, several noteworthy findings related to RTX, virulence-associated protein D, *lic1ABCD* operon, F17-like fimbriae, and heme utilization system were discussed in Supplementary Table 9, which may provide valuable insights for the AP research community.

In conclusion, this study provides a comprehensive comparative genomic analysis between pAP and naturally occurring npAP isolates to identify genetic features associated with the nonpathogenic phenotype of npAP. Collectively, the genomic differences identified in this study suggest that the pathogenicity of AP is unlikely to depend on a single virulence factor. Our findings suggest that alterations in factors related to adhesion (HMTp210), immune evasion (capsule, and the nitric oxide dioxygenase system), and cell division (FtsZ), along with the absence of OmpP4 and presence of a putative sialyltransferase, may contribute to the nonpathogenic phenotype of npAP. However, laboratory validation, including mutant construction, live-bird challenge studies, and phenotypic assays, is required to determine the specific contribution of these factors to pathogenicity. While the genomic differences identified in this study provide plausible explanations for the nonpathogenic phenotype of npAP, alternative explanations should also be considered. The observed phenotype may result from the combined effects of multiple genomic, regulatory, and environmental factors rather than individual genetic features alone.

We acknowledge the limitation of including only 14 npAP genomes originating from the United States; therefore, the full extent and global diversity of the npAP population remain to be determined. We also acknowledge the uncertainty associated with inferring gene absence from draft genomes. Although stringent thresholds were applied, factors such as assembly quality and contig fragmentation may influence gene detection and the confidence of absence calls.

## Data Availability

The datasets presented in this study can be found in online repositories. The names of the repository/repositories and accession number(s) can be found in the article/Supplementary material.

## Footnotes

1
https://www.ncbi.nlm.nih.gov/Structure/wrpsb-out/wrpsb.cgi

2
https://www.bv-brc.org/

## Supplementary material

Supplementary Figure 1 Multiple amino acid sequence alignment generated using MAFFT in MegAlign Pro (DNASTAR Navigator v15.0.1.1). Representative pAP HMTp210 proteins are shown (NCTC10926-A1, HP60-C4, Spross-B1, SA3-C3, ESV-135-C1, Modesto-C2, NCTC11296-A2, E-3C [HVR-A3], and HP-14 [HVR-A4]) in comparison with the npAP HMTp210 sequences. E-3C and HP-14 contain only partial HMTp210 sequences corresponding to the hypervariable region (HVR). Green regions represent aligned sequences, whereas gray regions indicate gaps (absence of sequence).
