# Supplementary material for: Comparative genomics and in silico virulence assessment of nonpathogenic Avibacterium paragallinarum isolates from healthy layer chickens
Source: Front Microbiol. 2026 Sep 3;17:1919963. doi: 10.3389/fmicb.2026.1919963 (PMC13582619; doi:10.3389/fmicb.2026.1919963)
Supplement: Supplementary file 1 [file Table_1.docx]

**Supplementary Table 1: The List of the Analyzed *Avibacterium paragallinarum* Genomes**

| Strain/Isolate^a^ | Pathogenicity^b^ | NCBI Accession no. | Assembly status | Quast | | | CheckM completeness (%) | CheckM contamination (%) | Original isolate location/year | *In silico* serovar prediction^c^ |
| --- | --- | --- | --- | --- | --- | --- | --- | --- | --- | --- |
|  |  |  |  | Number of contigs | GC content (%) | Contig N50 |  |  |  |  |
| npAP/IA-USA/20210120/S1-1 (AP-2)* | Nonpathogenic | CP104917 | complete | 1 | 40.99 | 2,529,853 | 98.8 | 1.3 | United States/2021 | NA |
| npAP/OH-USA/20210309/S1-1 (AG21-0333)* | Nonpathogenic | CP104914 | complete | 1 | 40.96 | 2,399,548 | 98.4 | 1.2 | United States/2021 | NA |
| npAP/OH-USA/20230213/S1-2* | Nonpathogenic | CP173233 | complete | 1 | 41.07 | 2,423,524 | 99.3 | 1.3 | United States/2023 | NA |
| npAP/OH-USA/20230213/S1-3* | Nonpathogenic | CP173232 | complete | 1 | 41 | 2,425,817 | 99 | 1.1 | United States/2023 | NA |
| npAP/OH-USA/20230213/S1-4* | Nonpathogenic | CP173231 | complete | 1 | 40.99 | 2,419,151 | 99 | 1.1 | United States/2023 | NA |
| npAP/GA-USA/20231216/S1-1 | Nonpathogenic | CP173236 | complete | 1 | 41.04 | 2,521,590 | 99.4 | 0.9 | United States/2023 | NA |
| npAP/GA-USA/20231216/S1-2 | Nonpathogenic | CP173230 | complete | 1 | 41 | 2,506,457 | 98.8 | 0.8 | United States/2023 | NA |
| npAP/GA-USA/20231220/S2-1 | Nonpathogenic | CP173229 | complete | 1 | 41.07 | 2,517,499 | 99.3 | 1.1 | United States/2023 | NA |
| npAP/GA-USA/20231220/S2-3 | Nonpathogenic | CP173228 | complete | 1 | 41.14 | 2,532,146 | 98.9 | 1.1 | United States/2023 | NA |
| npAP/NC-USA/20240110/S1-1 | Nonpathogenic | CP173234 | complete | 1 | 41.1 | 2,464,160 | 99.5 | 0.9 | United States/2024 | NA |
| npAP/GA-USA/20231220/S2-2 | Nonpathogenic | JBJBHD000000000 | Draft | 3 | 41.08 | 2,268,408 | 99.3 | 1 | United States/2023 | NA |
| npAP/GA-USA/20231220/S2-4 | Nonpathogenic | JBJBHC000000000 | Draft | 3 | 41.23 | 1,955,296 | 98.9 | 1.2 | United States/2023 | NA |
| npAP/OH-USA/20230315/S2-1* | Nonpathogenic | JBJBHF000000000 | Draft | 34 | 41.15 | 176,515 | 98.4 | 1.8 | United States/2023 | NA |
| npAP/FL-USA/20231025/S1-1 | Nonpathogenic | JBJBHE000000000 | Draft | 213 | 41.51 | 19,604 | 98 | 1 | United States/2023 | NA |
| *Avibacterium paragallinarum* Field isolate 1 | Pathogenic | JBREWG000000000 | Draft | 159 | 40.89 | 43,034 | 99 | 0.7 | United States/2023 | C-1 |
| *Avibacterium paragallinarum* Field isolate 2 | Pathogenic | JBREWH000000000 | Draft | 92 | 40.84 | 69,597 | 99 | 0.7 | United States/2023 | C-1 |
| *Avibacterium paragallinarum* Field isolate 3 | Pathogenic | JBREWI000000000 | Draft | 176 | 40.93 | 33,816 | 99 | 0.7 | United States/2023 | C-1 |
| *Avibacterium paragallinarum* Field isolate 4 | Pathogenic | JBREWJ000000000 | Draft | 186 | 40.93 | 27,167 | 99 | 0.8 | United States/2023 | C-1 |
| *Avibacterium paragallinarum* Field isolate 5 | Pathogenic | JBREWK000000000 | Draft | 99 | 40.81 | 71,576 | 99 | 0.6 | United States/2023 | C-1 |
| *Avibacterium paragallinarum* NCTC11296 | Pathogenic | UGHK00000000 | Draft | 3 | 41.13 | 1,867,593 | 100 | 1.8 | Germany/- | A-2 |
| *Avibacterium paragallinarum* ESV-135 | Pathogenic | CP050316 | complete | 1 | 40.88 | 2,521,134 | 98.28 | 1.29 | Mexico/2008 | C-1 |
| *Avibacterium paragallinarum* ADL-AP17 | Pathogenic | CP051636 | complete | 1 | 40.91 | 2,415,699 | 98.45 | 0.49 | United States/2019 | C-1 |
| *Avibacterium paragallinarum* AVPG2015 | Pathogenic | LAEO00000000 | Draft | 24 | 40.86 | 268,011 | 100 | - | Mexico/2014 | C-1 |
| *Avibacterium paragallinarum* FARPER-174 | Pathogenic | CP034110 | complete | 1 | 40.86 | 2,425,949 | 98.36 | 2.63 | Peru/2015 | **B-1** |
| *Avibacterium paragallinarum* ADL-AP16 | Pathogenic | CP051637 | complete | 1 | 40.91 | 2,415,855 | 98.45 | 0.49 | United States/2019 | C-1 |
| *Avibacterium paragallinarum* ADL-AP15 | Pathogenic | CP051638 | complete | 1 | 40.91 | 2,415,950 | 98.45 | 0.65 | United States/2019 | C-1 |
| *Avibacterium paragallinarum* ADL-AP07 | Pathogenic | CP051640 | complete | 1 | 40.91 | 2,415,993 | 98.45 | 0.57 | United States/2019 | C-1 |
| *Avibacterium paragallinarum* ADL-AP01 | Pathogenic | CP051642 | complete | 1 | 40.91 | 2,415,542 | 98.45 | 0.49 | United States/2019 | C-1 |
| *Avibacterium paragallinarum* ADL-AP02 | Pathogenic | CP051641 | complete | 1 | 40.91 | 2,416,187 | 98.45 | 0.57 | United States/2019 | C-1 |
| *Avibacterium paragallinarum* ADL-AP10 | Pathogenic | CP051639 | complete | 1 | 40.91 | 2,415,552 | 98.45 | 0.57 | United States/2019 | C-1 |
| *Avibacterium paragallinarum* p4chr1 | Pathogenic | CP081939 | complete | 1 | 41.01 | 2,774,989 | 98.69 | 2 | China/2021 | A-1 |
| *Avibacterium paragallinarum* ZJ-C | Pathogenic | CP095161 | complete | 1 | 40.93 | 2,675,016 | 99.3 | 2.3 | China/2019 | C-4 |
| *Avibacterium paragallinarum* C-AP1 | Pathogenic | CP113786 | complete | 1 | 40.92 | 2,614,420 | 99.3 | 0.7 | China/2021 | A-1 |
| *Avibacterium paragallinarum* C-AP4 | Pathogenic | CP113955 | complete | 1 | 40.79 | 2,592,260 | 99.3 | 0.7 | China/2022 | C-4 |
| *Avibacterium paragallinarum* 221 | Pathogenic | AOGF00000000 | Draft | 135 | 41 | 71,093 | 100 | 4.4 | Japan/- | **B-1** |
| *Avibacterium paragallinarum* JF4211 | Pathogenic | CBMK00000000 | Draft | 34 | 41.1 | 168,697 | 100 | 6.9 | Switzerland/- | A-2 |
| *Avibacterium paragallinarum* SCPM-O-B-8406 | Pathogenic | QJPJ00000000 | Draft | 67 | 40.81 | 132,106 | 100 | 1.8 | Russia/2017 | A-2 |
| *Avibacterium paragallinarum* SCPM-O-B-8407 | Pathogenic | QJPI00000000 | Draft | 66 | 40.81 | 133,552 | 100 | 1.8 | Russia/2016 | A-2 |
| *Avibacterium paragallinarum* NCTC10926 | Pathogenic | UFSW00000000 | Draft | 4 | 41.15 | 2,102,832 | 100 | 4.5 | -/1958 | A-1 |
| *Avibacterium paragallinarum* 2671 | Pathogenic | RQXQ00000000 | Draft | 135 | 41.02 | 32,637 | 98.53 | 1.61 | Germany/- | **A-1** |
| *Avibacterium paragallinarum* SA-3 | Pathogenic | RQXS00000000 | Draft | 139 | 41.03 | 33,022 | 98.45 | 1.93 | South Africa/- | C-3 |
| *Avibacterium paragallinarum* Modesto | Pathogenic | RQXR00000000 | Draft | 138 | 40.89 | 36,593 | 98.12 | 1.05 | United States/- | C-2 |
| *Avibacterium paragallinarum* AVPG 221 | Pathogenic | RQXP00000000 | Draft | 82 | 41.06 | 52,835 | 99.1 | 1.45 | Japan/- | **B-1** |
| *Avibacterium paragallinarum* Hp8 | Pathogenic | VXDA00000000 | Draft | 182 | 40.99 | 24,809 | 98.45 | 1.53 | China/2018 | A-1 |
| *Avibacterium paragallinarum* Z2S-1-2 | Pathogenic | JACEWC000000000 | Draft | 122 | 40.77 | 126,722 | 98.69 | 0.65 | China/2019 | C-4 |
| *Avibacterium paragallinarum* Z1N-2-2 | Pathogenic | JACEWA000000000 | Draft | 128 | 40.78 | 126,722 | 98.69 | 0.65 | China/2019 | C-4 |
| *Avibacterium paragallinarum* Z1N-1-2 | Pathogenic | JACEVY000000000 | Draft | 124 | 40.77 | 96,992 | 98.69 | 0.65 | China/2019 | C-4 |
| *Avibacterium paragallinarum* Y2S-2 | Pathogenic | JACEVX000000000 | Draft | 65 | 40.84 | 105,438 | 98.69 | 0.81 | China/2019 | A-2 |
| *Avibacterium paragallinarum* Z1N-2-1 | Pathogenic | JACEVZ000000000 | Draft | 125 | 40.77 | 96,992 | 98.69 | 0.65 | China/2019 | C-4 |
| *Avibacterium paragallinarum* APX1-1S-1 | Pathogenic | JACEVT000000000 | Draft | 124 | 40.78 | 141,227 | 98.69 | 0.65 | China/2019 | C-4 |
| *Avibacterium paragallinarum* APX2-2N-2 | Pathogenic | JACEVV000000000 | Draft | 125 | 40.78 | 126,721 | 98.69 | 0.65 | China/2019 | C-4 |
| *Avibacterium paragallinarum* APX3-2S-2 | Pathogenic | JACEVW000000000 | Draft | 127 | 40.77 | 126,721 | 98.69 | 0.65 | China/2019 | C-4 |
| *Avibacterium paragallinarum* APX1-2N-1 | Pathogenic | JACEVU000000000 | Draft | 128 | 40.78 | 133,348 | 98.69 | 0.65 | China/2019 | C-4 |
| *Avibacterium paragallinarum* APX1-1N-2 | Pathogenic | JACEVS000000000 | Draft | 126 | 40.78 | 126,721 | 98.69 | 0.65 | China/2019 | C-4 |
| *Avibacterium paragallinarum* AP12-5S-1 | Pathogenic | JACEVR000000000 | Draft | 119 | 40.78 | 126,721 | 98.69 | 0.65 | China/2019 | C-4 |
| *Avibacterium paragallinarum* AP12-4N-1 | Pathogenic | JACEVQ000000000 | Draft | 119 | 40.77 | 141,221 | 98.69 | 0.65 | China/2019 | C-4 |
| *Avibacterium paragallinarum* AP1-3N-1 | Pathogenic | JACEVO000000000 | Draft | 124 | 40.78 | 97,526 | 98.69 | 0.65 | China/2019 | C-4 |
| *Avibacterium paragallinarum* AP1-2N-1 | Pathogenic | JACEVN000000000 | Draft | 123 | 40.78 | 126,721 | 98.69 | 0.65 | China/2019 | C-4 |
| *Avibacterium paragallinarum* AP1-3S-1 | Pathogenic | JACEVP000000000 | Draft | 122 | 40.78 | 141,222 | 98.69 | 0.65 | China/2019 | C-4 |
| *Avibacterium paragallinarum* AP1-1N-1 | Pathogenic | JACEVM000000000 | Draft | 120 | 40.78 | 133,348 | 98.69 | 0.65 | China/2019 | C-4 |
| *Avibacterium paragallinarum* omv-p4chr1 | Pathogenic | JAJAGI000000000 | Draft | 162 | 40.90 | 64,861 | 98.69 | 1.13 | China/2021 | A-1 |
| *Avibacterium paragallinarum* CCUG 12835 | Pathogenic | PQVK00000000 | Draft | 335 | 41.02 | 19,158 | 100 | 4 | Germany/1970 | A-2 |
| *Avibacterium paragallinarum* BR | Pathogenic | JAZDVC000000000 | Draft | 129 | 40.84 | 34,241 | 99.2 | 0.9 | Russia/2015 | A-2 |
| *Avibacterium paragallinarum* Center | Pathogenic | JAZDVD000000000 | Draft | 165 | 40.90 | 30,097 | 99.2 | 1.1 | Russia/2018 | A-2 |
| *Avibacterium paragallinarum* UR | Pathogenic | JAZDVF000000000 | Draft | 79 | 40.83 | 76,952 | 99.2 | 1 | Russia/2018 | A-2 |
| *Avibacterium paragallinarum* KS | Pathogenic | JAZDVE000000000 | Draft | 58 | 40.81 | 124,951 | 99.2 | 0.9 | Russia/2018 | A-2 |
| *Avibacterium paragallinarum* VR | Pathogenic | JAZDVG000000000 | Draft | 59 | 40.82 | 117,842 | 99.2 | 0.9 | Russia/2018 | A-2 |
| *Avibacterium paragallinarum* AV38 | Pathogenic | JAMDJS000000000 | Draft | 98 | 40.82 | 92,668 | 99.3 | 1.1 | China/2022 | C-4 |
| *Avibacterium paragallinarum* AV33 | Pathogenic | JAMDJX000000000 | Draft | 164 | 40.81 | 33,082 | 99.3 | 3.4 | China/2022 | C-4 |
| *Avibacterium paragallinarum* AV32 | Pathogenic | JAMDJY000000000 | Draft | 117 | 40.82 | 48,847 | 99.3 | 1.8 | China/2022 | C-4 |
| *Avibacterium paragallinarum* AV34 | Pathogenic | JAMDJW000000000 | Draft | 179 | 40.81 | 28,925 | 99.3 | 4.3 | China/2022 | C-4 |
| *Avibacterium paragallinarum* AV35 | Pathogenic | JAMDJV000000000 | Draft | 100 | 40.81 | 95,407 | 99.3 | 0.9 | China/2022 | C-4 |
| *Avibacterium paragallinarum* AV31 | Pathogenic | JAMDJZ000000000 | Draft | 123 | 40.81 | 56,091 | 99.3 | 2 | China/2022 | C-4 |
| *Avibacterium paragallinarum* AV29 | Pathogenic | JAMDKB000000000 | Draft | 71 | 40.85 | 67,111 | 99.3 | 0.8 | China/2022 | A-2 |
| *Avibacterium paragallinarum* AV27 | Pathogenic | JAMDKD000000000 | Draft | 68 | 40.85 | 92,231 | 99.3 | 0.9 | China/2022 | A-2 |
| *Avibacterium paragallinarum* AV28 | Pathogenic | JAMDKC000000000 | Draft | 64 | 40.85 | 88,136 | 99.3 | 0.8 | China/2022 | A-2 |
| *Avibacterium paragallinarum* AV26 | Pathogenic | JAMDKE000000000 | Draft | 107 | 40.87 | 64,234 | 99.3 | 1.2 | China/2022 | A-1 |
| *Avibacterium paragallinarum* AV23 | Pathogenic | JAMDKH000000000 | Draft | 69 | 40.85 | 66,280 | 99.3 | 0.9 | China/2022 | A-2 |
| *Avibacterium paragallinarum* AV30 | Pathogenic | JAMDKA000000000 | Draft | 208 | 40.81 | 28,288 | 99.3 | 4.4 | China/2022 | C-4 |
| *Avibacterium paragallinarum* AV21 | Pathogenic | JAMDKJ000000000 | Draft | 102 | 40.81 | 70,647 | 99.3 | 0.9 | China/2022 | C-4 |
| *Avibacterium paragallinarum* AV25 | Pathogenic | JAMDKF000000000 | Draft | 95 | 40.88 | 92,887 | 99.3 | 1 | China/2022 | A-1 |
| *Avibacterium paragallinarum* AV24 | Pathogenic | JAMDKG000000000 | Draft | 111 | 40.88 | 62,992 | 99.3 | 1.9 | China/2022 | A-1 |
| *Avibacterium paragallinarum* AV18 | Pathogenic | JAMDKM000000000 | Draft | 128 | 40.78 | 126,722 | 99.3 | 0.7 | China/2022 | C-4 |
| *Avibacterium paragallinarum* AV22 | Pathogenic | JAMDKI000000000 | Draft | 101 | 40.81 | 66,002 | 99.3 | 1 | China/2022 | C-4 |
| *Avibacterium paragallinarum* AV19 | Pathogenic | JAMDKL000000000 | Draft | 94 | 40.81 | 91,875 | 99.3 | 1 | China/2022 | C-4 |
| *Avibacterium paragallinarum* AV17 | Pathogenic | JAMDKN000000000 | Draft | 124 | 40.77 | 96,992 | 99.3 | 0.7 | China/2022 | C-4 |
| *Avibacterium paragallinarum* AV16 | Pathogenic | JAMDKO000000000 | Draft | 119 | 40.77 | 141,221 | 99.3 | 0.7 | China/2022 | C-4 |
| *Avibacterium paragallinarum* AV15 | Pathogenic | JAMDKP000000000 | Draft | 122 | 40.77 | 126,722 | 99.3 | 0.7 | China/2022 | C-4 |
| *Avibacterium paragallinarum* AV13 | Pathogenic | JAMDKR000000000 | Draft | 125 | 40.77 | 96,992 | 99.3 | 0.7 | China/2022 | C-4 |
| *Avibacterium paragallinarum* AV14 | Pathogenic | JAMDKQ000000000 | Draft | 65 | 40.84 | 105,438 | 99.3 | 0.8 | China/2022 | A-2 |
| *Avibacterium paragallinarum* AV8 | Pathogenic | JAMDKW000000000 | Draft | 127 | 40.77 | 126,721 | 99.3 | 0.7 | China/2022 | C-4 |
| *Avibacterium paragallinarum* AV10 | Pathogenic | JAMDKU000000000 | Draft | 124 | 40.78 | 97,526 | 99.3 | 0.7 | China/2022 | C-4 |
| *Avibacterium paragallinarum* AV9 | Pathogenic | JAMDKV000000000 | Draft | 125 | 40.78 | 126,721 | 99.3 | 0.7 | China/2022 | C-4 |
| *Avibacterium paragallinarum* AV6 | Pathogenic | JAMDKY000000000 | Draft | 123 | 40.78 | 126,721 | 99.3 | 0.7 | China/2022 | C-4 |
| *Avibacterium paragallinarum* AV4 | Pathogenic | JAMDLA000000000 | Draft | 122 | 40.78 | 141,222 | 99.3 | 0.7 | China/2022 | C-4 |
| *Avibacterium paragallinarum* AV5 | Pathogenic | JAMDKZ000000000 | Draft | 120 | 40.78 | 133,348 | 99.3 | 0.7 | China/2022 | C-4 |
| *Avibacterium paragallinarum* AV7 | Pathogenic | JAMDKX000000000 | Draft | 128 | 40.78 | 133,348 | 99.3 | 0.7 | China/2022 | C-4 |
| *Avibacterium paragallinarum* AV2 | Pathogenic | JAMDLC000000000 | Draft | 124 | 40.78 | 141,227 | 99.3 | 0.7 | China/2022 | C-4 |
| *Avibacterium paragallinarum* AV3 | Pathogenic | JAMDLB000000000 | Draft | 119 | 40.78 | 126,721 | 99.3 | 0.7 | China/2022 | C-4 |
| *Avibacterium paragallinarum* AV1 | Pathogenic | JAMDLD000000000 | Draft | 126 | 40.78 | 126,721 | 99.3 | 0.7 | China/2022 | C-4 |
| *Avibacterium paragallinarum* HP60 | Pathogenic | JBANLW000000000 | Draft | 305 | 40.86 | 26,856 | 98.9 | 1.3 | South Africa/2023 | C-4 |

^a^Naming format for nonpathogenic *Avibacterium paragallinarum* isolates: npAP/location/YearMonthDay/site-isolate number. *refers to the npAP isolates used for the identification of differential qPCR targets described by Shelkamy et al. (2024).^b^nonpathogenic isolates obtained from naïve healthy layer chicken flocks, while pathogenic isolates obtained from diseased chicken flocks. ^c^The serovar of the included genome sequences was predicted according to Buter et al. (2023), “Molecular characterization of the *HMTp210* gene of *Avibacterium paragallinarum* and the proposition of a new genotyping method as an alternative to classical serotyping.” Avian Pathology 52, no. 5 (2023): 362–376. Serovars highlighted in red indicate mispredicted serovars, and these genomes were more likely mislabeled by the submitters to NCBI. All 101 genomes included in this study were obtained from the NCBI Genomes database, comprising 14 npAP genomes and 5 pAP genomes (Field isolates 1-5) generated by our group, together with 82 publicly available assembled pAP genomes generated by other research groups. (-) unknown data.
