# Supplementary material for: Comparative genomics and in silico virulence assessment of nonpathogenic Avibacterium paragallinarum isolates from healthy layer chickens
Source: Front Microbiol. 2026 Sep 3;17:1919963. doi: 10.3389/fmicb.2026.1919963 (PMC13582619; doi:10.3389/fmicb.2026.1919963)
Supplement: Supplementary file 2 [file Table_10.docx]

**Supplementary Table 10. Summary of the analyzed virulence-associated genes identified using VFDB**

| Gene | Product | EC Number | BV-BRC ID**^a^** | pAP**^b^** | npAP | Important sites**^c^** | Protein domain**^d^** |
| --- | --- | --- | --- | --- | --- | --- | --- |
| *kdsA* | 2-Keto-3-deoxy-D-manno-octulosonate-8-phosphate synthase | EC 2.5.1.55 | **fig\|728.107.peg.641** | Present (87) | Present (13) | None | **IPR006218** |
| *rfaD* | ADP-L-glycero-D-manno-heptose-6-epimerase | EC 5.1.3.20 | **fig\|728.107.peg.2098** | Present (87) | Present (14) | Active sites (N93 – S116 – Y139 – K143), NADP binding sites (T5 – G6 - G9 – F10 – I11 – 30VDD – K38 – K53 – 75EGA – S79 – F88 – N92 – Y96 – 114ASS – Y139 – K143 – 166YF – V169 – 176HK), Substrate binding sites (T81 – N168 – 179SMA – V183 – H186 – R208 – F242) | IPR001509 |
| *IpxA* | Acyl-[acyl-carrier-protein]--UDP-N-acetylglucosamine O-acyltransferase | EC 2.3.1.129 | **fig\|728.107.peg.1718** | Present (87) | Present (14) | None | IPR056729  IPR001451  IPR029098 |
| *KatA* | Catalase KatE | EC 1.11.1.6 | fig\|728.352.peg.1751 | Absent (87) | Present (4) | None | IPR011614  IPR010582 |
| *rfaE* | D-glycero-beta-D-manno-heptose 1-phosphate adenylyltransferase / D-glycero-beta-D-manno-heptose-7-phosphate kinase | EC 2.7.7.70/  EC 2.7.1.167 | fig\|728.107.peg.700 | Present (87) | Present (14) | None | IPR011611  IPR004821 |
| *gmhA/lpcA* | D-sedoheptulose 7-phosphate isomerase | EC 5.3.1.28 | fig\|728.107.peg.1923 | Present (87) | Present (14) | Active sites (52NGG – 119STS – S124 – Y167 –Q172) | IPR001347 |
| *gmd* | GDP-mannose 4,6-dehydratase | EC 4.2.1.47 | fig\|728.107.peg.2586 | Present (81) | Present (13) | None | IPR016040 |
| *msbB* | Lipid A biosynthesis myristoyltransferase | EC 2.3.1.243 | fig\|728.107.peg.1291 | Present (87) | Present (14) | Putative acyl-acceptor binding pocket sites (144H -147A – 149D -166YNPH – 215EDY) | IPR004960 |
| *lpxB* | Lipid-A-disaccharide synthase | EC 2.4.1.182 | fig\|728.107.peg.1717 | Present (87) | Present (14) | None | IPR003835 |
| *lpxC* | UDP-3-O-[3-hydroxymyristoyl] N-acetylglucosamine deacetylase | EC 3.5.1.108 | fig\|728.107.peg.987 | Present (87) | Present (14) | None | IPR004463 |
| *lpxD* | UDP-3-O-[3-hydroxymyristoyl] glucosamine N-acyltransferase | EC 2.3.1.191 | fig\|728.107.peg.1720 | Present (87) | Present (14) | None | IPR020573  IPR001451 |
| *galE* | UDP-glucose 4-epimerase | EC 5.1.3.2 | fig\|728.107.peg.2246 | Present (87) | Present (14) | None | IPR001509 |
| *gaIU* | UTP--glucose-1-phosphate uridylyltransferase | EC 2.7.7.9 | fig\|728.107.peg.2980 | Present (87) | Present (14) | None | IPR005835 |
| *wecA* | Undecaprenyl-phosphate alpha-N-acetylglucosaminyl 1-phosphate transferase | EC 2.7.8.33 | fig\|728.107.peg.829 | Present (87) | Present (14) | None | IPR000715 |

^a^The BV-BRC ID of AP type strain NCTC11296 was used for all genes, except for *KatA,* for which the ID of isolate AG21-0333 was used. Typing this ID in the BV-BRC search bar <https://www.bv-brc.org/>, provides access to the nucleotide and amino acid sequences of each gene. ^b^Numbers in parentheses in both pAP and npAP columns indicate the number of genomes. ^c^Important sites were identified using NCBI CDD <https://www.ncbi.nlm.nih.gov/Structure/wrpsb-out/wrpsb.cgi>; “None” indicates that no important sites were detected. ^d^The integrated accession number for the representative protein domain was obtained from the InterPro server.
