# Supplementary material for: Comparative genomics and in silico virulence assessment of nonpathogenic Avibacterium paragallinarum isolates from healthy layer chickens
Source: Front Microbiol. 2026 Sep 3;17:1919963. doi: 10.3389/fmicb.2026.1919963 (PMC13582619; doi:10.3389/fmicb.2026.1919963)
Supplement: Supplementary file 3 [file Table_2.docx]

**Supplementary Table 2: The 557 gene products used to construct the bacterial phylogenomic tree**

| **Serial** | **PGFam** | **Gene Product** |
| --- | --- | --- |
| 1 | PGF_00416410 | Carbamoyl-phosphate synthase large chain (EC 6.3.5.5) |
| 2 | PGF_05237064 | RND efflux system, inner membrane transporter |
| 3 | PGF_00045982 | Pyruvate dehydrogenase E1 component (EC 1.2.4.1) |
| 4 | PGF_09084129 | Acetaldehyde dehydrogenase (EC 1.2.1.10) / Alcohol dehydrogenase (EC 1.1.1.1) |
| 5 | PGF_10049811 | Protein translocase subunit SecA |
| 6 | PGF_07830674 | Alanyl-tRNA synthetase (EC 6.1.1.7) |
| 7 | PGF_00031036 | Periplasmic nitrate reductase (EC 1.7.99.4) |
| 8 | PGF_04438983 | ATP-dependent protease La (EC 3.4.21.53) Type I |
| 9 | PGF_00008595 | Glycerol-3-phosphate acyltransferase (EC 2.3.1.15) |
| 10 | PGF_02906681 | Transcription accessory protein (S1 RNA-binding domain) |
| 11 | PGF_10471233 | ATP-dependent DNA helicase UvrD/PcrA (EC 3.6.4.12) |
| 12 | PGF_01219411 | 5-methyltetrahydropteroyltriglutamate--homocysteine methyltransferase (EC 2.1.1.14) |
| 13 | PGF_06915428 | ATP-dependent DNA helicase Rep |
| 14 | PGF_00950554 | Excinuclease ABC subunit B |
| 15 | PGF_01175575 | Threonyl-tRNA synthetase (EC 6.1.1.3) |
| 16 | PGF_00903690 | Exoribonuclease II (EC 3.1.13.1) |
| 17 | PGF_06856090 | Uncharacterized transporter YfbS |
| 18 | PGF_00426115 | 1-deoxy-D-xylulose 5-phosphate synthase (EC 2.2.1.7) |
| 19 | PGF_05421852 | Chaperone protein HtpG |
| 20 | PGF_05949521 | Protein translocase subunit SecD |
| 21 | PGF_00423472 | DinG family ATP-dependent helicase YoaA |
| 22 | PGF_00060414 | Translation elongation factor LepA |
| 23 | PGF_08442788 | Peptidyl-prolyl cis-trans isomerase PpiD (EC 5.2.1.8) |
| 24 | PGF_05507938 | DNA mismatch repair protein MutL |
| 25 | PGF_00926109 | Arginyl-tRNA synthetase (EC 6.1.1.19) |
| 26 | PGF_00421098 | DEAD-box ATP-dependent RNA helicase DeaD (= CshA) (EC 3.6.4.13) |
| 27 | PGF_00055324 | Anaerobic glycerol-3-phosphate dehydrogenase subunit A (EC 1.1.5.3) |
| 28 | PGF_01212739 | Heat shock protein 60 kDa family chaperone GroEL |
| 29 | PGF_00008326 | Glutaminyl-tRNA synthetase (EC 6.1.1.18) |
| 30 | PGF_00014144 | Inner membrane protein YejM, alkaline phosphatase superfamily |
| 31 | PGF_07058357 | Single-stranded-DNA-specific exonuclease RecJ |
| 32 | PGF_00421679 | DNA primase DnaG |
| 33 | PGF_08652177 | Cytochrome c-type biogenesis protein DsbD, protein-disulfide reductase (EC 1.8.1.8) |
| 34 | PGF_01569608 | Putative transport protein YidE |
| 35 | PGF_00416129 | CTP synthase (EC 6.3.4.2) |
| 36 | PGF_03751973 | Inner membrane protein translocase and chaperone YidC, long form |
| 37 | PGF_00006935 | GMP synthase [glutamine-hydrolyzing], amidotransferase subunit (EC 6.3.5.2) / GMP synthase [glutamine-hydrolyzing], ATP pyrophosphatase subunit (EC 6.3.5.2) |
| 38 | PGF_00013509 | IMP cyclohydrolase (EC 3.5.4.10) / Phosphoribosylaminoimidazolecarboxamide formyltransferase (EC 2.1.2.3) |
| 39 | PGF_04569524 | ATP synthase alpha chain (EC 3.6.3.14) |
| 40 | PGF_00025290 | Na+/H+ antiporter NhaB |
| 41 | PGF_01605461 | Cytochrome d ubiquinol oxidase subunit I (EC 1.10.3.-) |
| 42 | PGF_04602135 | Lipid A phosphoethanolamine transferase EptA/PmrC (EC 2.7.8.43) |
| 43 | PGF_00023745 | N-acetylgalactosamine 6-sulfate sulfatase (GALNS) |
| 44 | PGF_00015971 | L-2,4-diaminobutyrate decarboxylase (EC 4.1.1.86) |
| 45 | PGF_00710545 | Lysyl-tRNA synthetase (class II) (EC 6.1.1.6) |
| 46 | PGF_00050995 | Amidophosphoribosyltransferase (EC 2.4.2.14) |
| 47 | PGF_00015855 | Ketol-acid reductoisomerase (NADP(+)) (EC 1.1.1.86) |
| 48 | PGF_06027248 | Glucose-6-phosphate 1-dehydrogenase (EC 1.1.1.49) |
| 49 | PGF_00425024 | Exodeoxyribonuclease VII large subunit (EC 3.1.11.6) |
| 50 | PGF_10048015 | Inosine-5'-monophosphate dehydrogenase (EC 1.1.1.205) / CBS domain |
| 51 | PGF_00024322 | NAD(P) transhydrogenase subunit beta (EC 1.6.1.2) |
| 52 | PGF_06835522 | Dihydrolipoamide dehydrogenase of pyruvate dehydrogenase complex (EC 1.8.1.4) @ Dihydrolipoamide dehydrogenase (EC 1.8.1.4) |
| 53 | PGF_07760799 | Pyruvate kinase (EC 2.7.1.40) |
| 54 | PGF_08788152 | Trk potassium uptake system protein TrkH |
| 55 | PGF_00275905 | Glutamine synthetase type I (EC 6.3.1.2) |
| 56 | PGF_00413237 | tRNA 4-thiouridine synthase (EC 2.8.1.4) / Rhodanese-like domain required for thiamine synthesis |
| 57 | PGF_00013100 | D-glycero-beta-D-manno-heptose 1-phosphate adenylyltransferase (EC 2.7.7.70) / D-glycero-beta-D-manno-heptose-7-phosphate kinase (EC 2.7.1.167) |
| 58 | PGF_00066502 | Asparaginyl-tRNA synthetase (EC 6.1.1.22) |
| 59 | PGF_00006461 | Fumarate hydratase class II (EC 4.2.1.2) |
| 60 | PGF_07096769 | UDP-N-acetylmuramoyl-dipeptide--2,6-diaminopimelate ligase (EC 6.3.2.13) |
| 61 | PGF_05195027 | ATP synthase beta chain (EC 3.6.3.14) |
| 62 | PGF_10482116 | Cardiolipin synthase, bacterial type ClsA |
| 63 | PGF_02105064 | Uncharacterized protease YegQ |
| 64 | PGF_00064004 | UDP-N-acetylmuramate:L-alanyl-gamma-D-glutamyl-meso-diaminopimelate ligase (EC 6.3.2.-) |
| 65 | PGF_04208139 | Phosphomannomutase (EC 5.4.2.8) |
| 66 | PGF_06627798 | Trk potassium uptake system protein TrkA |
| 67 | PGF_00423084 | Diaminobutyrate--2-oxoglutarate aminotransferase (EC 2.6.1.76) |
| 68 | PGF_07540743 | Thiamin ABC transporter, transmembrane component |
| 69 | PGF_01137124 | Phosphoglucosamine mutase (EC 5.4.2.10) |
| 70 | PGF_04521913 | UDP-N-acetylmuramoyl-tripeptide--D-alanyl-D-alanine ligase (EC 6.3.2.10) |
| 71 | PGF_08454293 | N-acetylglucosamine-1-phosphate uridyltransferase (EC 2.7.7.23) / Glucosamine-1-phosphate N-acetyltransferase (EC 2.3.1.157) |
| 72 | PGF_00956915 | O-succinylbenzoic acid--CoA ligase (EC 6.2.1.26) |
| 73 | PGF_00417961 | Chromosome partition protein MukF |
| 74 | PGF_00025184 | Na(+)-translocating NADH-quinone reductase subunit A (EC 1.6.5.8) |
| 75 | PGF_02209787 | Conserved protein YcjX with nucleoside triphosphate hydrolase domain |
| 76 | PGF_06935032 | Adenylosuccinate synthetase (EC 6.3.4.4) |
| 77 | PGF_03752718 | Intramembrane protease RasP/YluC, implicated in cell division based on FtsL cleavage |
| 78 | PGF_06982509 | Efflux transport system, outer membrane factor (OMF) lipoprotein |
| 79 | PGF_02452671 | Cell division trigger factor (EC 5.2.1.8) |
| 80 | PGF_03117340 | Peptidase B (EC 3.4.11.23) |
| 81 | PGF_05622318 | Serine hydroxymethyltransferase (EC 2.1.2.1) |
| 82 | PGF_02862285 | Argininosuccinate synthase (EC 6.3.4.5) |
| 83 | PGF_00064931 | Uncharacterized protein EC-HemY in Proteobacteria (unrelated to HemY-type PPO in GramPositives) |
| 84 | PGF_00008334 | Glutamyl-tRNA reductase (EC 1.2.1.70) |
| 85 | PGF_07868088 | ATP-dependent RNA helicase SrmB |
| 86 | PGF_02417803 | D-tagatose-1,6-bisphosphate aldolase subunit KbaZ |
| 87 | PGF_06162930 | UDP-N-acetylmuramoyl-L-alanine--D-glutamate ligase (EC 6.3.2.9) |
| 88 | PGF_03316046 | Threonine synthase (EC 4.2.3.1) |
| 89 | PGF_00843497 | Cell division protein FtsA |
| 90 | PGF_07844318 | 3-phosphoshikimate 1-carboxyvinyltransferase (EC 2.5.1.19) |
| 91 | PGF_07750515 | Phosphoribosylamine--glycine ligase (EC 6.3.4.13) |
| 92 | PGF_05760069 | Histidinol dehydrogenase (EC 1.1.1.23) |
| 93 | PGF_04009223 | Uncharacterized protein EC-HemX |
| 94 | PGF_00033904 | Phosphopentomutase (EC 5.4.2.7) |
| 95 | PGF_00769755 | 16S rRNA (cytosine(967)-C(5))-methyltransferase (EC 2.1.1.176) |
| 96 | PGF_03080445 | Anaerobic glycerol-3-phosphate dehydrogenase subunit B (EC 1.1.5.3) |
| 97 | PGF_04485073 | Dihydrofolate synthase (EC 6.3.2.12) @ Folylpolyglutamate synthase (EC 6.3.2.17) |
| 98 | PGF_00037171 | Probable low-affinity inorganic phosphate transporter |
| 99 | PGF_09258296 | Fe2+ ABC transporter, substrate binding protein |
| 100 | PGF_00421013 | 3-oxoacyl-[acyl-carrier-protein] synthase, KASI (EC 2.3.1.41) |
| 101 | PGF_08177814 | Lipoprotein releasing system transmembrane protein LolE |
| 102 | PGF_06747785 | AmpG permease |
| 103 | PGF_00011634 | HflK protein |
| 104 | PGF_06606669 | Alanine transaminase (EC 2.6.1.2) |
| 105 | PGF_09945671 | Acetate kinase (EC 2.7.2.1) |
| 106 | PGF_09111052 | Phosphopantothenoylcysteine decarboxylase (EC 4.1.1.36) / Phosphopantothenoylcysteine synthetase (EC 6.3.2.5) |
| 107 | PGF_02945982 | CzcABC family efflux RND transporter, membrane fusion protein |
| 108 | PGF_00016338 | ATP-dependent Clp protease ATP-binding subunit ClpX |
| 109 | PGF_09188652 | Molybdopterin molybdenumtransferase (EC 2.10.1.1) |
| 110 | PGF_03520151 | Cell division protein FtsZ |
| 111 | PGF_02029783 | GTP-binding protein Obg |
| 112 | PGF_02877184 | Chorismate mutase I (EC 5.4.99.5) / Prephenate dehydratase (EC 4.2.1.51) |
| 113 | PGF_04460416 | Tryptophan-specific transport protein |
| 114 | PGF_10125426 | Peptidoglycan glycosyltransferase FtsW (EC 2.4.1.129) |
| 115 | PGF_02908669 | N-acetylgalactosamine-6-phosphate deacetylase |
| 116 | PGF_00054528 | Succinyl-CoA ligase [ADP-forming] beta chain (EC 6.2.1.5) |
| 117 | PGF_05292270 | Ribonucleotide reductase of class Ia (aerobic), beta subunit (EC 1.17.4.1) |
| 118 | PGF_02061452 | Queuine tRNA-ribosyltransferase (EC 2.4.2.29) |
| 119 | PGF_10109529 | Cytochrome d ubiquinol oxidase subunit II (EC 1.10.3.-) |
| 120 | PGF_03006190 | 2-polyprenyl-6-methoxyphenol hydroxylase |
| 121 | PGF_00580333 | Lipopolysaccharide assembly protein LapB |
| 122 | PGF_07800768 | 2-polyprenylphenol hydroxylase |
| 123 | PGF_00420053 | Cysteine desulfurase (EC 2.8.1.7) => sulfur transfer pathway protein CsdA |
| 124 | PGF_01126005 | Rod shape-determining protein RodA |
| 125 | PGF_00024234 | N-succinyl-L,L-diaminopimelate desuccinylase (EC 3.5.1.18) |
| 126 | PGF_00417831 | Chorismate mutase I (EC 5.4.99.5) / Cyclohexadienyl dehydrogenase (EC 1.3.1.12)(EC 1.3.1.43) |
| 127 | PGF_07304022 | tRNA-specific 2-thiouridylase MnmA (EC 2.8.1.13) |
| 128 | PGF_10143857 | Fructose-bisphosphate aldolase class II (EC 4.1.2.13) |
| 129 | PGF_00004214 | Fe2+ ABC transporter, permease protein 2 |
| 130 | PGF_01985478 | Biosynthetic Aromatic amino acid aminotransferase beta (EC 2.6.1.57) |
| 131 | PGF_00007012 | GTP-binding and nucleic acid-binding protein YchF |
| 132 | PGF_00067739 | [4Fe-4S] cluster assembly scaffold protein Mrp (=ApbC) |
| 133 | PGF_00033359 | Phospho-N-acetylmuramoyl-pentapeptide-transferase (EC 2.7.8.13) |
| 134 | PGF_08196377 | Spermidine/putrescine import ABC transporter ATP-binding protein PotA (TC 3.A.1.11.1) |
| 135 | PGF_07015581 | Carbamoyl-phosphate synthase small chain (EC 6.3.5.5) |
| 136 | PGF_06473395 | DNA polymerase III beta subunit (EC 2.7.7.7) |
| 137 | PGF_00034566 | Poly(A) polymerase (EC 2.7.7.19) |
| 138 | PGF_01466819 | N-acetylmuramoyl-L-alanine amidase (EC 3.5.1.28) |
| 139 | PGF_02959749 | Ribonuclease D (EC 3.1.26.3) |
| 140 | PGF_10387199 | DNA recombination and repair protein RecF |
| 141 | PGF_00020717 | Methionine ABC transporter ATP-binding protein |
| 142 | PGF_03116630 | Membrane-bound lytic murein transglycosylase B |
| 143 | PGF_00030694 | Peptide ABC transporter, ATP-binding protein SapD |
| 144 | PGF_07191648 | 3-isopropylmalate dehydrogenase (EC 1.1.1.85) |
| 145 | PGF_00033237 | Phosphate starvation-inducible protein PhoH, predicted ATPase |
| 146 | PGF_08461341 | Lipopolysaccharide export system permease protein LptF |
| 147 | PGF_10439576 | FAD:protein FMN transferase (EC 2.7.1.180) @ FAD:protein FMN transferase (EC 2.7.1.180), NqrBC-associated |
| 148 | PGF_03295331 | UDP-N-acetylglucosamine--N-acetylmuramyl-(pentapeptide) pyrophosphoryl-undecaprenol N-acetylglucosamine transferase (EC 2.4.1.227) |
| 149 | PGF_00033954 | Phosphoribosylformylglycinamidine cyclo-ligase (EC 6.3.3.1) |
| 150 | PGF_00062045 | Tryptophanyl-tRNA synthetase (EC 6.1.1.2) |
| 151 | PGF_08376928 | Branched-chain amino acid aminotransferase (EC 2.6.1.42) |
| 152 | PGF_00024274 | N5-carboxyaminoimidazole ribonucleotide synthase (EC 6.3.4.18) |
| 153 | PGF_00026349 | Nucleoid-associated protein NdpA |
| 154 | PGF_00033289 | Phosphate:acyl-ACP acyltransferase PlsX (EC 2.3.1.n2) |
| 155 | PGF_05406959 | beta-N-acetylglucosaminidase (EC 3.2.1.52) |
| 156 | PGF_02019462 | Phenylalanyl-tRNA synthetase alpha chain (EC 6.1.1.20) |
| 157 | PGF_00780840 | Undecaprenyl-phosphate alpha-N-acetylglucosaminyl 1-phosphate transferase (EC 2.7.8.33) |
| 158 | PGF_00006380 | Fructose-1,6-bisphosphatase, type I (EC 3.1.3.11) |
| 159 | PGF_07313224 | Outer membrane stress sensor protease DegS |
| 160 | PGF_01534078 | Murein endolytic transglycosylase MltG |
| 161 | PGF_02191019 | Thiamine-monophosphate kinase (EC 2.7.4.16) |
| 162 | PGF_00648450 | Rod shape-determining protein MreB |
| 163 | PGF_03811905 | Histidinol-phosphate aminotransferase (EC 2.6.1.9) |
| 164 | PGF_00067160 | Aspartate--ammonia ligase (EC 6.3.1.1) |
| 165 | PGF_10244701 | GTP 3',8-cyclase (EC 4.1.99.22) |
| 166 | PGF_07421229 | 6-phosphofructokinase (EC 2.7.1.11) |
| 167 | PGF_09201380 | DNA polymerase III delta subunit (EC 2.7.7.7) |
| 168 | PGF_03374975 | O-succinylbenzoate synthase (EC 4.2.1.113) |
| 169 | PGF_00048928 | Ribosome small subunit biogenesis RbfA-release protein RsgA |
| 170 | PGF_00024478 | NAD-dependent glyceraldehyde-3-phosphate dehydrogenase (EC 1.2.1.12) |
| 171 | PGF_02390924 | 16S rRNA (cytosine(1402)-N(4))-methyltransferase (EC 2.1.1.199) |
| 172 | PGF_03589438 | Protein translocase subunit SecF |
| 173 | PGF_00048829 | LSU rRNA pseudouridine(2605) synthase (EC 5.4.99.22) |
| 174 | PGF_01625234 | Thioredoxin reductase (EC 1.8.1.9) |
| 175 | PGF_06946342 | tRNA-dihydrouridine(20/20a) synthase (EC 1.3.1.91) |
| 176 | PGF_08244241 | Tetraacyldisaccharide 4'-kinase (EC 2.7.1.130) |
| 177 | PGF_00063944 | UDP-3-O-[3-hydroxymyristoyl] N-acetylglucosamine deacetylase (EC 3.5.1.108) |
| 178 | PGF_00709681 | Inner membrane protein YrbG, predicted calcium/sodium:proton antiporter |
| 179 | PGF_00017063 | Lipid A biosynthesis myristoyltransferase (EC 2.3.1.243) |
| 180 | PGF_00018952 | Malate dehydrogenase (EC 1.1.1.37) |
| 181 | PGF_06755829 | DNA polymerase III delta prime subunit (EC 2.7.7.7) |
| 182 | PGF_00420953 | 3-oxoacyl-[acyl-carrier-protein] synthase, KASIII (EC 2.3.1.180) |
| 183 | PGF_00055985 | Tagatose 1,6-bisphosphate aldolase (EC 4.1.2.40) |
| 184 | PGF_00013017 | ADP-L-glycero-D-manno-heptose-6-epimerase (EC 5.1.3.20) |
| 185 | PGF_02996782 | LSU rRNA pseudouridine(955/2504/2580) synthase (EC 5.4.99.24) |
| 186 | PGF_05165078 | Methionyl-tRNA formyltransferase (EC 2.1.2.9) |
| 187 | PGF_01528300 | Formate dehydrogenase O beta subunit (EC 1.2.1.2) |
| 188 | PGF_00017939 | Lysine 2,3-aminomutase (EC 5.4.3.2) |
| 189 | PGF_00052053 | Sigma factor RpoE negative regulatory protein RseB precursor |
| 190 | PGF_05863337 | Lysophospholipase L2 (EC 3.1.1.5) |
| 191 | PGF_00006376 | Fructose repressor FruR, LacI family |
| 192 | PGF_00057511 | Transcriptional activator MetR |
| 193 | PGF_04807486 | tRNA dimethylallyltransferase (EC 2.5.1.75) |
| 194 | PGF_00017281 | Lipopolysaccharide core heptosyltransferase I |
| 195 | PGF_00009967 | Glycyl-tRNA synthetase alpha chain (EC 6.1.1.14) |
| 196 | PGF_10518532 | D-alanine--D-alanine ligase (EC 6.3.2.4) |
| 197 | PGF_01763778 | Lipid A biosynthesis lauroyl acyltransferase (EC 2.3.1.241) |
| 198 | PGF_00026453 | Acetyl-coenzyme A carboxyl transferase beta chain (EC 6.4.1.2) |
| 199 | PGF_00069596 | Ferrous-iron efflux pump FieF |
| 200 | PGF_02382389 | Ribokinase (EC 2.7.1.15) |
| 201 | PGF_00048858 | Ribosomal protein S6--L-glutamate ligase |
| 202 | PGF_10525969 | Magnesium and cobalt efflux protein CorC |
| 203 | PGF_05873936 | Hydrogen peroxide-inducible genes activator => OxyR |
| 204 | PGF_00549380 | 4-hydroxy-tetrahydrodipicolinate synthase (EC 4.3.3.7) |
| 205 | PGF_01809653 | Uncharacterized inner membrane transporter YhbE |
| 206 | PGF_03839583 | Lysine exporter LysO |
| 207 | PGF_03011092 | ATP phosphoribosyltransferase (EC 2.4.2.17) => HisGl |
| 208 | PGF_00052943 | Site-specific tyrosine recombinase XerD |
| 209 | PGF_00926610 | Glycine cleavage system transcriptional activator GcvA |
| 210 | PGF_00023919 | N-acetylneuraminate lyase (EC 4.1.3.3) |
| 211 | PGF_00413295 | tRNA pseudouridine(55) synthase (EC 5.4.99.25) |
| 212 | PGF_00019974 | Membrane protein YcjF |
| 213 | PGF_00053700 | Spermidine/putrescine import ABC transporter permease protein PotB (TC 3.A.1.11.1) |
| 214 | PGF_03938996 | Phosphatidylserine decarboxylase (EC 4.1.1.65) |
| 215 | PGF_10462808 | 33 kDa chaperonin HslO |
| 216 | PGF_00853393 | Succinyl-CoA ligase [ADP-forming] alpha chain (EC 6.2.1.5) |
| 217 | PGF_06068352 | Cytochrome c heme lyase subunit CcmH |
| 218 | PGF_04486278 | Biotin operon repressor / Biotin--protein ligase (EC 6.3.4.9)(EC 6.3.4.10)(EC 6.3.4.11)(EC 6.3.4.15) |
| 219 | PGF_00010729 | Transcriptional regulator IlvY, LysR family |
| 220 | PGF_00423709 | DnaJ-like protein DjlA |
| 221 | PGF_00048842 | Ribosomal protein L3 N(5)-glutamine methyltransferase (EC 2.1.1.298) |
| 222 | PGF_07102049 | UPF0701 protein YicC |
| 223 | PGF_10363383 | Murein hydrolase activator NlpD |
| 224 | PGF_00033936 | Phosphoribosylaminoimidazole-succinocarboxamide synthase (EC 6.3.2.6) |
| 225 | PGF_00020984 | Methenyltetrahydrofolate cyclohydrolase (EC 3.5.4.9) / Methylenetetrahydrofolate dehydrogenase (NADP+) (EC 1.5.1.5) |
| 226 | PGF_00048840 | Ribosomal protein L11 methyltransferase |
| 227 | PGF_05091456 | Uncharacterized inner membrane protein RarD |
| 228 | PGF_10374939 | Polyferredoxin NapH (periplasmic nitrate reductase) |
| 229 | PGF_03846068 | Pyridoxal kinase (EC 2.7.1.35) |
| 230 | PGF_00030246 | Penicillin-insensitive murein endopeptidase |
| 231 | PGF_05930156 | Inner membrane protein YihY, formerly thought to be RNase BN |
| 232 | PGF_00016393 | LSU ribosomal protein L2p (L8e) |
| 233 | PGF_00023828 | N-acetylmannosamine kinase (EC 2.7.1.60) |
| 234 | PGF_00020172 | 2,3,4,5-tetrahydropyridine-2,6-dicarboxylate N-succinyltransferase (EC 2.3.1.117) |
| 235 | PGF_00423533 | 4-diphosphocytidyl-2-C-methyl-D-erythritol kinase (EC 2.7.1.148) |
| 236 | PGF_00876943 | 1,4-dihydroxy-2-naphthoate polyprenyltransferase (EC 2.5.1.74) |
| 237 | PGF_05580933 | Peptide chain release factor N(5)-glutamine methyltransferase (EC 2.1.1.297) |
| 238 | PGF_00006320 | Formyltetrahydrofolate deformylase (EC 3.5.1.10) |
| 239 | PGF_05362726 | Formate dehydrogenase formation protein FdhE |
| 240 | PGF_03427698 | 3',5'-cyclic-nucleotide phosphodiesterase (EC 3.1.4.17) |
| 241 | PGF_00065941 | Universal stress protein E |
| 242 | PGF_00127024 | Cell division inhibitor Slr1223 (YfcH in EC), contains epimerase/dehydratase and DUF1731 domains |
| 243 | PGF_00418497 | Cof protein, HD superfamily hydrolase |
| 244 | PGF_07420523 | Exodeoxyribonuclease III (EC 3.1.11.2) |
| 245 | PGF_10160552 | Enoyl-[acyl-carrier-protein] reductase [NADH] (EC 1.3.1.9) |
| 246 | PGF_06629251 | Methionine aminopeptidase (EC 3.4.11.18) |
| 247 | PGF_00033729 | Acyl-[acyl-carrier-protein]--UDP-N-acetylglucosamine O-acyltransferase (EC 2.3.1.129) |
| 248 | PGF_00049827 | SSU rRNA (adenine(1518)-N(6)/adenine(1519)-N(6))-dimethyltransferase (EC 2.1.1.182) |
| 249 | PGF_05147941 | Nucleoside-diphosphate-sugar epimerases |
| 250 | PGF_00024855 | NADPH-dependent 7-cyano-7-deazaguanine reductase (EC 1.7.1.13) |
| 251 | PGF_00058785 | Transcriptional regulator of glmS gene, DeoR family |
| 252 | PGF_04150742 | Zinc ABC transporter, permease protein ZnuB |
| 253 | PGF_09950480 | Aldose 1-epimerase |
| 254 | PGF_03034557 | Fumarate reductase iron-sulfur protein (EC 1.3.5.4) |
| 255 | PGF_00058575 | Transcriptional regulator KdgR, KDG operon repressor |
| 256 | PGF_07609122 | Pyrroline-5-carboxylate reductase (EC 1.5.1.2) |
| 257 | PGF_00026615 | N-acetylglutamate kinase (EC 2.7.2.8) |
| 258 | PGF_07763915 | Bis(5'-nucleosyl)-tetraphosphatase, symmetrical (EC 3.6.1.41) |
| 259 | PGF_04127376 | Twin-arginine translocation protein TatC |
| 260 | PGF_00912542 | L-cystine ABC transporter, substrate-binding protein TcyA |
| 261 | PGF_05048110 | Spermidine/putrescine import ABC transporter permease protein PotC (TC 3.A.1.11.1) |
| 262 | PGF_02011760 | Imidazole glycerol phosphate synthase cyclase subunit |
| 263 | PGF_00064270 | UPF0246 protein YaaA |
| 264 | PGF_00418128 | 3-deoxy-manno-octulosonate cytidylyltransferase (EC 2.7.7.38) |
| 265 | PGF_02472178 | Uncharacterized metal-dependent hydrolase YcfH |
| 266 | PGF_08518355 | ATP synthase F0 sector subunit a (EC 3.6.3.14) |
| 267 | PGF_05740384 | Formamidopyrimidine-DNA glycosylase (EC 3.2.2.23) |
| 268 | PGF_03863088 | FIG000875: Thioredoxin domain-containing protein EC-YbbN |
| 269 | PGF_00067732 | Zn-ribbon-containing, possibly nucleic-acid-binding protein |
| 270 | PGF_10506571 | DNA polymerase III epsilon subunit (EC 2.7.7.7) |
| 271 | PGF_01110486 | Flavodoxin reductases (ferredoxin-NADPH reductases) family 1 |
| 272 | PGF_07502400 | Transcriptional repressor of aga operon |
| 273 | PGF_04505269 | SSU ribosomal protein S2p (SAe) |
| 274 | PGF_08789310 | L-cystine ABC transporter, ATP-binding protein TcyC |
| 275 | PGF_00033946 | Phosphoribosylformimino-5-aminoimidazole carboxamide ribotide isomerase (EC 5.3.1.16) |
| 276 | PGF_08110668 | Glycerol-3-phosphate regulon repressor GlpR |
| 277 | PGF_00426810 | 5'-nucleotidase SurE (EC 3.1.3.5) @ Exopolyphosphatase (EC 3.6.1.11) |
| 278 | PGF_00401928 | 2-methoxy-6-polyprenyl-1,4-benzoquinol methylase (EC 2.1.1.201) @ Demethylmenaquinone methyltransferase (EC 2.1.1.163) |
| 279 | PGF_00051717 | NADP-dependent 3-hydroxy acid dehydrogenase YdfG (EC 1.1.1.381) @ 3-hydroxypropionate dehydrogenase (EC 1.1.1.298) |
| 280 | PGF_00581959 | Hybrid peroxiredoxin hyPrx5 (EC 1.11.1.15) |
| 281 | PGF_08693885 | 3-oxoacyl-[acyl-carrier protein] reductase (EC 1.1.1.100), FadG |
| 282 | PGF_00420482 | Cytochrome c-type biogenesis protein CcmC, putative heme lyase for CcmE |
| 283 | PGF_00017190 | Lipopolysaccharide ABC transporter, ATP-binding protein LptB |
| 284 | PGF_02923127 | Uridylate kinase (EC 2.7.4.22) |
| 285 | PGF_05867827 | Molybdopterin-synthase adenylyltransferase (EC 2.7.7.80) |
| 286 | PGF_00038987 | Purine nucleoside phosphorylase (EC 2.4.2.1) |
| 287 | PGF_10535492 | Cell division protein FtsQ |
| 288 | PGF_00049542 | SAM-dependent methyltransferase HI0095 (UbiE paralog) |
| 289 | PGF_00502921 | Arginine ABC transporter, substrate-binding protein ArtI |
| 290 | PGF_00049889 | SSU ribosomal protein S3p (S3e) |
| 291 | PGF_07631588 | Acyl-CoA:1-acyl-sn-glycerol-3-phosphate acyltransferase (EC 2.3.1.51) @ Acyl-ACP:1-acyl-sn-glycerol-3-phosphate acyltransferase (EC 2.3.1.n4) |
| 292 | PGF_05572422 | Molybdenum ABC transporter, substrate-binding protein ModA |
| 293 | PGF_01052212 | Uroporphyrinogen-III synthase (EC 4.2.1.75) |
| 294 | PGF_00036567 | Aerobic respiration control response regulator ArcA |
| 295 | PGF_00037301 | Probable ribonuclease HI0526 precursor |
| 296 | PGF_00006144 | Formate dehydrogenase O gamma subunit (EC 1.2.1.2) |
| 297 | PGF_06250778 | Esterase ybfF (EC 3.1.-.-) |
| 298 | PGF_00417959 | Chromosome partition protein MukE |
| 299 | PGF_00777371 | Orotidine 5'-phosphate decarboxylase (EC 4.1.1.23) |
| 300 | PGF_00417755 | 3-demethylubiquinol 3-O-methyltransferase (EC 2.1.1.64) @ 2-polyprenyl-6-hydroxyphenyl methylase (EC 2.1.1.222) |
| 301 | PGF_10245672 | Ribulose-phosphate 3-epimerase (EC 5.1.3.1) |
| 302 | PGF_00648054 | Tol-Pal system protein TolQ |
| 303 | PGF_00906364 | Hydroxyacylglutathione hydrolase (EC 3.1.2.6) |
| 304 | PGF_10369163 | 4'-phosphopantetheinyl transferase (EC 2.7.8.-) |
| 305 | PGF_07925636 | UPF0126 inner membrane protein YicG |
| 306 | PGF_00064204 | Arginine ABC transporter, permease protein ArtM |
| 307 | PGF_03790040 | Ribonuclease III (EC 3.1.26.3) |
| 308 | PGF_10535258 | Lipoprotein-releasing system ATP-binding protein LolD |
| 309 | PGF_00066139 | Uracil-DNA glycosylase, family 1 (EC 3.2.2.27) |
| 310 | PGF_02230495 | N-acetylmannosamine-6-phosphate 2-epimerase (EC 5.1.3.9) |
| 311 | PGF_00294361 | hypothetical protein |
| 312 | PGF_06216244 | DNA recombination and repair protein RecO |
| 313 | PGF_00423055 | Diadenosine tetraphosphatase and related serine/threonine protein phosphatases |
| 314 | PGF_00014196 | Inner membrane protein YohK |
| 315 | PGF_00001005 | FIG009095: D,D-carboxypeptidase family protein |
| 316 | PGF_07071261 | GTP cyclohydrolase II (EC 3.5.4.25) |
| 317 | PGF_09626318 | SSU rRNA pseudouridine(516) synthase (EC 5.4.99.19) |
| 318 | PGF_02820109 | GTP cyclohydrolase I (EC 3.5.4.16) type 1 |
| 319 | PGF_00777996 | 5'-methylthioadenosine nucleosidase (EC 3.2.2.16) @ S-adenosylhomocysteine nucleosidase (EC 3.2.2.9) |
| 320 | PGF_00048824 | LSU rRNA pseudouridine(746) synthase (EC 5.4.99.29) @ tRNA pseudouridine(32) synthase (EC 5.4.99.28) |
| 321 | PGF_00063396 | Type II secretory pathway, component PulJ |
| 322 | PGF_04244475 | Adenylate kinase (EC 2.7.4.3) |
| 323 | PGF_00421408 | DNA mismatch repair endonuclease MutH |
| 324 | PGF_00403733 | predicted 4-deoxy-L-threo-5-hexosulose-uronate ketol-isomerase (EC 5.3.1.17) |
| 325 | PGF_08122432 | 2-C-methyl-D-erythritol 4-phosphate cytidylyltransferase (EC 2.7.7.60) |
| 326 | PGF_06842926 | Molybdenum ABC transporter ATP-binding protein ModC |
| 327 | PGF_04007946 | Octanoate-[acyl-carrier-protein]-protein-N-octanoyltransferase (EC 2.3.1.181) |
| 328 | PGF_00503328 | UPF0319 protein YccT precursor |
| 329 | PGF_00730641 | 4-hydroxy-2-oxoglutarate aldolase (EC 4.1.3.16) @ 2-dehydro-3-deoxyphosphogluconate aldolase (EC 4.1.2.14) |
| 330 | PGF_08394843 | Endonuclease III (EC 4.2.99.18) |
| 331 | PGF_05026985 | Deoxyribose-phosphate aldolase (EC 4.1.2.4) |
| 332 | PGF_06857975 | Cell-division-associated, ABC-transporter-like signaling protein FtsE |
| 333 | PGF_00049893 | SSU ribosomal protein S4p (S9e) @ SSU ribosomal protein S4p (S9e), zinc-independent |
| 334 | PGF_00063937 | UDP-2,3-diacylglucosamine diphosphatase (EC 3.6.1.54) |
| 335 | PGF_03810679 | SOS-response repressor and protease LexA (EC 3.4.21.88) |
| 336 | PGF_00473519 | Superoxide dismutase [Mn] (EC 1.15.1.1) |
| 337 | PGF_10381714 | Orotate phosphoribosyltransferase (EC 2.4.2.10) |
| 338 | PGF_00033312 | Phosphatidylglycerophosphatase B (EC 3.1.3.27) |
| 339 | PGF_00031127 | Periplasmic thiol:disulfide interchange protein DsbA |
| 340 | PGF_04565471 | ABC transporter involved in cytochrome c biogenesis, ATPase component CcmA |
| 341 | PGF_09749430 | SAM-dependent methyltransferase |
| 342 | PGF_04681578 | Alkyl hydroperoxide reductase subunit C-like protein |
| 343 | PGF_04512522 | LSU ribosomal protein L4p (L1e) |
| 344 | PGF_02463284 | Recombination protein RecR |
| 345 | PGF_02787494 | Phosphate transport regulator (distant homolog of PhoU) |
| 346 | PGF_09866709 | Outer membrane lipoprotein carrier protein LolA |
| 347 | PGF_00008838 | 16S rRNA (guanine(527)-N(7))-methyltransferase (EC 2.1.1.170) |
| 348 | PGF_06111020 | Thymidylate kinase (EC 2.7.4.9) |
| 349 | PGF_01323228 | Acyl-phosphate:glycerol-3-phosphate O-acyltransferase PlsY (EC 2.3.1.n3) |
| 350 | PGF_06660812 | 3-isopropylmalate dehydratase small subunit (EC 4.2.1.33) |
| 351 | PGF_00425812 | High frequency lysogenization protein HflD |
| 352 | PGF_10659937 | Phospholipid ABC transporter shuttle protein MlaC |
| 353 | PGF_00048556 | Ribonuclease HII (EC 3.1.26.4) |
| 354 | PGF_10504332 | Ubiquinone biosynthesis protein UbiJ |
| 355 | PGF_04760055 | D-sedoheptulose 7-phosphate isomerase (EC 5.3.1.28) |
| 356 | PGF_03751823 | Heat shock protein GrpE |
| 357 | PGF_00048589 | Ribonuclease T |
| 358 | PGF_01484108 | Electron transport complex protein RnfG |
| 359 | PGF_03333815 | Copper-sensing two-component system response regulator CpxR |
| 360 | PGF_05520131 | Hydroxymethylpyrimidine phosphate kinase ThiD (EC 2.7.4.7) |
| 361 | PGF_03079268 | RNA polymerase sigma factor RpoE |
| 362 | PGF_00743561 | Anthranilate synthase, amidotransferase component (EC 4.1.3.27) |
| 363 | PGF_00051143 | SeqA protein, negative modulator of initiation of replication |
| 364 | PGF_03799365 | Translation elongation factor P |
| 365 | PGF_00425679 | FIG000605: protein co-occurring with transport systems (COG1739) |
| 366 | PGF_00022507 | Molybdopterin adenylyltransferase (EC 2.7.7.75) |
| 367 | PGF_00049713 | SH3 domain protein |
| 368 | PGF_01810305 | Uncharacterized protein YjaG |
| 369 | PGF_10542033 | Electron transport complex protein RnfB |
| 370 | PGF_00026362 | Nucleoside 5-triphosphatase RdgB (dHAPTP, dITP, XTP-specific) (EC 3.6.1.66) |
| 371 | PGF_00660064 | Galactoside O-acetyltransferase (EC 2.3.1.18) |
| 372 | PGF_01785347 | Adenosine (5')-pentaphospho-(5'')-adenosine pyrophosphohydrolase |
| 373 | PGF_04039232 | Sigma factor RpoE negative regulatory protein RseA |
| 374 | PGF_00058666 | Transcriptional regulator SlmA, TetR family |
| 375 | PGF_00048926 | Ribosome recycling factor |
| 376 | PGF_03174068 | Transcription antitermination protein NusG |
| 377 | PGF_00598374 | Para-aminobenzoate synthase, amidotransferase component (EC 2.6.1.85) |
| 378 | PGF_00706032 | FIG139438: lipoprotein B |
| 379 | PGF_00424141 | Electron transport complex protein RnfE |
| 380 | PGF_03081665 | Cell division integral membrane protein, YggT and half-length relatives |
| 381 | PGF_00416576 | 3'-to-5' oligoribonuclease (orn) |
| 382 | PGF_02658061 | Phosphoribosyl-AMP cyclohydrolase (EC 3.5.4.19) / Phosphoribosyl-ATP pyrophosphatase (EC 3.6.1.31) |
| 383 | PGF_07391058 | D-glycero-beta-D-manno-heptose-1,7-bisphosphate 7-phosphatase (EC 3.1.3.82) |
| 384 | PGF_12869170 | Periplasmic chaperone of outer membrane proteins Skp @ Outer membrane protein H precursor |
| 385 | PGF_00042848 | Anaerobic respiratory reductase chaperone |
| 386 | PGF_00038329 | Der GTPase-activating protein YihI |
| 387 | PGF_00016444 | LSU ribosomal protein L6p (L9e) |
| 388 | PGF_06274006 | ATP synthase delta chain (EC 3.6.3.14) |
| 389 | PGF_00036688 | RNA chaperone ProQ |
| 390 | PGF_05357708 | DNA-3-methyladenine glycosylase (EC 3.2.2.20) |
| 391 | PGF_06894101 | 3-deoxy-D-manno-octulosonate 8-phosphate phosphatase (EC 3.1.3.45) |
| 392 | PGF_00019428 | ATP-dependent protease subunit HslV (EC 3.4.25.2) |
| 393 | PGF_03295678 | CDP-diacylglycerol--glycerol-3-phosphate 3-phosphatidyltransferase (EC 2.7.8.5) |
| 394 | PGF_09190895 | Aminodeoxychorismate lyase (EC 4.1.3.38) |
| 395 | PGF_00017327 | Lipopolysaccharide export system protein LptC |
| 396 | PGF_00000172 | FIG00696143: hypothetical protein |
| 397 | PGF_01922063 | Inorganic pyrophosphatase (EC 3.6.1.1) |
| 398 | PGF_02918324 | hypothetical protein |
| 399 | PGF_00039503 | Putative NAD(P)H nitroreductase YdjA |
| 400 | PGF_02918910 | FIG001587: exported protein |
| 401 | PGF_07549903 | Periplasmic thiol:disulfide oxidoreductase DsbB, required for DsbA reoxidation |
| 402 | PGF_02031353 | FIG01269488: protein, clustered with ribosomal protein L32p |
| 403 | PGF_04867467 | Imidazole glycerol phosphate synthase amidotransferase subunit HisH |
| 404 | PGF_04630848 | Cytochrome c-type biogenesis protein CcmE, heme chaperone |
| 405 | PGF_00049896 | SSU ribosomal protein S5p (S2e) |
| 406 | PGF_00060422 | Translation elongation factor P Lys34 hydroxylase |
| 407 | PGF_01416568 | LemA family protein |
| 408 | PGF_10499347 | Molybdopterin-guanine dinucleotide biosynthesis protein MobB |
| 409 | PGF_01213071 | LSU ribosomal protein L10p (P0) |
| 410 | PGF_00034972 | Adenine phosphoribosyltransferase (EC 2.4.2.7) |
| 411 | PGF_02386652 | Ribonuclease E inhibitor RraA |
| 412 | PGF_00016305 | LPS-assembly lipoprotein LptE |
| 413 | PGF_00808195 | DNA protection during starvation protein |
| 414 | PGF_00418655 | Colicin V production protein |
| 415 | PGF_04315180 | Protein-export protein SecB (maintains pre-export unfolded state) |
| 416 | PGF_00419913 | Cyclic pyranopterin monophosphate synthase (EC 4.6.1.17) |
| 417 | PGF_00049904 | SSU ribosomal protein S7p (S5e) |
| 418 | PGF_10546429 | ATP synthase F0 sector subunit b (EC 3.6.3.14) |
| 419 | PGF_05172785 | N5-carboxyaminoimidazole ribonucleotide mutase (EC 5.4.99.18) |
| 420 | PGF_07480521 | 23S rRNA (pseudouridine(1915)-N(3))-methyltransferase (EC 2.1.1.177) |
| 421 | PGF_01309240 | PTS system, glucose-specific IIA component (EC 2.7.1.199) |
| 422 | PGF_00057435 | Transcription elongation factor GreB |
| 423 | PGF_02328856 | Type IV pilus biogenesis protein PilF |
| 424 | PGF_01420802 | Transcriptional regulator MraZ |
| 425 | PGF_00033486 | Acyl-CoA thioesterase YciA, involved in membrane biogenesis |
| 426 | PGF_02881204 | Cytochrome c heme lyase subunit CcmL |
| 427 | PGF_00047274 | Transcriptional regulator AsnC |
| 428 | PGF_07504595 | 3-hydroxyacyl-[acyl-carrier-protein] dehydratase, FabZ form (EC 4.2.1.59) |
| 429 | PGF_06458877 | Phosphatidylglycerophosphatase A (EC 3.1.3.27) |
| 430 | PGF_00035243 | Possible periplasmic thiredoxin |
| 431 | PGF_00016452 | LSU ribosomal protein L9p |
| 432 | PGF_09348764 | Dihydrofolate reductase (EC 1.5.1.3) |
| 433 | PGF_00038642 | Protein SprT |
| 434 | PGF_00417577 | 3-dehydroquinate dehydratase II (EC 4.2.1.10) |
| 435 | PGF_02728237 | Putative sugar isomerase involved in processing of exogenous sialic acid |
| 436 | PGF_00040536 | Putative dehydrogenase |
| 437 | PGF_00023247 | Uncharacterized protein HI_0449 |
| 438 | PGF_04762552 | Phosphopantetheine adenylyltransferase (EC 2.7.7.3) |
| 439 | PGF_00413554 | tmRNA-binding protein SmpB |
| 440 | PGF_00048643 | Ribonucleotide reductase transcriptional regulator NrdR |
| 441 | PGF_01682834 | Bacterial ribosome SSU maturation protein RimP |
| 442 | PGF_00020361 | Metal-dependent hydrolase YbeY, involved in rRNA and/or ribosome maturation and assembly |
| 443 | PGF_00046597 | RNA polymerase-binding transcription factor DksA |
| 444 | PGF_00648021 | Biotin carboxyl carrier protein of acetyl-CoA carboxylase |
| 445 | PGF_00022550 | Molybdopterin synthase catalytic subunit MoaE (EC 2.8.1.12) |
| 446 | PGF_00004511 | Ferric siderophore transport system, biopolymer transport protein ExbB |
| 447 | PGF_00016342 | LSU ribosomal protein L15p (L27Ae) |
| 448 | PGF_00057028 | Tol-Pal system peptidoglycan-associated lipoprotein PAL |
| 449 | PGF_00426932 | 6,7-dimethyl-8-ribityllumazine synthase (EC 2.5.1.78) |
| 450 | PGF_00012126 | Histone acetyltransferase HPA2 and related acetyltransferases |
| 451 | PGF_00645831 | DNA polymerase III chi subunit (EC 2.7.7.7) |
| 452 | PGF_00025698 | Nitrate reductase cytochrome c550-type subunit |
| 453 | PGF_05586373 | Rhodanese-related sulfurtransferase YibN |
| 454 | PGF_00020996 | Methylglyoxal synthase (EC 4.2.3.3) |
| 455 | PGF_00016343 | LSU ribosomal protein L16p (L10e) |
| 456 | PGF_02156631 | Arginine pathway regulatory protein ArgR, repressor of arg regulon |
| 457 | PGF_00064239 | UPF0208 membrane protein YfbV |
| 458 | PGF_00057016 | Tol biopolymer transport system, TolR protein |
| 459 | PGF_06120144 | Ribosome association toxin RatA |
| 460 | PGF_10470343 | Ferric uptake regulation protein FUR |
| 461 | PGF_00016546 | Lactoylglutathione lyase (EC 4.4.1.5) |
| 462 | PGF_10450086 | Transcription termination protein NusB |
| 463 | PGF_03518570 | Nucleoside diphosphate kinase (EC 2.7.4.6) |
| 464 | PGF_02139563 | 6-carboxy-5,6,7,8-tetrahydropterin synthase (EC 4.1.2.50) |
| 465 | PGF_00005948 | Flavoprotein MioC |
| 466 | PGF_00049837 | SSU ribosomal protein S11p (S14e) |
| 467 | PGF_09669766 | Protein translocase subunit SecE |
| 468 | PGF_10123167 | 2-amino-4-hydroxy-6-hydroxymethyldihydropteridine pyrophosphokinase (EC 2.7.6.3) |
| 469 | PGF_00049909 | SSU ribosomal protein S9p (S16e) |
| 470 | PGF_00048933 | Ribosome-associated heat shock protein implicated in the recycling of the 50S subunit (S4 paralog) |
| 471 | PGF_05541499 | Biopolymer transport protein ExbD/TolR |
| 472 | PGF_00049906 | SSU ribosomal protein S8p (S15Ae) |
| 473 | PGF_00068794 | Autonomous glycyl radical cofactor |
| 474 | PGF_00023476 | Mutator MutT protein (7,8-dihydro-8-oxoguanine-triphosphatase) (EC 3.6.1.-) |
| 475 | PGF_00057032 | Tol-Pal system-associated acyl-CoA thioesterase |
| 476 | PGF_00006500 | Fumarate reductase subunit C |
| 477 | PGF_06941403 | SSU ribosomal protein S12p (S23e) |
| 478 | PGF_00049901 | SSU ribosomal protein S6p |
| 479 | PGF_09452281 | Ribosome-binding factor A |
| 480 | PGF_00060478 | Translation initiation factor 3 |
| 481 | PGF_00048539 | Ribonuclease E inhibitor RraB |
| 482 | PGF_01748119 | Sulfur acceptor protein => sulfur transfer pathway protein CsdE |
| 483 | PGF_00049840 | SSU ribosomal protein S13p (S18e) |
| 484 | PGF_00016358 | LSU ribosomal protein L20p |
| 485 | PGF_02961167 | 2-iminobutanoate/2-iminopropanoate deaminase RidA/TdcF (EC 3.5.99.10) |
| 486 | PGF_02294260 | Sigma factor RpoE regulatory protein RseC |
| 487 | PGF_01232993 | Tautomerase |
| 488 | PGF_03225027 | UPF0125 protein RatB |
| 489 | PGF_00016353 | LSU ribosomal protein L18p (L5e) |
| 490 | PGF_04845029 | LSU ribosomal protein L19p |
| 491 | PGF_01979294 | Dihydroneopterin aldolase (EC 4.1.2.25) |
| 492 | PGF_06210305 | hypothetical protein |
| 493 | PGF_09375481 | Uncharacterized protein YfgD, not an arsenate reductase |
| 494 | PGF_09545833 | Iron-sulfur cluster insertion protein ErpA |
| 495 | PGF_00567182 | YcfF/hinT protein: a purine nucleoside phosphoramidase |
| 496 | PGF_06545170 | Fluoride ion transporter CrcB |
| 497 | PGF_00006501 | Fumarate reductase subunit D |
| 498 | PGF_00016368 | LSU ribosomal protein L22p (L17e) |
| 499 | PGF_08057496 | Monothiol glutaredoxin GrxD |
| 500 | PGF_02141223 | Nucleoid-associated protein YaaK |
| 501 | PGF_00416617 | Carboxymuconolactone decarboxylase family protein |
| 502 | PGF_02501663 | UPF0102 protein YraN |
| 503 | PGF_00048898 | Ribosome-associated inhibitor A |
| 504 | PGF_00363133 | hypothetical protein |
| 505 | PGF_10456344 | Protein translocase membrane subunit SecG |
| 506 | PGF_00016377 | LSU ribosomal protein L24p (L26e) |
| 507 | PGF_03990071 | LSU ribosomal protein L23p (L23Ae) |
| 508 | PGF_00049844 | SSU ribosomal protein S14p (S29e) @ SSU ribosomal protein S14p (S29e), zinc-independent |
| 509 | PGF_03767152 | Proposed lipoate regulatory protein YbeD |
| 510 | PGF_00420145 | Cytochrome C553 (soluble cytochrome f) |
| 511 | PGF_00685189 | Protein translocase subunit YajC |
| 512 | PGF_01170625 | Cell division protein FtsL |
| 513 | PGF_00421913 | DNA uptake protein and related DNA-binding proteins |
| 514 | PGF_00013290 | Uncharacterized protein YqcC |
| 515 | PGF_09581668 | Heat shock protein 10 kDa family chaperone GroES |
| 516 | PGF_03793164 | Integration host factor alpha subunit |
| 517 | PGF_00425811 | dsDNA mimic protein, highly acidic |
| 518 | PGF_04287303 | RNA-binding protein YhbY |
| 519 | PGF_00060287 | Transcriptional repressor protein TrpR |
| 520 | PGF_00067423 | Z-ring-associated protein ZapA |
| 521 | PGF_00649271 | Cell division protein BolA |
| 522 | PGF_00422053 | DNA-binding protein Fis |
| 523 | PGF_05693577 | Integration host factor beta subunit |
| 524 | PGF_03048693 | Periplasmic nitrate reductase component NapD |
| 525 | PGF_04902248 | DNA-binding protein HU-alpha |
| 526 | PGF_02454577 | SSU ribosomal protein S20p |
| 527 | PGF_00038759 | Protein YihD |
| 528 | PGF_05906360 | DNA-directed RNA polymerase omega subunit (EC 2.7.7.6) |
| 529 | PGF_00435430 | Ribosomal silencing factor RsfA |
| 530 | PGF_00416927 | Cell division protein DivIC (FtsB), stabilizes FtsL against RasP cleavage |
| 531 | PGF_05076104 | Exodeoxyribonuclease VII small subunit (EC 3.1.11.6) |
| 532 | PGF_00008349 | Glutaredoxin 1 |
| 533 | PGF_08051111 | Phosphotransferase system, phosphocarrier protein HPr |
| 534 | PGF_00049854 | SSU ribosomal protein S17p (S11e) |
| 535 | PGF_10444145 | ATP synthase F0 sector subunit c (EC 3.6.3.14) |
| 536 | PGF_00065765 | Uncharacterized protein YbfE |
| 537 | PGF_06087691 | hypothetical protein |
| 538 | PGF_00064685 | Uncharacterized ferredoxin-like protein YfhL |
| 539 | PGF_00022566 | Molybdopterin synthase sulfur carrier subunit |
| 540 | PGF_02069837 | Succinate dehydrogenase flavin-adding protein, antitoxin of CptAB toxin-antitoxin |
| 541 | PGF_08212976 | Oxaloacetate decarboxylase Na(+) pump, gamma chain (EC 4.1.1.3) |
| 542 | PGF_00037063 | Probable exported or periplasmic protein in ApbE locus |
| 543 | PGF_00413253 | tRNA 5-methylaminomethyl-2-thiouridine synthesis sulfur carrier protein TusA |
| 544 | PGF_07822705 | Twin-arginine translocation protein TatA |
| 545 | PGF_00060431 | Translation initiation factor 1 |
| 546 | PGF_04034430 | SSU ribosomal protein S21p |
| 547 | PGF_00002132 | UPF0270 protein YheU |
| 548 | PGF_00038637 | Protein SlyX |
| 549 | PGF_00299130 | Cell division protein ZapB |
| 550 | PGF_03818138 | LSU ribosomal protein L35p |
| 551 | PGF_02899131 | LSU ribosomal protein L29p (L35e) |
| 552 | PGF_02189744 | Cytochrome c-type biogenesis protein CcmD, interacts with CcmCE |
| 553 | PGF_00016416 | LSU ribosomal protein L33p @ LSU ribosomal protein L33p, zinc-independent |
| 554 | PGF_04792416 | LSU ribosomal protein L32p @ LSU ribosomal protein L32p, zinc-independent |
| 555 | PGF_02174988 | Carbon storage regulator |
| 556 | PGF_00016418 | LSU ribosomal protein L34p |
| 557 | PGF_00016424 | LSU ribosomal protein L36p @ LSU ribosomal protein L36p, zinc-dependent |
