# Supplementary material for: Comparative genomics and in silico virulence assessment of nonpathogenic Avibacterium paragallinarum isolates from healthy layer chickens
Source: Front Microbiol. 2026 Sep 3;17:1919963. doi: 10.3389/fmicb.2026.1919963 (PMC13582619; doi:10.3389/fmicb.2026.1919963)
Supplement: Supplementary file 4 [file Table_3.docx]

***hmp* and *nsrR* in-house real-time PCR assays**

**Primers and probes:**

| ***hmp* Forward Primer** | ATT ATG CCA TTG TGG GCA AC |
| --- | --- |
| ***hmp* Reverse Primer** | CTG TGC TAA TCA AAA TAT CCG CTA |
| ***hmp* Probe** | CGG CGG TCC AGG CTT CAA TTA AT |
| ***nsrR* Forward Primer** | CAA TGA GCC GTT GGT AAA CT |
| ***nsrR* Reverse Primer** | TTA AGA TTT TGG TAA AAC TGC TCG |
| ***nsrR* Probe** | TCC TAA ATG TGG ATT ACA ACC ACA ATG CC |

**Recipe and Thermal profile:**

| Master Mix | Concentration per reaction | 1 reaction (µl) |
| --- | --- | --- |
| Nuclease free water | - | 8.145 |
| Forward primer (10 µM) | 400 nM | 0.800 |
| Reverse primer (10 µM) | 400 nM | 0.800 |
| Probe (25 µM) (FAM) | 200 nM | 0.160 |
| XIPC forward primer(100 µM) | 200 nM | 0.040 |
| XIPC reverse primer(100 µM) | 200 nM | 0.040 |
| XIPC probe (100 µM) (Cy5) | 75 nM | 0.015 |
| TaqMan Fast Virus 1-step Master Mix (5 µL) (Applied Biosystems, Carlsbad, CA, USA) | - | 5.000 |
| Total |  | 15.000 |
| Template |  | 5 |
| Total final volume |  | 20 |

**Results:**

All nonpathogenic Avibacterium paragallinarum isolates tested negative by both assays, whereas the positive control (a pathogenic A. paragallinarum field isolate) produced positive signals. Notably, both assays also generated positive signals with Avibacterium volantium isolates, due to the presence of a highly similar operon in this species.
