# Supplementary material for: Comparative genomics and in silico virulence assessment of nonpathogenic Avibacterium paragallinarum isolates from healthy layer chickens
Source: Front Microbiol. 2026 Sep 3;17:1919963. doi: 10.3389/fmicb.2026.1919963 (PMC13582619; doi:10.3389/fmicb.2026.1919963)
Supplement: Supplementary file 5 [file Table_5.docx]

**Sequence variation within translated ORFs across the whole genome**

**Summary of partially consistent divergent genes**

**Supplementary Table**

| **BV-BRC ID** | **Product** | **Query strain** | **Amino acids subject identity%/coverage% in npAP** | **Amino acids subject identity%/coverage% in pAP** |
| --- | --- | --- | --- | --- |
| **fig\|728.171.peg.2377** | Na (+) - translocating NADH-quinone reductase subunit C (EC 1.6.5.8) | **ESV-135** | 93-95% identity and 99% coverage in all npAP except for npAP/OH-USA/20230315/S2-1, which has 98% identity. | 99-100% identity and 99% coverage. |
| **fig\|728.713.peg.335** | Acetolactate synthase small subunit (EC 2.2.1.6) | **AG21-0333** | 100% identity, and 98.6% coverage | 71.8 – 97.2% identity, and 97.2-98.6% coverage |
