# Supplementary material for: Comparative genomics and in silico virulence assessment of nonpathogenic Avibacterium paragallinarum isolates from healthy layer chickens
Source: Front Microbiol. 2026 Sep 3;17:1919963. doi: 10.3389/fmicb.2026.1919963 (PMC13582619; doi:10.3389/fmicb.2026.1919963)
Supplement: Supplementary file 6 [file Table_6.docx]

**The entire amino acid sequence of the whole HMTp210 of npAP/IA-USA/20210120/S1-1 (AP-2)**

**The four main insertions are highlighted in yellow**

MNKVFKIKYSVVKQEMIVVSELANNKDKTATVKSSITPVIKPAGLFSRLSKLAISVALAL

GITLFSSSIAQAEEIWVSGGKIGRKNSITGQPSSSIALNPVSGREGYDTISTNSVAVGTG

AKAAADSTVSIGQGAVNESSAANGIAIGKQAKNVMEKGIVIGYNAYTKSSTEEAREGSVV

IGENAKSVGNQSVSLGQNAWSKSNSISIGSGTFAEGKSTIAMGTDKISGNNYNDQLPDTA

WNGTGNAPANSIWDIFSDLYMGKQANGNDYDAKTPDPNKKEAFYTFSDFKRRYVDNPSAS

PTYAGKLGAIALGSRTIAAGEMSTAVGSLAFALADKSTAMGLRSFVARGAEGGTAIGEES

RTFAENSVAIGNKTEASNAGSMAYGYQAKAVGEGAIAIGAKVAAGAQFDSRTTGNLLLNN

GAYAALRNADKSDDAKAENAINAFTQSFDTVLTNGLPLASENDTYLTTSAGAIKKTATTN

SNAGGGKNAIAIGNKTFASKTNSVALGSYALADAKNAFALGSYSFVEPSATNTIAIGVGS

YARGASSFLGGSWTSTLSDRTVVLGHAASISSGSRDALAMGVNAFIGSDSASSLALGMGS

TIAKNAKSPDSLAIGKDTRIDAKDTDNGTLYTPQVYNENTQTFRRFNADSDYMRQAMALG

FNAKVSRGAGKMETGINAMAIGAFAQATLQNSTALGVNSKTDYTWEQLEAEPWVSKGATS

IPTSGKIGVISVGSKGSERRIVNVASGSFDTDAVNVAQLKTIEERFQSEIDLLQNGGGVQ

YLSVEKTNVNGEAGRVASQIRKGESYERYVKLKTQLLYLDAREKLNGEKFNQTSLNKIRT

AVQELEAEYGGELKTTASELNKVATQLNGETTANNFEKFNRYKTQIENATNADSARNVGG

LTPEAIARLKANNNYLNDGAKGQDSIAFGWQAKTSMTGAVAIGKGATVTAGGKPSIALGQ

DSTVANSASRTSSATINGLTFNNFAGSPETLGVLSIGTAGKERKIVNVAAGDISQASTEA

INGSQLYATNFMLSKVAQSVKSNFGGDANLATDGTITFTDIGGTRQNTIHDAINNVRTNA

ANKDLSNLSDNGKNAITGLVDVVKKADSPITVDPSTDNNKKKTFTVGVDFTDTITEGDTT

DDKKLTTAKSVENYVTNKLTNFSTDILLSDGRGGNETTQNDGTGKRSLSDGFTIKADNFN

LDNVNYRGDKNIKVLYDDQNGVFKIGLAPLLLLNQGIQIDRKNVANIFGGDYGYTTSLTD

QGLTFQYDLTVNGNSDSTDLPKANYGATGLSIDYSRNPNGTKFKASMDESGVHFEKGAGT

QKTIADLTGEALTLASNAQKMKVDNTDGSVKLLSSADAQQAAIQLKSDKILLQGANATTP

ITLDGVKAATISATSKEAVNGSQLNSLLTTVLGAGADLNAVGFTGINNASPMTYKAAIEA

LAGSTIALSGNTGNTTTKKITDNPTFTIKGDGTDITSNAATDAVTFTLNKATTVAQNDNK

AVTSSAVYSAIESAKPTVSAKQGESILKVEGTKTGGITGDSFKLSLDQTALKKALETDFA

KVDASNVAGNAEKWRQAIDVYSKSETNDAIKSSKVTLTPQNGLISATKTTTGTGNQGDTW

TLAVNSTDSIDKNNATGGKLVTDKAVKDYVAQSSILRTKGNNATAGGPATGQVDLSANQI

LTVEGVNNGADISTNATGNNISLTLNKATSITNSDSKAATSQAVYDAIKTAKASITAATG

GIITVSGTKGDNINSDNWTIGVNYTSDNQIGTNPTGNKLATDLAVKNAVDGAVDKVKILR

IAGDASSSGQVDLSQNQILGITGTENQIETTARGNNITLKLSNTLMSKIDGKVDNTDYQN

ATNITASKWQEALSIAYKANSDAQKTVSLAKGLAFTNGTNTTAEVGDEGVVKFNLNKDLT

GISSIEGESGKAKLTFGDALTLNGKKLTGLADGTISTGSKDAITGGQLRALLGADPSSSN

TFTNFGGVTGATTVQAAIQTLNTNLSKGYKVKADKDVNSPPVAGTEHKLDLGTTLNVLTD

ATPWGETNGAMYSGENLQTSFNGTNNLYIGLKEKPTFKGLTISNGTNAGSPQSIVFDTLG

VKGTFGGNKTTGFEIGDGKILFKQAGGSTENVQLKNVADGVDDNDAVNFKQHKDLASLSN

TGKKVIQDQVNVQKSDSQNFITVTKEAGTDDGSTPKTYKVGLNTASKDDLDKNNGLSDKI

ATADATKNYVDEKVNGLSQNLSLTDGTANESLNLKAEKLAVVGTANQTTVALTKDDTQKT

KTLTVGLAEDIQGVNSITFKESGSAPNNITGKMSSEGLTFKKGTDTDGSTTTFAEDGLTI

DSTTNSAQTNLVKVSREGFSVKDNTGESKLAPTKLSIGAENAEHVEVTKSGIALKENSTT

GKSSITLAQDAITLAGNATGTAIKLTGVADGAITTASKDAINGGQLRDILGFDPTSKQNT

AGQGIGGTGSKTIEGAVKEIKDSVTGAKLKYTANSGTQKQEVLLSDGLDFKNGDNTTATV

AASGEVKFSVNKTLEGMNTVEFDQQNGVGDKAQLNASGLVFAADPTNTTAGNSTKFASDG

VTLVKNGTTVKLANDGFGIQNGTSQSKLGADKLTIGNPAAQHTVLENGTFARYNGQTKGT

NIALSDDAITVSNGTGNIRVTGVANATGDNDAVNFGQYKDLQNVTDKAKKLIQDQIVVTN

KDNFVSVSAENDQNGTAPKTYKVGVNTVTDLTNGGTLTDKIATADATKNYVDQKVKGLSQ

TLDLSDGTTDKSLDLKSEKLKVVGTEKQTSVVLTDNDAASPKEKILTVGLVKDIEGVNSI

TFDKSGQDPNQVTGRMSSAGLTFKKGTDTDGSTTTFAEDGLTIDSTTNSAQTNLVKVSRD

GFSVKNGSDESKLAPTKLSIGAENAEHVEVTKTGIALKADDTIGKSSITLSDSAITLAAA

TAGNAIKLTGVADGNITTDSKDAVNGGQLHDVLGFDPKDKQNTAGQGIGGTGSKTIDGAI

SEVKESLTNATLAYKADNKNGKTVKLTDGLNFTSTTNIDASVEESGVVKFTLKDQLTGLK

TIATESLNASQNIIAGGTVTVGGETEGIVLTKSGSGNDRTLSLSGAGNAATDGIKVSGVK

AGTAETDAVNKGQLDKLSKAINDALGTTDLAVTKDPNKTSIFDPINGTAPTTFKDAVDKL

TTAVNTGWGLNVGSTNKNIGAGKTVTVAGSNGITANLSNDDSPTLTIGLETTTFNTTNPA

NISAADTGDKFATAANVASAINAATAGLSQTLKFKANGDNTAQSLNLKTQTLEFNKKDEN

LTVSSTGTSITYGLSNNITLGDSTNAGQITIKRGAGSNPDLVIGKDGKITGLADGSIATN

STDAVNGGQIRSILGVDPNSKIGGTEKTNISEAIKEVNDKADKLAATNLQYKATNSQGAT

KQNSQTPAQEQKGEVPLTTGLDFRDGKNTTSEVSDNGVVKYHLNETLDDMDTINFKAQPN

QTNKAKLNADGLVIADDPNNISTGNYTALGKDGVTLVKGDSTVKLADEGFSVKNATSESK

LGADKLTLGKTGENHLEITQDTISAKNGSNGKSGINFTENTVTLVGKDGTNSTAKLTGLS

DGAIGADSKDAINGKQIYDVLGFDPATKKNEAGLGIGGTSSKTVDGAIKEVKDSVTNATL

KYTANSGTTKKEVKLSEGLDFVNGDNTTASVEDNGKVKVSVNTTLKDMDTINFKAKTTDK

AALSSDGLVFDDSTTAAQTDRTTFAKDGVTLEKDGSTVKVAKDGFAVKDTTSQSKVESNK

LSVGDVAGNAFNDHLEVSKTGISRLGGTEENLVSLTLGNKAVTLAGKDSAAVALKGVSSA

LGLTAGATAVEKATAQSKMTDLLAKTNGDLNTAVNLADLQAVAQAGLDFAGNSGELHRTL

GSKLNVKGEGNVTGTTAADNITVEANNDTLTIKLSNNITNISSIGGGKKTNPNTEEKAKI

TFEDDSLKVNDKTLTGVKSALDGVQVMDGNNTPKQNPTILEKLDTAAKDTQGGSSSVVNV

KDLSDVATALTDKGLSFVGDDSQKVDRKLGETLKLIGGANKDQLSDGNNIGVTKDTTDGT

LKLRLAKDLSQIASIAFDTAGGVTGKISSEGTVFTKGDKNNGGETKTTFGVDGVTIDNTK

TAADGTDAKTTLVKVATDGFSVTDGNSESKLGADKLTIGKSGEDHLEISKDTITAKNGAD

GKSSIKLAENTVTLVGKDGNNATAKLTGVADGSMAAGSNDAVNGGQINKILGFDPAELEK

PNGSKTGIGGTTSKTIDGAIEEVKNSVVNAELTYRANDETDDQAKKVKLSKGLNFKNGTN

TTATVDNDGVVTYSLNATLNEMDTINFATKNNNNATLNSDGLVFNSDPKDGNHTTFAKDG

VTLTKAATDGTVSTIKVAEDGFSVKAVDNTGAVQNESKLGADKLTIGTPTADHFEVSKTG

ISRLGGEDKNTVSLTLGENAVTLAGKDSAAVALKGVSSALGLSATTVSKDDAKTKVEELL

KKNNTELNTAVNLADLQAVAQSGFDFAGNSGNEIHRTLGTKLTIKGEGTLNGDTAADNLT

VVSDGTDTLTVKLSKNLTNINSIGGGNTAAGAEKAKITFEDDKLKVNDKTLTGVKSALDS

VQVMDGNTPKQNPTILEKLDTAAKDTQGGSSSVVNVKDLSDVATALTDKGLSFVGDDSQK

VDRKLGETLKLIGGANKDQLSDGNNIGVTKDTTDGTLKLRLAKELSQIASIAFDTVGGVT

GKISSEGTVFTKGEKNNGGETKTTFGVDGVTIDNTKTAADGTDAKTTLVKVATDGFSVTD

GNSESKLGADKLTIGTPTADHLEVSKTGISRLGGADKDIVSMTLGENAVTLAGKDSAAVA

LKGVSSALGLSATTVSKGDAKTKVEELLKKNNTELNTAVNLADLQAVAQSGFDFAGNSGG

DIHRTLGTKLTIKGEGTLTGDTAADNLTVVSDGTDTLTVKLSKNLTNINSIGGGNTAAGA

EKAKITFEDDKLKVNDKTLTGVKSALDSVQVMDSNNTPKQNPTILEKLDAAAKDTQGGSS

SVVNVKDLSDVATALTDKGLSFVGDDSQKVDRKLGETLKLIGGASTNLSDGANIGVTKDT

ADGTLKLRLAKDLSQIASVAFDPTASGVIGRISSEGTVFTKGDKANGGETTTTFGIDGVT

IDNTKTVDGADVKTNLVKVATDGFSVKQGENESKLSPTKLSIGKETGSHIEVTNEGIALK

ENTTDKSSIKLGTDAITLAGNAANQPIKLTGVANGSMAANSNDAVNGGQINKILGFDPAD

FDKQTDPKTGIGGTNAKTIEGAISALNNKKLKYIASGANGTQRGNDQEVDITNGLNFKDG

KNTTASVDANGVVQYALNDTLEEMDTINFAQQGGTGDKAKLNANGLVFAADPNNIQTGDA

TVFAKDGVTLTKAATDGTVSTIKVAEDGFSVKKVDGQGSVQNESKLGADKLTIGTPTADH

FEATNTGIARKNGTANISSIELGTDAITLAGANATDGITLKGVKGGEISSTSKEAINGTQ

LNDLGATVLGATVKDDKTGFVAPDFAAINGKKDKPTTFKAAIDQLIEGMNSGINLKGSNS

TDAHKALLGSTITIKQGDYTDNGTQYKGENLSVTLDNASQTFTVAMKKDPRFDTVKVGTD

GENQLVINGSEISSKVKGADGTVSDGAKVTLGKDEITLSKGTGSDPVELKGVASAIDKVT

KVTKDDGTENSNPTVLEKLEQAAKDGASSVVNVKDLNNVAKAVQDSLTEKGLTFADDNGT

EVKRKLGETLTVKGGVQETQDPNAEKKLTDNNIGVVSDTATNALVVKLAKEISDITSIAT

NTAGSSKSVFDTEGVTVTGLNQDNSNGQSQPTETGAKAIYGLNGVSVKDTEGNTSTTNAT

TTQFVDKEGKTSMQTASGVVTVDGIHTSVLKADSMTMTDQNGHSQLTANELSLIDNDGNN

NIQTAAGTRLTSKDGDITAVTGKGIMSISPDRTKTVSLSSEGISLNSPNGGVNVTSEGFG

FTGANNQFRTDAPRILATGIDAGNKKISNVADGDISPTSGDVVTGRQLYALMQKGIRVYG

DEVSPTKTQTTAPTNANPTATTAPTNANPTATTAPTASSTQGGATTANTAGGVAPAGNVA

TGDIAPTQPTLPEMNTALVDDHLAVPLGGSLKIHGDHNVKTTISADNQVGISLQPNISIE

NNLVIGSNDPEKAKLAAQEGNALVITNKDDGNAAMVFNNEKNMLVLSDKEAKPRAVLDGQ

NGALTLVGNDDSQVTLSSKKGKDIDGNDLSRLSVTTERTNANGQLEKVETSFATMDDGLK

FKADGDKVINKKLNETVEIVGDENVTTSITDNKEVKVSLNKKIAIDEVKIPNTDPDAQEG

DSIVINNGGIHAGNKVITGVKASDDPTSAVNRGQLNTVIDNVQNNFNQVNQRIGDLTRES

RAGIAGAMATASLQNVVLPGKTTISVGTATFKGENAVAIGMSRLSDNGKVGIRLSGMSTS

NGDKGAAMSVGFTF

**BlastP results of the translated npAP HMTp210 four main insertions**

**>Insertion 1 starts at amino acid position 1294 in npAP/IA-USA/20210120/S1-1 (AP-2)**

YSRNPNGTKFKASMDESGVHFEKGAGTQKTIADLTGEALTLASNAQKMKVDNTDGSVKLLSSADAQQAAIQLKSDKILLQGANATTPITLDGVKAATISATSKEAVNGSQLNSLLTTVLGAGADLNAVGFTGINNASPMTYKAAIEALAGSTIALSGNTGNTTTKKITDNPTFTIKGDGTDITSNAATDAVTFTLNKATTVAQNDNKAVTSSAVYSAIESAKPTVSAKQGESILKVEGTKTGGITGDSFKLSLDQTALKKALETDFAKVDASNVA

**>Insertion 2 starts at amino acid position 1609 in npAP/IA-USA/20210120/S1-1(AP-2)**

TTTGTGNQGDTWTLAVNSTDSIDKNNATGGKLVTDKAVKDYVAQSSILRTKGNNATAGGPATGQVDLSANQILTVEGVNNGADISTNATGNNISLTLNKATSITNSDSKAATSQAVYDAIKTAKASITAATGGIITVSGTKGDNINSDNWTIGVNYTSDNQIGTNPTGNKLATDLAVKNAVDGAVDKVKILRIAGDASSSGQVDLSQNQILGITGTENQIETTARGNNITLKLSNTLMSKIDGKVDNTDYQNATNITASKWQEALSIAYKANSDAQKTVSLAKGLAFTNGTNTTAEVGDEGVVKFNLNKDLTGISSIEGESGKAKLTFGDALTLNGKKLTGLADGTISTGSKDAITGGQLRALLGADPSSSNTFTNFGGVTGATTVQAAIQTLNTNLSKGYKVKADKDVNSPPVAGTEHKLDLGTTLNVLTDATPWGETNGAMYSGENLQTSFNGTNNLYIGLKEKPTFKGLTISNGTNAGSPQSIVFDTLGVKGTFGGNKTTGFEIGDGKILFKQAGGSTENVQLKNVADGVDDNDAVNFKQHKDLASLSNTGKKVIQDQVNVQKSDSQNFITVTKEAGTDDGSTPKTYKVGLNTASKDDLDKNNGLSDKIATADATKNYVDEKVNGLSQNLSLTDGTANESLNLKAEKLAVVGTANQTTVALTKDDTQKTKTLTVG

**>Insertion 3 starts at amino acid position 2383 in npAP/IA-USA/20210120/S1-1(AP-2) (This insertion is absent in npAP/OH-USA/20210309/S1-1 (AG21-0333) and npAP/OH-USA/20230213/S1-2)**

EHVEVTKSGIALKENSTTGKSSITLAQDAITLAGNATGTAIKLTGVADGAITTASKDAINGGQLRDILGFDPTSKQNTAGQGIGGTGSKTIEGAVKEIKDSVTGAKLKYTANSGTQKQEVLLSDGLDFKNGDNTTATVAASGEVKFSVNKTLEGMNTVEFDQQNGVGDKAQLNASGLVFAADPTNTTAGNSTKFASDGVTLVKNGTTVKLANDGFGIQNGTSQSKLGADKLTIGNPAAQHTVLENGTFARYNGQTKGTNIALSDDAITVSNGTGNIRVTGVANATGDNDAVNFGQYKDLQNVTDKAKKLIQDQIVVTNKDNFVSVSAENDQNGTAPKTYKVGVNTVTDLTNGGTLTDKIATADATKNYVDQKVKGLSQTLDLSDGTTDKSLDLKSEKLKVVGTEKQTSVVLTDNDAASPKEKILTVGLVKDIEGVNSITFDKSGQDPNQVTGRMSSAGLTFKKGTDTDGSTTTFAEDGLTIDSTTNSAQTNLVKVSRDGFSVKNGSDESKLAPTKLSIGAENA

**>Insertion 4 starts at amino acid position 3194 in npAP/IA-USA/20210120/S1-1(AP-2) (This Insertion is very short in npAP/GA-USA/20231216/S1-1)**

STNKNIGAGKTVTVAGSNGITANLSNDDSPTLTIGLETTTFNTTNPANISAADTGDKFATAANVASAINAATAGLSQTLKFKANGDNTAQSLNLKTQTLEFNKKDENLTVSSTGTSITYGLSNNITLGDSTNAGQITIKRGAGSNPDLVIGKDGKITGLADGSIATNSTDAVNGGQIRSILGVDPNSKIGGTEKTNISEAIKEVNDKADKLAATNLQYKATNSQGATKQNSQTPAQEQKGEVPLTTGLDFRDGKNTTSEVSDNGVVKYHLNETLDDMDTINFKAQPNQTNKAKLNADGLVIADDPNNISTGNYTALGKDGVTLVKGDSTVKLADEGFSVKNATSESKLGADKLTLGKTGENHLEITQDTISAKNGSNGKSGINFTENTVTLVGKDGTNSTAKLTGLSDGAIGADSKDAINGKQIYDVLGFDPATKKNEAGLGIGGTSSKTVDGAIKEVKDSVTNATLKYTANSGTTKKEVKLSEGLDFVNGDNTTASVEDNGKVKVSVNTTLKDMDTINFKAKTTDKAALSSDGLVFDDSTTAAQTDRTTFAKDGVTLEKDGSTVKVAKDGFAVKDTTSQSKVESNKLSVGDVAGNAFNDHLEVSKTGISRLGGTEENLVSLTLGNKAVTLAGKDSAAVALKGVSSALGLTAGATAVEKATAQSKMTDLLAKTNGDLNTAVNLADLQAVAQAGLDFAGNSGELHRTLGSKLNVKGEGNVTGTTAADNITVEANNDTLTIKLSNNITNISSIGGGKKTNPNTEEKAKITFEDDSLKVNDKTLTGVKSALDGVQVMDGNNTPKQNPTILEKLDTAAKDTQGGSSSVVNVKDLSDVATALTDKGLSFVGDDSQKVDRKLGETLKLIGGANKDQLSDGNNIGVTKDTTDGTLKLRLAKDLSQIASIAFDTAGGVTGKISSEGTVFTKGDKNNGGETKTTFGVDGVTIDNTKTAADGTDAKTTLVKVATDGFSVTDGNSESKLGADKLTIGKSGEDHLEISKDTITAKNGADGKSSIKLAENTVTLVGKDGNNATAKLTGVADGSMAAGSNDAVNGGQINKILGFDPAELEKPNGSKTGIGGTTSKTIDGAIEEVKNSVVNAELTYRANDETDDQAKKVKLSKGLNFKNGTNTTATVDNDGVVTYSLNATLNEMDTINFATKNNNNATLNSDGLVFNSDPKDGNHTTFAKDGVTLTKAATDGTVSTIKVAEDGFSVKAVDNTGAVQNESKLGADKLTIGTPTADHFEVSKTGISRLGGEDKNTVSLTLGENAVTLAGKDSAAVALKGVSSALGLSATTVSKDDAKTKVEELLKKNNTELNTAVNLADLQAVAQSGFDFAGNSGNEIHRTLGTKLTIKGEGTLNGDTAADNLTVVSDGTDTLTVKLSKNLTNINSIGGGNTAAGAEKAKITFEDDKLKVNDKTLTGVKSALDSVQVMDGNTPKQNPTILEKLDTAAKDTQGGSSSVVNVKDLSDVATALTDKGLSFVGDDSQKVDRKLGETLKLIGGANKDQLSDGNNIGVTKDTTDGTLKLRLAKELSQIASIAFDTVGGVTGKISSEGTVFTKGEKNNGGETKTTFGVDGVTIDNTKTAADGTDAKTTLVKVATDGFSVTDGNSESKLGADKLTIGTPTADHLEVSKTGISRLGGADKDIVSMTLGENAVTLAGKDSAAVALKGVSSALGLSATTVSKGDAKTKVEELLKKNNTELNTAVNLADLQAVAQSGFDFAGNSGGDIHRTLGTKLTIKGEGTLTGDTAADNLTVVSDGTDTLTVKLSKNLTNINSIGGGNTAAGAEKAKITFEDDKLKVNDKTLTGVKSALDSVQVMDSNNTPKQNPTILEKLDAAAKDTQGGSSSVVNVKDLSDVATALTDKGLSFVGDDSQKVDRKLGETLKLIGGASTNLSDGANIGVTKDTADGTLKLRLAKDLSQIASVAFDPTASGVIGRISSEGTVFTKGDKANGGETTTTFGIDGVTIDNTKTVDGADVKTNLVKVATDGFSVKQGENESKLSPTKLSIGKETGSHIEVTNEGIALKENTTDKSSIKLGTDAITLAGNAANQPIKLTGVANGSMAANSNDAVNGGQINKILGFDPADFDKQTDPKTGIGGTNAKTIEGAISALNNKKLKYIASGANGTQRGNDQEVDITNGLNFKDGKNTTASVDANGVVQYALNDTLEEMDTINFAQQGGTGDKAKLNANGLVFAADPNNIQTGDATVFAKDGVTLTKAATDGTVSTIKVAEDGFSVKKVDGQGSVQNESKLGADKLTIGTPTADHFEATNTGIARKNGTANISSIELGTDAITLAGANATDGITLKGVKGGEISSTSKEAINGTQLNDLGATVLGATVKDDKTGFVAPDFAAINGKKDKPTTFKAAIDQLIEGMNSGINLKGSNSTDAHKALLGSTITIKQGDYTDNGTQYKGENLSVTLDNASQTFTVAMKKDPRFDTVKVGTDGENQLVINGSEISSKVKGADGTVSDGAKVTLGKDEITLSKGTGSDPVELKGVASAIDKVTKVTKDDGTENSNPTVLEKLEQAAKDGASSVVNVKDLNNVAKAVQDSLTEKGLTFADDNGTEVKRKLGETLTVKGGVQETQDPNAEKKLTDNNIGVVSDTATNALVVKLAKEISDITSIATNTAGSSKSVFDTEGVTVTGLNQDNSNGQSQPTETGAKAIYGLNGVSVKDTEGNTSTTNATTTQFVDKEGKTSMQTASGVVTVDGIHTSVLKADSMTMTDQNGHSQLTANELSLIDNDGNNNIQTAAGTRLTSKDGDITAVTGKGIMSISPDRTKTVSLSSEGISLNSPNGGVNVTSEGFGFTGANNQFRTDAPR

**BlastP results: (C denotes coverage (%) and I denotes amino acid identity (%))**

**Multiple percentages per species are due to different strains**

**Insertion 1 showed hits with:**

- YadA-like family protein in *Fusobacterium* sp, **C** 65% and **I** 36.55%
- YadA-like family protein in *Gallibacterium anatis* **C** 47% and 67% with **I** 53% and 39.4%, respectively.
- YadA-like family protein of *Histophilus somni*, **C** 33% - 71% with **I** 34.7% - 48.4%.
- YadA-like family protein *Veillonella magna*, **C** 45% - 63% with **I** 36.4% - 41.7%.
- Adhesin 2 of npAP/IA-USA/20210120/S1-1 (AP-2) **C** 37%, and **I** 45%.

**Insertion 2 showed hits with:**

- YadA-like family protein, *Pasteurella sp. PK-2025*, **C** 55% and **I** 35%.
- YadA-like family protein, *Gallibacterium anatis*, **C** 45% and **I** 30.64%.
- Adhesin 2 of pAP strains AV26, AV25, and AV24, **C** 85% and **I** 40 %, in addition to hits with many other pAP adhesin 2 but with lower C and I.
- YadA-like family protein, *Gallibacterium trehalosifermentans*, **C** 49 % and **I** 47.13%.

**Insertion 3 showed hits with:**

- Hemagglutinin of *AP ENV-1065* **C** 100% , **I** 64.32%.

**Insertion 4 showed hits with:**

- ESPR-Type extended signal peptide-containing protein of *Veillonella parvula*, **C** 45%, and **I** 32.42%.
- YadA-like family protein, *Mergibacter septicus*, **C** 39% C and **I** 33.7%.
- YadA-like family protein, *Gallibacterium anatis* **C** 31% - 38% and **I** 35.71%-41.84%
- Adhesin 3 in many pAP and npAP with **C** around 35% and **I** around 38%
