# Supplementary material for: Comparative genomics and in silico virulence assessment of nonpathogenic Avibacterium paragallinarum isolates from healthy layer chickens
Source: Front Microbiol. 2026 Sep 3;17:1919963. doi: 10.3389/fmicb.2026.1919963 (PMC13582619; doi:10.3389/fmicb.2026.1919963)
Supplement: Supplementary file 7 [file Table_7.docx]

A. **Amino acid identity (%) of npAP capsule biosynthesis genes compared with the corresponding reference sequences of Genotype I (strain 221) and Genotype II (strain H18).**

| npAP genomes | Genotype **I**  Strain **221**  (GenBank FJ965541) | | | | | | Genotype **II**  Strain **H18**  (GenBank FJ965542) | | | | | |
| --- | --- | --- | --- | --- | --- | --- | --- | --- | --- | --- | --- | --- |
|  | AcbE* | AcbD | AcbG | AcbH | AcbC | AcbB | CcbE* | CcbF1 | CcbF2 | IS1016** | CcbC | CcbB |
| npAP/GA-USA/20231216/S1-1 | 57.9, 59.5 | No hits | No hits | No hits | 81.7 | 57 | 96, 93 | 99 | 98.7 | absent | 98.9 | 98 |
| npAP/GA-USA/20231216/S1-2 | 57.9, 59.5 | No hits | No hits | No hits | 81.7 | 57 | 96, 93 | 99 | 99 | absent | 98.9 | 98 |
| npAP/GA-USA/20231220/S2-1 | 57.9, 59.5 | No hits | No hits | No hits | 81.7 | 57 | 96, 93 | 99 | 99 | absent | 98.9 | 98 |
| npAP/GA-USA/20231220/S2-2 | 57.9, 59.5 | No hits | No hits | No hits | 81.7 | 57 | 96, 93 | 99 | 99 | absent | 98.9 | 98 |

*Two percentages are shown for AcbE and CcbE because their corresponding ORFs in npAP are split into two fragments due to a premature stop codon. Each percentage corresponds to one fragment. ** IS1016 is an insertion sequence present in the capsule biosynthesis locus of strain H18

B. **Amino acid substitutions in the npAP WaaQ protein compared to the corresponding protein in the type strain NCTC11296**

| npAP genomes | NCTC11296 WaaQ | | | | | | | | | | | | |
| --- | --- | --- | --- | --- | --- | --- | --- | --- | --- | --- | --- | --- | --- |
|  | A43 | K54 | I55 | S64 | A139 | Q162 | A179 | N181 | A201 | S209 | Q215 | N239 | P281 |
| npAP/IA-USA/20210120/S1-1 (AP-2) | - | E | V | N | - | R | E | D | - | A | - | - | - |
| npAP/OH-USA/20210309/S1-1 (AG21) | - | E | - | - | - | - | - | - | - | A | - | - | - |
| npAP/OH-USA/20230213/S1-2 | V | E | - | - | - | R | E | D | - | A | - | - | - |
| npAP/OH-USA/20230213/S1-3 | V | E | - | - | - | R | E | D | - | A | - | - | - |
| npAP/OH-USA/20230213/S1-4 | V | E | - | - | - | R | E | D | - | A | - | - | - |
| npAP/OH-USA/20230315/S2-1 | - | E | - | - | - | - | - | - | - | A | - | - | - |
| npAP/FL-USA/20231025/S1-1 | - | E | - | - | - | - | - | - | - | A | - | - | - |
| npAP/GA-USA/20231216/S1-1 | - | E | V | N | - | - | - | - | - | A | K | - | - |
| npAP/GA-USA/20231216/S1-2 | - | E | V | N | - | - | - | - | - | A | K | - | - |
| npAP/GA-USA/20231220/S2-1 | - | E | V | N | - | - | - | - | - | A | K | - | - |
| npAP/GA-USA/20231220/S2-2 | - | E | V | N | - | - | - | - | - | A | K | - | - |
| npAP/GA-USA/20231220/S2-3 | - | E | V | N | V | R | E | D | V | A | - | D | S |
| npAP/GA-USA/20231220/S2-4 | - | E | V | N | V | R | E | D | V | A | - | D | S |
| npAP/NC-USA/20240110/S1-1 | - | E | - | - | - | - | E | D | - | A | K | - | - |

(–) indicates no amino acid substitutions. Yellow-shaded positions denote substitutions observed exclusively in npAP, whereas the remaining substitutions were also reported in pAP.

C. The 16 glycine-rich repeats identified in the npAP AvxA (NCTC11296 numbering)

634GGAGNDIIQ

663GGRGNDVIY

682GGDGDDIIL

745GGAGNDMLS

763GGEGEDIVE

1508GGRGDDMLF

1535GGSGKDIFV

1665GGLGDDVLV

1690GGDGNDIVR

1699GGAGDDILY

1708GGNGLDSLG

1758GGSGNDKFI

1968GGLGDDVMI

1977GGKGDDIYL

2058GGNGSDFLM

2067GGVGNDTYI

D. Promoter region analysis of the F17-like fimbriae *flfADCG* operon in npAP. The number in parentheses beside each name indicates the number of C repeats (**bolded**). The start codon of *flfA* is highlighted in green.

npAP/OH-USA/20230213/S1-2 (12)

TTG**CCCCCCCCCCCC**GAAATTTTGTATACTCCGAGCGAATTTACTTAATCCTTAAGGAGTATTGAAAAATG

npAP/OH-USA/20230213/S1-3 (10)

TTG**CCCCCCCCCC**GAAATTTTGTATACTCCGAGCGAATTTACTTAATCCTTAAGGAGTATTGAAAAATG

npAP/OH-USA/20230213/S1-4 (11)

TTG**CCCCCCCCCCC**AAATTTTGTATACTCCGAGCGAATTTACTTAATCCTTAAGGAGTATTGAAAAATG

npAP/OH-USA/20230315/S2-1 (13)

TTG**CCCCCCCCCCCCC**GAAATTTTGTATACTCCAGGCGAATTTACTTAATCCTTAAGGAGTATTGAAAAAATG

npAP/GA-USA/20231216/S1-1 (13)

TTG**CCCCCCCCCCCCC**GAAATTTTGTATACTCCAGGCGAATTTACTTAATCCTTAAGGAGTATTGAAAAAATG

npAP/GA-USA/20231216/S1-2 (10)

TTG**CCCCCCCCCC**GAAATTTTGTATACTCCAGGCGAATTTACTTAATCCTTAAGGAGTATTGAAAAAATG

npAP/GA-USA/20231220/S2-1 (13)

TTG**CCCCCCCCCCCCC**GAAATTTTGTATACTCCAGGCGAATTTACTTAATCCTTAAGGAGTATTGAAAAAATG

npAP/GA-USA/20231220/S2-2 (13)

TTG**CCCCCCCCCCCCC**GAAATTTTGTATACTCCAGGCGAATTTACTTAATCCTTAAGGAGTATTGAAAAAATG

npAP/GA-USA/20231220/S2-3 (10)

TTG**CCCCCCCCCC**GAAATTTTGTATACTCCGAGCGAATTTACTTAATCCTTAAGGAGTATTGAAAAAATGAAAAAAATTG

npAP/GA-USA/20231220/S2-4 (10)

TTG**CCCCCCCCCC**GAAATTTTGTATACTCCGAGCGAATTTACTTAATCCTTAAGGAGTATTGAAAAAATGAAAAAAATTG

npAP/FL-USA/20231025/S1-1 **could not be determined because of incomplete contig**

E. **Amino acid substitutions in the npAP Glutathione reductase compared to the corresponding protein in the type strain NCTC11296**

| npAP genomes | NCTC11296 Glutathione reductase | | | | | | | | | | | | | | | |
| --- | --- | --- | --- | --- | --- | --- | --- | --- | --- | --- | --- | --- | --- | --- | --- | --- |
|  | S25 | A75 | V76 | D78 | S82 | K149 | A151 | V160 | A165 | A179 | A219 | D281 | V295 | P331 | A366 | V397 |
| npAP/IA-USA/20210120/S1-1 (AP-2) | G | - | - | - | - | - | - | - | - | - | - | - | - | S | - | A |
| npAP/OH-USA/20210309/S1-1 (AG21) | - | - | - | - | - | Q | - | - | - | - | - | - | M | - | - | - |
| npAP/OH-USA/20230213/S1-2 | - | E | Q | G | A | - | - | - | - | - | T | - | - | - | A | - |
| npAP/OH-USA/20230213/S1-3 | - | E | Q | G | A | - | - | - | - | - | T | - | - | - | A | - |
| npAP/OH-USA/20230213/S1-4 | - | E | Q | G | A | - | - | - | - | - | T | - | - | - | A | - |
| npAP/OH-USA/20230315/S2-1 | - | E | Q | G | A | - | T | M | - | - | - | N | - | - | A | - |
| npAP/FL-USA/20231025/S1-1 | - | - | - | - | - | - | - | - | - | - | - | N | - | - | - | - |
| npAP/GA-USA/20231216/S1-1 | - | E | Q | G | A | - | T | - | - | S | - | N | - | - | - | - |
| npAP/GA-USA/20231216/S1-2 | - | E | Q | G | A | - | T | - | - | S | - | N | - | - | - | - |
| npAP/GA-USA/20231220/S2-1 | - | E | Q | G | A | - | T | - | - | S | - | N | - | - | - | - |
| npAP/GA-USA/20231220/S2-2 | - | E | Q | G | A | - | T | - | - | S | - | N | - | - | - | - |
| npAP/GA-USA/20231220/S2-3 | - | E | Q | G | A | - | - | - | E | - | - | - | - | - | - | - |
| npAP/GA-USA/20231220/S2-4 | - | E | Q | G | A | - | - | - | E | - | - | - | - | - | - | - |
| npAP/NC-USA/20240110/S1-1 | - | E | Q | G | A | - | - | - | - | - | - | - | - | - | - | - |

(–) indicates no amino acid substitutions
