# Supplementary material for: Comparative genomics and in silico virulence assessment of nonpathogenic Avibacterium paragallinarum isolates from healthy layer chickens
Source: Front Microbiol. 2026 Sep 3;17:1919963. doi: 10.3389/fmicb.2026.1919963 (PMC13582619; doi:10.3389/fmicb.2026.1919963)
Supplement: Supplementary file 8 [file Table_8.docx]

***ccbE* amplification and Sanger sequencing**

**Primers:**

*ccbE* Forward: CATTCTAGCGTAGAGCAAGCA

*ccbE* Reverse: TGCAATTCCTTAGCAATCTTTTCT

**Recipe and Thermal profile:**

A total of 50 µL reaction mix using Superscript III One-Step RT-PCR System with Platinum Taq DNA Polymerase (ThermoFisher Scientific, USA), forward primer 2 µL (0.4 µmol final concentration), reverse primer 2 µL (0.4 µmol final concentration), and 14 µL of PCR grade water was applied.

The following thermal profile was adopted based on primers melting temperature: 95 ℃ for 20 s (1 cycle) followed by 40 cycles of 95 ℃ for 15 s, 55 ℃ for 10 s, and 68 ℃ for 1 min then final extension at 68 ℃ for 5 min (1 cycle).

Positive PCR products were purified using the ExoSAP [1] and submitted for Sanger sequencing (DNA facility – Iowa State University).

**Generated consensus:**

CAAGCATTATCTTTAGATGCTAACAAGATAGATAATAAACTACAGCAATGGCAACAATATAACCAAAAAATGTTGACCTTATTTAATCAATATAAATCACGTAGTGTGCTTCTTTGTGGGCAACAAATGCTTGATGAAGTCAACAGTGCAATAGAAAAAGTAGCAAACACGTTGTCAGCTCCTTTATCTTTAGAACAGCACTGTGATGTTATGGATGTTGAAGATAATATTGAACAAAAGCAGAACTCAGCATTAGAAAAATTTGTAGTAAATAATATCCTTCAAGGCTATAAAGAAGTTGCTAATGTTTATGAGGAATTACAAAATAACGCTACCTTACCTTACTCTTGAAGAGCAAAATTCAGCAGCATTAAGTGCTTGGAAAGATCTGATTAAACAAAAATCCGATTTAGATAGCCAAAAATATACCATTAAAAAGAATAAAGATCGAATAGCTGAATTAATTTCTTCAACAACAGAGTTGTCTGAGAGATTAAATACAGCACAAAATTCATTTTCATCACCAAAAGCAAATCTAGAACTCAAAAATCATCAATTACAAGACGATTTAGAAAATAGTCAAGAACTTTCTAGACAGCTAGAAAATATACAAGAACAAACCACTAGAATTATAACTGAAAAAGAAAA

**References:**

1.	Bell, J.R., *A simple way to treat PCR products prior to sequencing using ExoSAP-IT®.* Biotechniques, 2008. **44**(6): p. 834.
