# Supplementary material for: Comparative genomics and in silico virulence assessment of nonpathogenic Avibacterium paragallinarum isolates from healthy layer chickens
Source: Front Microbiol. 2026 Sep 3;17:1919963. doi: 10.3389/fmicb.2026.1919963 (PMC13582619; doi:10.3389/fmicb.2026.1919963)
Supplement: Supplementary file 9 [file Table_9.docx]

**Virulence Factors Analysis**

1. **Identified from Avibacterium paragallinarum/Pasteurellaceae literature**
   1.1. **Lipooligosaccharide**

Two genes, waaF and waaQ, were investigated in this study. Both encode heptosyltransferases involved in the assembly of the inner core of lipooligosaccharide. The waaF gene was identified in the analyzed genomes [1], with a BV-BRC ID of **fig|728.107.peg.2097 as query sequence**. The waaQ gene was identified based on sequence homology with Pasteurella multocida waaQ [2, 3]. The WaaQ protein in AP genomes showed 63–64% amino acid identity to the corresponding Pasteurella multocida protein, with a BV-BRC ID of **fig|728.107.peg.2126**.

Genomic analysis revealed the presence of the waaF gene in all analyzed pAP and npAP genomes. The in silico translated ORFs varied in length among genomes. Two npAP genomes, npAP/GA-USA/20231220/S2-3 and npAP/GA-USA/20231220/S2-4, encoded the longest WaaF protein with 369 amino acids, compared to 348 amino acids in the remaining npAP genomes and 348–366 amino acids in the pAP genomes. Notably, the waaF gene was incomplete in one pAP genome (JF4211).

Nine putative active sites (G186, P187, F219, A255, V256, N268, G271, L272, and V275) were identified and found to be highly conserved between pAP and npAP, except for position G186, which was substituted with cysteine (C) in all npAP genomes and the majority of pAP genomes. Protein domain analysis revealed the presence of the Glycosyltransferase family 9 (heptosyltransferase) domain (IPR002201) in both pAP and npAP.

Regarding the waaQ gene, it was present in all analyzed genomes with an identical translated ORF length of 339 amino acids. Multiple sequence alignment revealed seven amino acid substitutions in some npAP genomes compared to pAP (Supplementary File 2B). The same Glycosyltransferase family 9 (heptosyltransferase) domain (IPR002201) was identified in both pAP and npAP.

The *lic1ABCD* operon, which regulates the phase-variable expression of phosphorylcholine on the lipooligosaccharide of AP [4], was also examined in this study. Our analysis showed that the *lic1ABCD* operon is not universally present across AP genomes, as it was absent in several pAP genomes, including ESV-135, AVPG2015, FARPER, ADL, and the five field isolates. Among the npAP isolates, the operon was detected only in npAP/GA-USA/2023 1216/S1-1, npAP/GA-USA/20231216/S1-2, npAP/GA-USA/20231220/S2-1, npAP/GA-USA/20 231220/S2-2, and npAP/OH-USA/20230315/S2-1. The Georgia npAP isolates carried the complete operon, except for npAP/GA-USA/20231216/S1-2, which contains a deletion in *lic1B* at position 363 (based on the npAP/GA-USA/20231216/S1-1 numbering) that introduces a premature stop codon. A similar disruption was noted in npAP/OH-USA/20230315/S2-1, which contains a cysteine insertion at positions 172/173 in *lic1B* and also lacks *lic1D*. It is noteworthy that the type strain also exhibits a split *lic1B* ORF caused by a deletion at position 357. Additionally, tetranucleotide CAAT repeats were identified within *lic1A* of the npAP isolates.

1.2. RTX toxin

The ***avxCABD* operon**, which encodes a dual-function serine protease–RTX toxin, was investigated [5]. avxA encodes the pretoxin that is activated by the product of avxC and exported via the type I secretion system encoded by avxBD. The BV-BRC IDs of this operon are fig|728.107.peg.1101 (avxC), fig|728.107.peg.1099 (avxA), fig|728.107.peg.1098 (avxB), and fig|728.107.peg.1097 (avxD). The analysis uncovered defects in the RTX operon of some pAP genomes and revealed its presence only in three npAP isolates: AG21-0333, npAP/OH-USA/20230315/S2-1, and npAP/FL-USA/20231025/S1-1.

avxA analysis revealed nine active sites (E980, F1013, H1016, W1067, S1086, N1117, S1128, S1197) and three catalytic sites (E980, H1016, S1197), all of which were conserved between pAP and npAP. Protein domain analysis indicated the presence of RTX calcium-binding nonapeptide repeat domains (IPR001343), cadherin-like domain (IPR041690), serine peptidase domain (IPR000209), P/Homo-B domain (IPR002884), and hemolysin-type calcium-binding protein domain (IPR010566) in both pAP and npAP. Multiple sequence alignment demonstrated that approximately the first ~1000 nucleotides (~333 amino acids) are highly variable among the genomes included in the study, regardless of pathotype. Gene length was variable as well, with 2194 and 2286 amino acids being the most frequently reported lengths. Notably, the pAP ZJ-C genome had multiple insertions and deletions that interrupted the ORFs of the entire operon and introduced multiple premature stop codons. Furthermore, the pAP ADL-AP10 genome had an adenine insertion at position 1292/1293, which resulted in a premature stop codon.

The acylation site (GK) and glycine-rich nonapeptide repeats described by Küng and Frey [5] were examined. The analysis identified the acylation site (GK) at positions 1538/39 and 16 glycine-rich repeats (Supplementary File 2C) according to Satchell’s guidelines [6] in the three npAP AvxA proteins, similar to pAP genomes.

Regarding the exporters AvxB and AvxD, no active sites were identified. Protein domain analysis revealed the presence of the peptidase C39 family (IPR005074), ABC transporter transmembrane region (IPR011527), and ABC transporter domain (IPR003439) in AvxB of both pAP and npAP. Meanwhile, AvxD contained the CyaD alpha-helical hairpin domain (IPR059040) and AprE-like beta-barrel domain (IPR058982) in both pAP and npAP.

The avxC, activator-encoding gene, was investigated in the complete pAP genomes and the three npAP genomes. No active sites or protein domains were identified. Significant length variation was observed and appeared to correlate with geographic origin. For example, the Chinese genomes C-AP4, C-AP1, and p4chr1 had truncated activators of 83 amino acids compared to the 144-amino-acid activator of the type strain (85% of the short protein aligned with 100% identity to NCTC11296), whereas genomes from the Americas, such as ADL-AP and FARPER-174, contained extended homologs of 732 amino acids (17.8% of the long protein aligned with 96% identity to NCTC11296). In the npAP genomes, homologs of 143 amino acids were detected in npAP/OH-USA/20230315/S2-1 (54.5% aligned with 93.7% identity to NCTC11296) and 83 amino acids in npAP/FL-USA/20231025/S1-1 (90% aligned with 84.42% identity to NCTC11296), while no homolog was identified in AG21-0333. An interesting observation in AG21-0333 was that the AvxC of the type strain showed 100% identity to the first 126 amino acids of AvxA in this isolate. Similar observations were made in the AvxA sequences of AVP2015, ADL-AP, FARPER, and npAP/FL-USA/20231025/S1-1 genomes.

1.3. **Cytolethal distending toxin (CDT)**

The CDT is a cytotoxin encoded by an operon composed of three genes, *cdtA*, *cdtB*, and *cdtC* [7]. The *cdtB* gene encodes the active toxin with DNase I-like activity (CdtB), whereas *cdtA* and *cdtC* encode proteins (CdtA, CdtC) responsible for binding and delivery of CdtB toxin into the cell.

In the present study, genomic analysis revealed that the CDT operon was present and highly conserved across all pAP and npAP genomes. The BV-BRC IDs of this are fig|728.107.peg.2915 (cdtA), fig|728.107.peg.2914 (cdtB), and fig|728.107.peg.2913 (cdtC). Using the NCBI CDD, several putative catalytic, metal-binding, and phosphate-binding sites were identified, along with the predicted interfaces of the tripartite CDT complex. The putative catalytic (N31, E64, H158, D194, N196, D234, D265, H266), metal-binding (E64, D265), and phosphate-binding sites (H158, N196, H266) were conserved among both pAP and npAP genomes. Furthermore, five experimentally verified catalytic and metal-binding residues (E90, H158, D234, D265, and H266) [8] were conserved between pAP and npAP.

In terms of the tripartite complex interfaces, specifically the CdtC trimer interface, two amino acid substitutions were observed in npAP compared to pAP: S70R in npAP/GA-USA/20231220/S2-3, npAP/GA-USA/20231220/S2-4, npAP/NC-USA/20240110/S1-1, and npAP/FL-USA/20231025/S1-1; and P174S in AG21-0333, npAP/OH-USA/20230213/S1-2, npAP/OH-USA/20230213/S1-3, and npAP/OH-USA/20230213 /S1-4.

Protein domain analysis revealed that the same domains were present in both npAP and pAP genomes: the Endonuclease/Exonuclease/Phosphatase family (IPR005135) for CdtB, and the Cytolethal Distending Toxin A/C domain (IPR003558) for both CdtA and CdtC.

1.4. **F17-like fimbriae**

The bacterial fimbriae are composed of four structural proteins: the major fimbrial subunit, the chaperone, the usher, and the tip adhesin [9] and are encoded by the *flfADCG* operon in AP [10]. In this study, the major fimbrial subunit encoding gene (*flfA*) (BV-BRC ID: fig|728.107.peg.459) was investigated as a virulence factor according to Bager et al., [11] and Liu et al., [10].

The analysis revealed that FlfA is not present in all AP genomes. It is absent in some pAP genomes including Hp8, SA-3, Modesto, 2671, and NCTC10926. The FlfA is present in all npAP except for AP-2, AG21-0333, and npAP/NC-USA/20240110/S1-1.

In term of amino acids identity, the FlfA sequence of strain TW07 (GenBank accession number KU757292) [10] was used as a reference. The majority of pAP FlfA sequences showed 100% amino acid identity to TW07 FlfA, including the type strain. Among npAP FlfA, the Ohio and Florida isolates showed 99-100% amino acid identity to pAP FlfA. On contrary, the Georgia isolates npAP/GA-USA/20231216/S1-1, npAP/GA-USA/20231216/S1-2, npAP/GA-USA/20231220/S2-1, and npAP/GA-USA/20231220/S2-2 showed 76% amino acid identity, while npAP/GA-USA/20231220/S2-3 and npAP/GA-USA/20231220/S2-4 showed amino acid 68% identity to pAP.

Protein domain analysis identified the presence of Type-1 fimbrial protein A domain (IPR039458) in both pAP and npAP. The presence of poly-C repeats in the putative promoter region of *flfA,* which may control the phase-variable expression of fimbriae, was also investigated in npAP according to Liu et al., [10]. Ten to thirteen C repeats were identified in the putative promoter region of *flfA* in npAP, except for npAP/FL-USA/20231025/S1-1, in which the sequence was incomplete (Supplementary File 2D).

1.5. **Heme utilization system**

*hutZ* (BV-BRC ID: fig|728.107.peg.1183), *hutX (*BV-BRC ID: fig|728.107.peg.1182*)*, TonB dependent heme receptor A (BV-BRC ID: fig|728.106.peg.298), and Fur protein (BV-BRC ID: fig|728.107.peg.1281) were investigated.

The analysis revealed that *hutZ* is present in all pAP and npAP, and it is highly conserved except for minor substitutions were reported. Protein domain analysis identified the presence of Pyridoxamine 5’- phosphate oxidase domain (IPR011576) in both pAP and npAP.

*hutX* is present in all pAP and npAP, but it is less conserved compared to *hutZ*. The protein domain Haem utilization ChuX/HutX (IPR010413) was present in both pAP and npAP.

TonB dependent heme receptor A has two domains named TonB-dependent receptor plug domain (IPR012910), and TonB-dependent receptor like, beta barrel (IPR000531) and both are present in pAP and npAP. This protein is shorter in npAP compared to pAP by 9 amino acids except for npAP/FL-USA/20231025/S1-1, npAP/GA-USA/20231220/S2-2, npAP/GA-USA/20231220/S2-3, and npAP/GA-USA/20231220/S2-4.

It was noticed that the 10 npAP isolates of shorter protein has a highly variable region in TonB-dependent receptor like, beta barrel domain (IPR000531) at positions 376-403 amino acids as shown below.

Regarding amino acid identity %, it is 98-100% among pAP compared to the type strain, while 98% in npAP/GA-USA/20231220/S2-2, npAP/GA-USA/20231220/S2-3, and npAP/GA-USA/20231220/S2-4; 97% in npAP/NC-USA/20240110/S1-1; 96% in npAP/IA-USA/20210120/S1-1 (AP-2), npAP/OH-USA/20230213/S1-2, npAP/OH-USA/20230315/S2-1, npAP/GA-USA/20231216/S1-1, npAP/GA-USA/20231216/S1-2, and npAP/GA-USA/20231220/S2-1; and 95% in npAP/OH-USA/20210309/S1-1 (AG21-0333), npAP/OH-USA/20230213/S1-3, and npAP/OH-USA/20230213/S1-4.

The ferric uptake regulation protein FUR is present in all npAP and highly conserved. Protein domain analysis revealed the presence of Ferric uptake regulator family (IPR002481).

Our interpretation of these results is that the iron acquisition system in npAP should be functional. However, the allelic divergence observed in the majority of npAP strains within the TonB-dependent heme receptor A should be further investigated, as it may alter iron uptake and utilization in npAP, but at this moment we do not know.

1.6. **Bis (5’-nucleosyl)-tetraphosphatase symmetrical (EC 3.6.1.41) – Metallophosphatase**

The metallophosphatase encoding gene (*apaH*) (BV-BRC ID: fig|728.107.peg.1709) was investigated based on the findings of Guo et al., [12]. The analysis showed that *apaH* is present in all pAP and npAP genomes, with a translated protein length of 273 amino acids. No putative active sites were identified using NCBI CDD tool. A unique amino acid substitution (Q105R) was observed in all npAP genomes compared to pAP, except for AP-2, npAP/GA-USA/20231220/S2-3, and npAP/GA-USA/20231220/S2-4. Protein domain analysis identified the presence of a calcineurin-like phosphoesterase domain (IPR004843) in both pAP and npAP genomes.

1.7. **16S rRNA adenine dimethyltransferase (EC 2.1.1.182)**

The 16S rRNA adenine dimethyltransferase encoding gene (*ksgA/rsmA*) (BV-BRC ID: fig|728.107.peg.1708) is important for pAP virulence [13]. Our analysis showed that *ksgA/rsmA* is present in all pAP and npAP genomes. The npAP/FL-USA/20231025/S1-1 has incomplete gene because of incomplete sequence. No putative active sites were identified using NCBI CDD tool. A unique amino acid substitution (A278T) was observed in all npAP genomes compared to pAP, except for npAP/GA-USA/20231216/S1-1, npAP/GA-USA/20231216/S1-2, npAP/GA-USA/20231220/S2-1, npAP/GA-USA/20231220/S2-2, and npAP/NC-USA/20240110/S1-1. Protein domain analysis identified the presence of a ribosomal RNA adenine methyltransferase KsgA/Erm domain (IPR001737) in both pAP and npAP genomes.

1.8. **Metalloprotease**

The metalloprotease-encoding gene (*pepN*) (BV-BRC ID: fig|728.107.peg.438) was investigated based on previous studies in AP [14] and *Actinobacillus pleuropneumoniae* [15]. The *pepN* gene was identified in the analyzed AP genomes through sequence homology search against the corresponding sequence in *Actinobacillus pleuropneumoniae* [15], showing 69% amino acid identity to AP Metalloprotease.

The analysis confirmed the presence of *pepN* gene in all pAP and npAP. Notably, *pepN* was fragmented into three segments in the AV32 pAP genome. Metalloprotease sequences have the same size of 869 amino acids across all genomes except for AG21-0333, which has two additional amino acids at position 86, resulting in a total length of 871 amino acids.

Three Zinc binding sites (H298, H302, and E32), and 19 active sites (Q120, E122, 259FNMGAME, V295, H298, E299, H302, K320, E321, N374, Y377, Y382, E383) were identified and found to be conserved in both pAP and npAP. Protein domain analysis showed similar hits in both pAP and npAP to Peptidase M1 N-terminal domain (IPR045357), Peptidase family M1 domain (IPR014782), Domain of unknown function (DUF3458) Ig-like fold (IPR035414), and Domain of unknown function (DUF3458_C) ARM repeats (IPR024601).

1.9. **Glutathione reductase (EC 1.8.1.7)**

The Glutathione reductase-encoding gene (*gor*) (BV-BRC ID: fig|728.107.peg.1672) was investigated based on the findings of Zhi et al., [16]. No putative active sites were identified using the NCBI CDD tool. Some amino acid substitutions were observed in the npAP genomes compared to pAP (Supplementary File 2F). Protein domain analysis identified the presence of the Pyridine nucleotide-disulphide oxidoreductase domain (IPR023753) and the Pyridine nucleotide-disulphide oxidoreductase dimerisation domain (IPR004099) in both pAP and npAP genomes.

2. **Identified from VFDB**
   2.1. ***kdsA***

The ***kdsA*** gene, which encodes **2-Keto-3-deoxy-D-manno-octulosonate-8-phosphate synthase** (EC 2.5.1.55) (BV-BRC ID: **fig|728.107.peg.641)**, was investigated. The analysis revealed that the kdsA gene was present in all pAP and npAP genomes included. A defect was identified in the kdsA gene of the nonpathogenic isolate **npAP/OH-USA/20230315/S2-1**, caused by the insertion of a guanosine nucleotide at position 140, leading to the introduction of a premature stop codon. No active sites were identified via NCBI CDD analysis. Protein domain analysis revealed the presence of the **DAHP synthetase I family domain (IPR006218)** in both pAP and npAP. Notably, the *kdsA* gene in the pAP CCUG 12835 genome was remarkably longer than in all other genomes (almost twice the length) and contained two additional protein domains: the transglutaminase-like superfamily (IPR032698) and the tetratricopeptide repeat (PF13371).

2.2. ***rfaD***

The *rfaD* gene, which encodes ADP-L-glycero-D-manno-heptose-6-epimerase (EC 5.1.3.20) (BV-BRC ID: **fig|728.107.peg.2098)**, was present in all pAP and npAP genomes included.

The NCBI CDD analysis determined four active sites (N93 – S116 – Y139 – K143), 27 NADP binding sites (T5 – G6 - G9 – F10 – I11 – 30VDD – K38 – K53 – 75EGA – S79 – F88 – N92 – Y96 – 114ASS – Y139 – K143 – 166YF – V169 – 176HK), and nine substrate binding sites (T81 – N168 – 179SMA – V183 – H186 – R208 – F242), and all are conserved between pAP and npAP. Protein domain analysis revealed the presence of the NAD dependent epimerase/dehydratase family **domain (**IPR001509**)** in both pAP and npAP.

2.3. ***IpxA***

The *IpxA* gene, which encodes Acyl-[acyl-carrier-protein]--UDP-N-acetylglucosamine O-acyltransferase (EC 2.3.1.129) (BV-BRC ID: **fig|728.107.peg.1718)**, was present in all pAP and npAP genomes included. No active sites were identified via NCBI CDD analysis. Protein domain analysis revealed the presence of GMPPB C-terminal domain (IPR056729), bacterial transferase hexapeptide domain (IPR001451), and Udp N-acetylglucosamine O-acyltransferase Domain 2 (IPR029098) in both pAP and npAP.

2.4. ***KatA***

The *KatA* gene, which encodes Catalase KatE (EC 1.11.1.6), was only present in four nonpathogenic isolates among all AP genomes included. The four isolates are AG21-0333, npAP/OH-USA/20230213/S1-2, npAP/ OH-USA/20230213/S1-3, and npAP/OH-USA/20230213/S1-4. The BV-BRC ID of *KatA* in AG21-0333 isolate is fig|728.352.peg.1751.

2.5. ***rfaE***

The *rfaE* gene, which encodes D-glycero-beta-D-manno-heptose 1-phosphate adenylyltransferase (EC 2.7.7.70)/D-glycero-beta-D-manno-heptose-7-phosphate kinase (EC 2.7.1.167) (BV-BRC ID: fig|728.107.peg.700), was present in all pAP and npAP genomes included. No active sites were identified via NCBI CDD analysis. Protein domain analysis revealed the presence of pfkB family carbohydrate kinase domain (IPR011611), and Cytidylyltransferase-like domain (IPR004821) in both pAP and npAP.

2.6. ***gmhA/lpcA***

The *gmhA/lpcA* gene, which encodes D-sedoheptulose 7-phosphate isomerase (EC 5.3.1.28) (BV-BRC ID: fig|728.107.peg.1923), was present in all pAP and npAP genomes included. Nine active sites (52NGG – 119STS – S124 – Y167 –Q172) were identified via NCBI CDD, and these sites were conserved between pAP and npAP. Protein domain analysis revealed the presence of SIS domain (IPR001347) in both pAP and npAP.

2.7. ***gmd***

The *gmd* gene, which encodes GDP-mannose 4,6-dehydratase (EC 4.2.1.47) (BV-BRC ID: fig|728.107.peg.2586) was not present in all AP genomes included. Specifically, the gene was absent in six pAP genomes: HP60, HP8, SA-3, Modesto, 2671, and NCTC10926. In the case of npAP, the *gmd* gene was disrupted in npAP/OH-USA/20230213/S1-4 due to the insertion of four nucleotides (AAGA) at position 594, resulting in a frameshift and the introduction of a premature stop codon. No active sites were identified via NCBI CDD analysis. Protein domain analysis revealed the presence of GDP-mannose, 6 dehydratase domain (IPR016040) in both pAP and npAP.

2.8. ***msbB***

The *msbB* gene*,* which encodes lipid A biosynthesis myristoyltransferase (EC 2.3.1.243) (BV-BRC ID: fig|728.107.peg.1291), was present in all pAP and npAP genomes included. The BV-BRC ID of the *msbB* gene in the AP type strain is. A putative acyl-acceptor binding pocket consisting of ten sites (144H -147A – 149D -166YNPH – 215EDY) was identified via NCBI CDD, and it was conserved between pAP and npAP. Protein domain analysis revealed the presence of bacterial lipid A biosynthesis acyltransferase domain (IPR004960) in both pAP and npAP.

2.9. ***lpxB***

The *lpxB* gene, which encodes lipid-A-disaccharide synthase (EC 2.4.1.182) (BV-BRC ID: fig|728.107.peg.1717), was present in all pAP and npAP genomes included. No active sites were identified via NCBI CDD analysis. Protein domain analysis revealed the presence of lipid-A-disaccharide synthetase domain (IPR003835) in both pAP and npAP.

2.10. ***lpxC***

The *lpxC* gene, which encodes UDP-3-O-[3-hydroxymyristoyl] N-acetylglucosamine deacetylase (EC 3.5.1.108) (BV-BRC ID: fig|728.107.peg.987), was present in all pAP and npAP genomes included. No active sites were identified via NCBI CDD analysis. Protein domain analysis revealed the presence of UDP-3-O-acyl N-acetylglycosamine deacetylase domain (IPR004463) in both pAP and npAP.

2.11. ***lpxD***

The *lpxD* gene, which encodes UDP-3-O-[3-hydroxymyristoyl] glucosamine N-acyltransferase (EC 2.3.1.191) (BV-BRC ID: fig|728.107.peg.1720), was present in all pAP and npAP genomes included. No active sites were identified via NCBI CDD analysis. Protein domain analysis revealed the presence of UDP-3-O-[3-hydroxmyristoyl] glucosamine N-acyltransferase, LpxD domain (IPR020573), and two Bacterial transferase hexapeptide (IPR001451) in both pAP and npAP.

2.12. ***galE***

The *galE* gene, which encodes UDP-glucose 4-epimerase (EC 5.1.3.2) (BV-BRC ID: fig|728.107.peg.2246), was present in all pAP and npAP genomes included. No active sites were identified via NCBI CDD analysis. Protein domain analysis revealed the presence of NAD dependent epimerase/dehydratase family domain (IPR001509) in both pAP and npAP.

2.13. ***gaIU***

The *gaIU* gene, which encodes UTP--glucose-1-phosphate uridylyltransferase (EC 2.7.7.9) (BV-BRC ID: fig|728.107.peg.2980), was present in all pAP and npAP genomes included. No active sites were identified via NCBI CDD analysis. Protein domain analysis revealed the presence of Nucleotidyl transferase (IPR005835) in both pAP and npAP.

2.14. *wecA*

The *wecA* gene, which encodes undecaprenyl-phosphate alpha-N-acetylglucosaminyl 1-phosphate transferase (EC 2.7.8.33) (BV-BRC ID: fig|728.107.peg.829), was present in all pAP and npAP genomes included. No active sites were identified via NCBI CDD analysis. Protein domain analysis revealed the presence of Glycosyl transferase family 4 (IPR000715) in both pAP and npAP.

The summary of the analysis findings for the virulence-associated genes identified via VFDB is shown (Supplementary Table 3).

**Discussion**

Beyond the four consistent findings, several additional trends merit discussion for their potential value to the AP literature. For example, RTX is not strictly absent from npAP and present only in pAP: we detected RTX operons in three npAP isolates and observed defects in *avxA*, the gene encoding the RTX pretoxin, in some pAP genomes. The *avxA* defects reported in this study in the highly virulent pAP strain ZJ-C [17], and in the pathogenic ADL-AP10 strain [18], could reflect sequencing or assembly errors, or they could represent true biological mutations that render the toxin nonfunctional. Consequently, RTX production by ZJ-C and ADL-AP10 should be confirmed experimentally. These observations raise an important question: is RTX a major virulence determinant in AP? Küng and Frey [5] observed RTX-associated cytotoxicity and concluded that RTX is a common major virulence factor across AP serotypes, whereas Pan *et al.* in 2012 [19] did not reproduce this finding. This discrepancy, which may be attributable to differences in tissue culture systems or other experimental conditions, underscores the need for further, carefully controlled studies to clarify the role of RTX in AP pathogenicity.

Another noteworthy observation involves AvxC, the activator of the RTX pretoxin. The AG21-0333 isolate does not appear to possess a full-length *AvxC* gene; instead, only a partial match corresponding to the N-terminus of the AvxA pretoxin was detected. This pattern also occurs in several pAP isolates (ADL-AP, FARPER, and AVPG2015) as well as in npAP/FL-USA/20231025/S1-1. This raises an additional question: could AvxC be structurally integrated into AvxA in certain isolates rather than encoded as a separate activator gene? At the complete-genome level, we further noted truncated AvxC homologs of 83 amino acids in C-AP4, C-AP1, and p4chr1, and extended homologs of 732 amino acids in ADL-AP and FARPER-174, compared to the type strain. Whether these homologs are functional, or whether AvxC is universally required for AP isolates to produce an active RTX toxin, remains unclear. Küng and Frey [5] demonstrated that AvxC was necessary for RTX activity in the AP isolates included in their study. However, evidence from other bacterial systems suggests that activator proteins are not universally required for toxin functionality, especially in the Multifunctional Autoprocessing Repeats-in-Toxin Toxins (MARTX) [6]. Successful disruption of *V. vulnificus* *rtxC* was reported and found to have no effect on *rtxA* cytotoxicity, despite the fact that disruption of *rtxA* caused loss of cytotoxicity and decreased the bacterial virulence [20], but more experimental data are needed to resolve this question in AP.

Virulence-associated protein D (VapD) has been characterized as a virulence factor in nontypeable *Haemophilus influenzae* [21, 22], and *Riemerella anatipestifer* [23]. However, its role in AP remains unclear, as no studies have investigated VapD in this species. In our dataset, VapD was present in all pAP genomes and absent in all npAP genomes, except for two npAP isolates. This pattern suggests that VapD may function as a minor or auxiliary virulence factor in AP, warranting further investigation.

The ***lic1ABCD* operon**, which regulates phase-variable expression of phosphorylcholine on the lipooligosaccharide of AP [4] was also analyzed as a potential virulence factor in this study. Our analysis showed that this operon is absent in several pAP genomes, indicating that it is unlikely to represent a major virulence determinant for AP. Furthermore, its presence in some npAP isolates, along with the tetranucleotide CAAT repeats in *lic1A* that control phosphorylcholine expression as described for pAP [4] and *Haemophilus influenzae* [24], further supports the interpretation that *lic1ABCD* is not a major contributor to AP pathogenicity.

The F17-like fimbriae encoded by *flfADCG* operon [10] were investigated in this study, with particular focus on the major fimbrial subunit gene *flfA* as a proposed virulence factor for AP [10]. The analysis showed that *flfA* is absent in some pAP genomes yet present in the majority of npAP isolates. The Georgia npAP FlfA proteins displayed lower pairwise identities compared to pAP; however, they may still fall within the same major fimbrial subunit category based on the 66.7% identity threshold defined by Kudirkienė *et al.* in 2014 [25] for *Gallibacterium* species. The poly-C repeats in the putative promoter region of *flfA*, which may regulate phase-variable fimbrial expression in AP, were also examined in npAP following the framework proposed by Liu *et al.* in 2016 [10]. Ten to thirteen consecutive C repeats were detected in npAP, suggesting the possibility of relatively high flfA expression, similar to the hypothesis proposed [10] for pAP*.* Given its absence in some pAP, its presence in most npAP, and the suggestion that these repeats may lead to higher expression in npAP, FlfA is unlikely to function as a common major virulence factor in AP.

It is also worth noting that all virulence factors identified through VFDB cannot be considered major virulence determinants of AP based on our analysis, as they are present, and appear functional, in npAP isolates. Therefore, these factors are more likely to represent minor or accessory virulence elements rather than major virulence determinants of AP.

**References**

1.	Chen, L., et al., *Identification and characterization of biosynthetic loci of lipooligosaccharide and capsular polysaccharide in Avibacterium paragallinarum.* Veterinary Microbiology, 2024. **299**: p. 110317.

2.	Harper, M., et al., *Signature-tagged mutagenesis of Pasteurella multocida identifies mutants displaying differential virulence characteristics in mice and chickens.* Infection and immunity, 2003. **71**(9): p. 5440-5446.

3.	Harper, M., et al., *A heptosyltransferase mutant of Pasteurella multocida produces a truncated lipopolysaccharide structure and is attenuated in virulence.* Infection and immunity, 2004. **72**(6): p. 3436-3443.

4.	Chiang, Y., et al., *Identification of the lic1ABCD operon that controls the phase-variable expression of phosphorylcholine on lipopolysaccharide from Avibacterium paragallinarum.* Avian Pathology, 2013. **42**(1): p. 72-78.

5.	Küng, E. and J. Frey, *AvxA, a composite serine-protease-RTX toxin of Avibacterium paragallinarum.* Veterinary microbiology, 2013. **163**(3-4): p. 290-298.

6.	Satchell, K.J.F., *MARTX, multifunctional autoprocessing repeats-in-toxin toxins.* Infection and immunity, 2007. **75**(11): p. 5079-5084.

7.	Lara-Tejero, M.a. and J.E. Galán, *Cytolethal distending toxin: limited damage as a strategy to modulate cellular functions.* Trends in microbiology, 2002. **10**(3): p. 147-152.

8.	Elwell, C.A. and L.A. Dreyfus, *DNase I homologous residues in CdtB are critical for cytolethal distending toxin‐mediated cell cycle arrest.* Molecular microbiology, 2000. **37**(4): p. 952-963.

9.	Wurpel, D.J., et al., *Chaperone-usher fimbriae of Escherichia coli.* PloS one, 2013. **8**(1): p. e52835.

10.	Liu, C.-C., et al., *The fimbrial protein is a virulence factor and potential vaccine antigen of Avibacterium paragallinarum.* Avian Diseases, 2016. **60**(3): p. 649-655.

11.	Bager, R.J., et al., *The fimbrial protein FlfA from Gallibacterium anatis is a virulence factor and vaccine candidate.* Infection and immunity, 2013. **81**(6): p. 1964-1973.

12.	Guo, M., et al., *Identifcation of the genes involved in biofilm formation of Avibacterium paragallinarum using random transposon mutagenesis.* Veterinary Microbiology, 2025. **302**: p. 110410.

13.	Guo, M., et al., *Development and evaluation of an attenuated Avibacterium paragallinarum strain as a live vaccine candidate for infectious coryza.* Veterinary Research, 2025. **56**(1): p. 115.

14.	Rivero-García, P., et al., *Haemophilus paragallinarum secretes metalloproteases.* Canadian journal of microbiology, 2005. **51**(10): p. 893-896.

15.	González, O.G.a., et al., *Actinobacillus pleuropneumoniae metalloprotease: cloning and in vivo expression.* FEMS Microbiology Letters, 2004. **234**(1): p. 81-86.

16.	Zhi, Y., et al., *Glutathione reductase modulates endogenous oxidative stress and affects growth and virulence in Avibacterium paragallinarum.* Veterinary research, 2025. **56**(1): p. 1.

17.	Mei, C., et al., *Characterization of a highly virulent Avibacterium paragallinarum isolate.* Journal of Animal Science, 2023. **101**: p. skad365.

18.	Byukusenge, M., et al., *Complete genome sequences of seven Avibacterium paragallinarum isolates from poultry farms in Pennsylvania, USA.* Microbiology Resource Announcements, 2020. **9**(27): p. 10.1128/mra. 00654-20.

19.	Pan, Y.-C., et al., *Identification and characterization of an RTX toxin–like gene and its operon from Avibacterium paragallinarum.* Avian Diseases, 2012. **56**(3): p. 537-544.

20.	Joseph, J. and P. Gulig, *Analysis of the three RTX loci in pathogenesis of Vibrio vulnificus, abstr. B-035. Abstr. 107th Gen.* Meet. Am. Soc. Microbiol. American Society for Microbiology, Washington, DC, 2007.

21.	Daines, D.A., J. Jarisch, and A.L. Smith, *Identification and characterization of a nontypeable Haemophilus influenzae putative toxin-antitoxin locus.* BMC microbiology, 2004. **4**(1): p. 30.

22.	Ren, D., A.N. Walker, and D.A. Daines, *Toxin-antitoxin loci vapBC-1 and vapXD contribute to survival and virulence in nontypeable Haemophilus influenzae.* BMC microbiology, 2012. **12**(1): p. 263.

23.	Chang, C., P. Hung, and Y. Chang, *Molecular characterization of a plasmid isolated from Riemerella anatipestifer.* Avian pathology, 1998. **27**(4): p. 339-345.

24.	De Bolle, X., et al., *The length of a tetranucleotide repeat tract in Haemophilus influenzae determines the phase variation rate of a gene with homology to type III DNA methyltransferases.* Molecular microbiology, 2000. **35**(1): p. 211-222.

25.	Kudirkienė, E., et al., *Chaperone-usher fimbriae in a diverse selection of Gallibacterium genomes.* BMC genomics, 2014. **15**(1): p. 1093.
